# Coordination-Induced Weakening of N–H Bonds Driven by Bimetallic Cooperativity in Zr/Co Compounds

**DOI:** 10.1021/acs.inorgchem.5c05652

**Published:** 2026-01-27

**Authors:** Julia Feresin, Megan. C. Ford, Matthew K. Vascura, Curtis E. Moore, Seth M. Barrett, Christine M. Thomas

**Affiliations:** † Department of Chemistry and Biochemistry, 2647 The Ohio State University, 100 W. 18th Avenue, Columbus, Ohio 43210, United States; ‡ Department of Chemistry, 5753 Muskingum University, 260 Stadium Drive, New Concord, Ohio 43762, United States

## Abstract

The bond dissociation free energy (BDFE) of the element-hydrogen bonds of protic substrates have been found to decrease upon metal coordination. Herein, an early/late heterobimetallic complex is used to examine the impact on the BDFE_N–H_ when the substrate binding site and the redox-active site are two different metals that are spatially separated. A tris­(phosphinoamide) framework is used to link a d^0^ Zr^IV^ center with an accessible substrate binding site to a coordinatively saturated redox-active Co center, which serves as an appended electron reservoir. A series of aniline, amido, and imido Zr/Co model compounds were synthesized starting from the Zr^IV^/Co^–I^ aniline adduct PhH_2_N–Zr­(MesNP^*i*^Pr_2_)_3_CoCN^*t*^Bu (**2**). 2,4,6-tris-*tert*-butylphenoxyl radical (^*t*^Bu_3_ArO^•^) was used to abstract one or two H atoms and produce the amido and imido complexes PhHN-Zr­(MesNP^*i*^Pr_2_)_3_CoCN^*t*^Bu (**3**) and PhN≡Zr­(MesNP^*i*^Pr_2_)_3_CoCN^*t*^Bu (**4**), respectively. Using open-circuit potential measurements, the BDFE_N–H_ within **2** and **3** were determined to be 37 kcal/mol (**2**) and 55 kcal/mol (**3**). Cyclic voltammetry measurements were conducted to determine the Co^I/0^ and Co^0/–I^ redox potentials. The p*K*_a_s were then estimated using the Bordwell equation to provide further insight into the thermochemical aspects of the observed proton coupled electron transfer (PCET) reactions.

## Graphical abstract

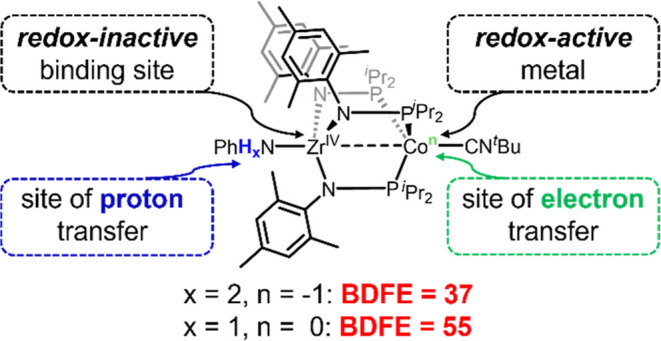

## Introduction

The Lewis acidity and redox activity of metal centers combine to substantially weaken the element-hydrogen bonds of metal-bound substrates. This so-called coordination-induced bond weakening is thought to play an important role in both organic transformations and the catalytic oxidation of small molecules such as water and ammonia.^1,2^ For example, recent reports on molecular catalysts for ammonia oxidation have highlighted the role of coordination-induced bond weakening as an approach to access N–H bond cleavage through a series of sequential oxidation, deprotonation, or hydrogen atom transfer steps.^1,3^ Researchers have reported many examples of BDFE_N–H_ decreasing upon amine coordination to a metal center, with the N–H bonds becoming weaker as the metal oxidation state decreases.^1,4,5^ However, there have been only a few examples of coordination-induced bond weakening in which the BDFE of an N–H bond decreases to lower than 50 kcal/mol. Among these examples, Chirik and co-workers reported Mo–NH_3_ and titanocene–NH_3_ complexes in which the BDFE_N–H_ is 45.8 and 38.4 kcal/mol, respectively (Figure 1A).^2,6^ Similarly, Ramírez-Solis, Flowers, and co-workers estimated that coordination of NH_3_ to SmI_2_ results in a remarkable decrease in the BDFE_N–H_ to 34 kcal/mol (Figure 1A).^7^ Additionally, Peters and co-workers observed a low BDFE_N–H_ of 39 kcal/mol with a cobaltocene complex in which the oxidation site (Co) and deprotonation site (N) are separated by > 7 Å (Figure 1A).^8^ Separate works by Chirik et al. and Abbenseth et al. described the weakening of the N–H bonds in PhNH_2_ to 60–70 kcal/mol when bound to V or Ta centers ligated by redox-active ligands that serve as electron-transfer sources.^4,5^ Thus, we sought to explore the impact of a multisite concerted proton electron transfer (MS-CPET) on lowering BDFE_N–H_ values using a system in which the amine substrate of interest is bound to one metal while a second metal serves as electron reservoir.

**Figure 1:**
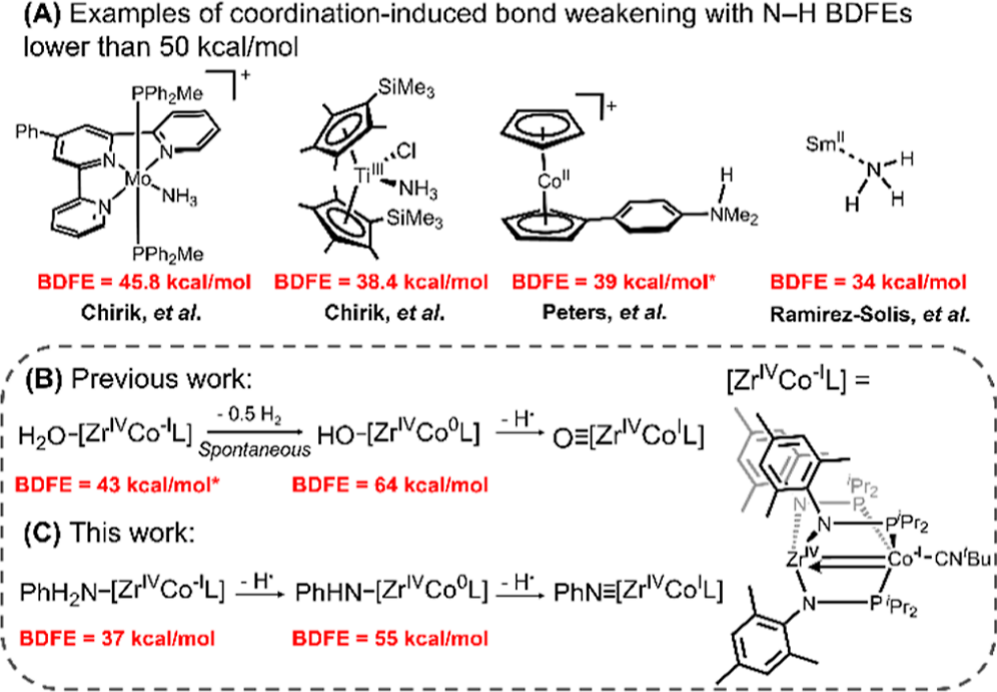
(A) Reported examples of BDFE_N–H_ values decreased below 50 kcal/mol through coordination-induced bond weakening.^2,6–8^ (B) Previously reported BDFE_O–H_ values through a heterobimetallic MS-CPET process.^12^ (C) BDFE_N–H_ values reported in this work using a heterobimetallic MS-CPET approach. BDFE values with * correspond to DFT calculated values. Portions of this graphic were adapted from ref 12. Available under a CC-BY 3.0 license. Copyright 2025 Feresin, Barden, Reyes, Ahyhankar, Barrett, and Thomas.

During the course of our previous studies, we found that tris­(phosphinoamide)-supported Zr^IV^/Co^–I^ complexes react with H_2_O to spontaneously release H_2_ to afford Zr^IV^/Co^0^ hydroxide compounds, implying a very weak BDFE_O–H_.^9–11^ The BDFE_O–H_ of HO–Zr­(MesNP^*i*^Pr_2_)_3_CoCN^*t*^Bu was determined experimentally to be 64 kcal/mol, demonstrating the viability of a multimetallic system to enable element–hydrogen bond cleavage through MS-CPET (Figure 1B).^12^ By employing an appended redox-active metal center (Co), a significant degree of coordination-induced bond weakening, driven by the low Co^I/0^ redox potential, was still observed even though the substrate was directly bound to a redox-inactive d^0^ Zr^IV^ site. Herein, we extend this heterobimetallic approach to N–H bonds to establish the generality of multimetallic coordination-induced bond weakening. Seeking to probe the impact of separated proton and electron transfer sites on the weakening of the N–H bonds of a coordinated substrate by experimentally determining BDFE_N–H_ values, we chose to begin our studies with a primary amine derivative for both operational simplicity and to prevent complications from potential dimerization processes (Figure 1C). Aniline (PhNH_2_) was specifically chosen for this study due to the increased crystallinity imparted by the aryl substituent, facilitating product isolation.

## Results & Discussion

### Synthesis and Characterization of **2–4**

Entry into this study began with the synthesis of a series of PhH_*x*_N–Zr­(MesNP^*i*^Pr_2_)_3_CoCN^*t*^Bu (*x* = 0–2) compounds that vary in cobalt oxidation state and the number of N-bound protons. One equiv PhNH_2_ was found to bind to the Zr site in (THF)­Zr^IV^(MesNP^*i*^Pr_2_)_3_Co^–I^CN^*t*^Bu (**1**) to generate PhH_2_N–Zr^IV^(MesNP^*i*^Pr_2_)_3_Co^–I^CN^*t*^Bu (**2**, Scheme 1). Complex **2** is diamagnetic and its ^1^H NMR spectrum features 11 peaks, consistent with a *C*_3_-symmetric molecule with free rotation about the Zr–N bond in solution on the NMR time scale. The two nitrogen-bound protons of the bound PhNH_2_ ligand appear as a singlet at 2.66 ppm integrating to two protons (Supporting Information Section 1.3.). Much like its precursor **1** and other related Zr^IV^/Co^–I^ bimetallic species,^10,13,14^ the ^31^P­{^1^H} NMR spectrum of **2** contains a very broad resonance that spans ∼ 30 ppm, centered at −56 ppm. Two N–H stretches corresponding to asymmetric and symmetric stretching vibrations were observed at 3337 cm^–1^ and 3265 cm^–1^ in the IR spectrum of **2**, along with a ν_C≡N_ stretch at 1974 cm^–1^.

**Scheme 1:**
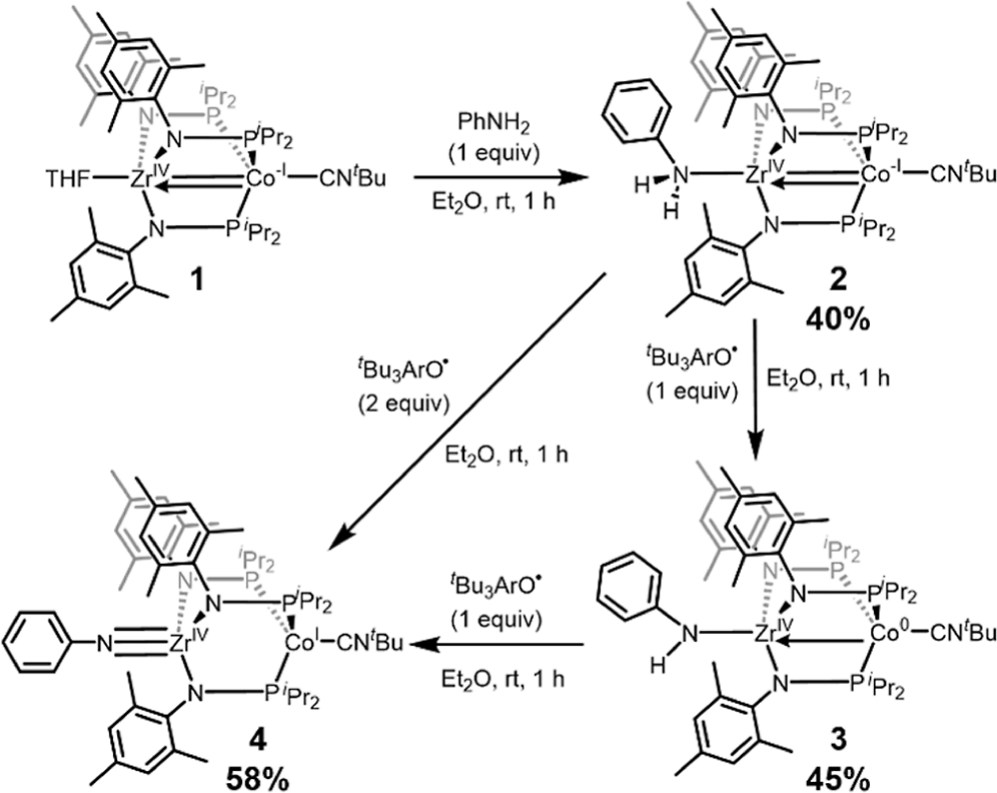
Synthetic Route to Generate 2, Followed by H-Atom Abstraction Reactions to Form 3 and 4

We next sought to sequentially abstract the H atoms from **2** to afford amido and imido species. Addition of 1 equiv of the 2,4,6-tris-*tert*-butylphenoxyl radical (^*t*^Bu_3_ArO^•^) to **2** resulted in the abstraction of a single H atom to produce PhHN-Zr^IV^(MesNP^*i*^Pr_2_)_3_Co^0^CN^*t*^Bu (**3**) via homolytic N–H bond cleavage (Scheme 1). The use of ^*t*^Bu_3_ArO^•^ was of paramount importance as reactions of **2** with (2,2,6,6-tetramethylpiperidin-1-yl)­oxyl (TEMPO) were complicated by the production of the previously reported oxo complex O≡Zr^IV^(MesNP^*i*^Pr_2_)_3_Co^I^CN^*t*^Bu via O atom transfer,^15^ as has been observed previously with a Ta–NH_2_Ph complex.^5^

Complex **3** is paramagnetic and its ^1^H NMR spectrum displays a total of 9 broad and paramagnetically shifted peaks, consistent with maintenance of *C*_3_ symmetry (Supporting Information Section 1.4.). As a result of their distance from the unpaired electrons on the Co atom, two of the peaks corresponding to the anilide moiety appear in the typical aromatic region of the spectrum (6.85, 6.67 ppm) and have discernible coupling. The magnetic moment of **3** was found to be 1.7 μ_B_ using Evans’ method, consistent with the expected *S* = 1/2 spin state. The ν_C≡N_ stretch observed in the IR spectrum of **3** also serves as an indicator of the one-electron oxidation of the Co center: The ν_C≡N_ of **3** is 2016 cm^–1^, which is 42 cm^–1^ higher than that of **2**, as would be expected for a compound with diminished π-backbonding into the C≡N π* orbital upon oxidation from Co^–I^ to Co^0^. In contrast to **2**, an N–H stretch was not observed in the IR spectrum of **3**, either because it was too weak to be distinguished from the baseline or because it had shifted to lower frequency where it was obscured by overlap with the C–H stretches.

Lastly, the imido species PhN≡Zr^IV^(MesNP^*i*^Pr_2_)_3_Co^I^CN^*t*^Bu (**4**) was synthesized via addition of 2 equiv ^*t*^Bu_3_ArO^•^ to **2** (Scheme 1). ^1^H NMR spectroscopy of **4** revealed 9 broad and paramagnetically shifted resonances and the spectrum is very similar to that of the previously reported mesitylimido derivative MesN≡Zr^IV^(MesNP^*i*^Pr_2_)_3_Co^I^CN^*t*^Bu (Supporting Information Section 1.5.).^13^ Evans’ method measurements on **4** resulted in an average μ_eff_ value of 2.8 μ_B_, consistent with the *S* = 1 spin state expected for a tetrahedral Co^I^ complex. The IR spectrum of **4** features a ν_C≡N_ of 2108 cm^–1^ that is increased a further 92 cm^–1^ with respect to **3**, in line with weakened π-backbonding upon oxidation from Co^0^ to Co^I^.

Complexes **2**, **3**, and **4** were characterized via single crystal X-ray diffraction and the resulting solid-state structures are shown in Figure 2; selected bond metrics are provided in Table 1. The Zr–N–C angles of **2–4** become more linear with each sequential H atom abstraction. The Zr–N–C angle in **2** is consistent with an sp^3^ hybridized nitrogen atom with an angle of 123.8(1)°, while the corresponding angles in **3** and **4** are 150.5(2)° and 171.9(1)° and consistent with sp^2^ and sp hybridized N atoms, respectively. In addition, with each H atom abstraction the Zr–N distances associated with the amine/amide/imide moiety decrease as the Zr–Co distance elongates. The Zr–N bond length in **2** is the longest among the complexes (2.587(2) Å). Since the Co center in **2** is the most reduced (Co^–I^, d^10^) and the aniline ligand is a relatively weak σ-donor, Co→Zr donation is maximized in **2**, leading to the shortest Zr–Co distance in the series (2.3772(4) Å) that is indicative of a highly polarized Zr–Co multiple bond. Upon abstraction of an H atom, the Zr–N distance contracts in **3** to 2.150(2) Å, consistent with conversion to an

**Figure 2:**
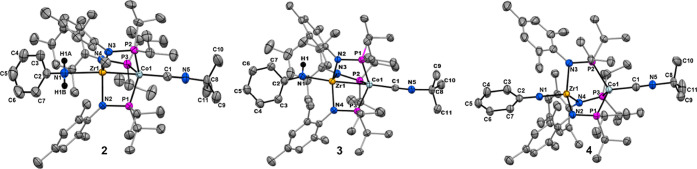
Solid-state structures of **2**, **3**, and **4** obtained using single crystal X-ray diffraction. Solvate molecules and hydrogen atoms, aside from the N-bound hydrogen atoms of **2** and **3** that were located in the difference map, have been omitted for clarity. Only one of two disordered positions of the ^*t*^Bu group in **3** and **4** is shown.

**Table 1:** Selected Bond Distances and Angles of **2-7** Obtained from Single Crystal X-ray Diffraction Data^a^

compound	Zr–N–C (°)	Zr–Co (Å)	Zr–N (Å)
**2**	123.8(1)	2.3772(4)	2.587(2)
**3**	150.5(2)	2.6369(7)	2.150(2)
**4**	171.9(1)	2.9818(7)	1.877(2)
**5**	142.3(5)	2.479(3)	2.287(6)
**6**	161.9(7)	2.7964(7)	1.970(9)
**7**	151.5(3)	2.7904(8)	2.041(3)

a N corresponds to the aniline-derived Zr-bound nitrogen atom.

LX-type amide ligand than can π-donate to the Lewis acidic Zr center. The resulting increased electron density on Zr combined with decreased electron density on Co upon oxidation to Co^0^ results in lengthening of the Co–Zr distance to 2.6369(7) Å, which corresponds to much a weaker Co→Zr dative σ bond. Lastly, the shortest Zr–N bond distance (1.877(2) Å) is observed for Zr^IV^/Co^I^ imido complex **4** owing to N→Zr π-donation and the resulting Zr≡N triple bond. As has been observed with previous Zr^IV^/Co^I^ species featuring Zr-ligand multiple bonds,^13,15^ the Zr–Co distance in **4** (2.9818(7) Å) indicates that metal–metal bonding is no longer present.

### BDFE Analysis via Electrochemical Experiments

Following the synthesis and characterization of the aforementioned heterobimetallic complexes **2**–**4**, open circuit potential (*E*°_OCP_) measurements were performed to determine the BDFE_N–H_ values of the aniline and anilide ligands in **2** and **3**. Following published procedures,^12,16–20^ the *E*°_OCP_(**2/3**) and *E*°_OCP_(**3*/*4**) were determined by measuring the *E*°_OCP_ (V vs H_2_) of five different ratios of **2**:**3** and **3**:**4** and plotting them vs the log­([**2**]/[**3**]) and log­([**3**]/[**4**]), respectively (Figure 3); the y-intercept of the resulting plot represents the *E*°_OCP_ of a 1:1 ratio of the two species that differ by an H atom. All the *E*°_OCP_ experiments were conducted in triplicate (Supporting Information Sections 2.1. and 2.2). The average y-intercept for the *E*°_OCP_ (**2/3**) was found to be −0.65 ± 0.018 V vs H_2_ while the average y-intercept for the *E*°_OCP_ (**3/4**) was determined as 0.14 ± 0.011 V vs H_2_. Figure 3 shows an example of one of the three trials performed for both the *E*°_OCP_(**2/3**) and *E*°_OCP_(**3/4**). The slopes obtained in the trials are in reasonable agreement with ideal Nernstian behavior for a one-electron process in a low dielectric constant nonaqueous solvent (THF), in which the *E*°_OCP_ decreases/increases by 0.0592 V for each order of magnitude change in ratio between the starting material and H atom abstracted product.

**Figure 3:**
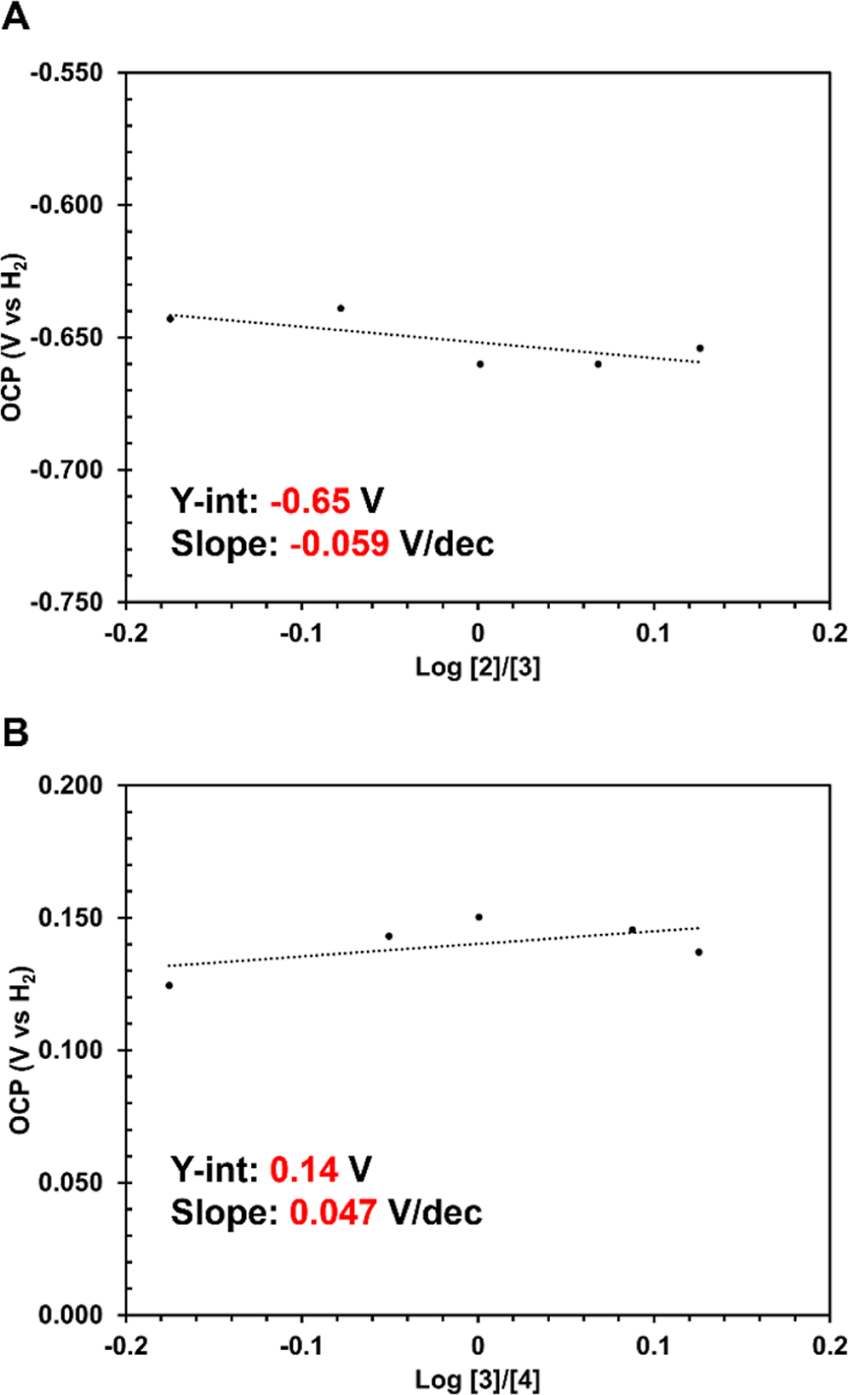
(A) Plot of *E*°_OCP_ (V vs H_2_) vs the log of the ratio of concentrations of **2** and **3** for one of three trials (THF, 100 mM [^*n*^Bu_4_N]­[PF_6_], 50 mM lutidine (lut), 50 mM [Hlut]­[BPh_4_]). (B) Plot of *E*°_OCP_ (V vs H_2_) vs the log of the ratio of concentrations of **3** and **4** for one of three trials (THF, 100 mM [^*n*^Bu_4_N]­[PF_6_], 50 mM NEt_3_, 50 mM [HNEt_3_]­[BPh_4_]).

With the *E*°_OCP_(**2/3**) and *E*°_OCP_(**3/4**) values in hand, eq 1 was used to calculate the BDFE_N–H_ values of complexes **2** and **3** as 37 ± 1 and 55 ± 1 kcal/mol, respectively (Table 2), using the known Δ*G*°(1/2*H*_2_(*g*)/*H*°̇̇_1M_) value of 52.0 kcal/mol in THF^16^ (see Supporting Information Section 3.4 for discussion of error analysis). The two BDFE_N–H_ values show significant coordination-induced bond weakening compared to free PhNH_2_ (86 kcal/mol)^21^ and PhNH^–^ (80 kcal/mol).^21^

1 $${BDFE}_{N-H}=23.06{\;E}_{OCP}^{{}^{\circ}}\left(N/NH\;vs\;H_{2}\right)+\Delta G^{{}^{\circ}}\left(\frac{1}{2}H_{2}\left(g\right)/H_{1M}^{\cdot }\right)$$

**Table 2:** Summary of BDFE Values (kcal/mol) Obtained for **2**, **3**, and HO–Zr­(MesNP^*i*^Pr_2_)_3_CoCN^*t*^Bu through OCP Measurements and DFT Calculations

compound	BDFE (OCP)	BDFE (DFT)
**2**	37 ± 1	37
**3**	55 ± 1	59
HO–Zr(MesNP^*i*^Pr_2_)_3_CoCN^*t*^Bu	64 ± 1^12^	60^12^

Density functional theory (DFT) calculations were performed to provide additional support for the experimentally determined BDFE values for **2** and **3** (Table 2). The BDFE value for **2** was calculated via DFT to be 37 kcal/mol, in excellent agreement with the experimentally determined value of 37 kcal/mol. The BDFE value for **3** was calculated to be 59 kcal/mol, overestimating the experimental value of 55 kcal/mol. Despite being higher than the value determined by *E*°_OCP_ experiments, this value falls within the range expected from BDFE test reactions (56.3< BDFE < 67.2, vide infra) and falls within previously observed DFT error of ± 4 kcal/mol.^12^

To provide further support for the BDFE_N–H_ values of **2** and **3** determined via the *E*°_OCP_ measurements, the stoichiometric reactivity of **2** and **3** toward H atom abstracting reagents was investigated. The BDFE_N–H_ of **2** is very low and limits the availability of H atom abstracting reagents with lower BDFEs to provide a lower bound for a BDFE range; however, when **2** was treated with 1,8-dichloroanthraquinone (BDFE_O–H_ = 56.3 kcal/mol)^21^ generation of **3** was observed (Supporting Information Section 4.1.). The observed reactivity demonstrates that the BDFE_N–H_ of **2** must be lower than 56 kcal/mol, which agrees with the BDFE_N–H_ of 37 ± 1 determined via *E*°_OCP_ measurements. Complex **3** was treated with *p*-benzoquinone (BDFE_O–H_ = 67.2 kcal/mol)^21^ and generation of **4** was observed, confirming that the BDFE_N–H_ of **3** must be less than 67 kcal/mol (Scheme 2). Treatment of **3** with 1,8-dichloroanthraquinone (BDFE_O–H_ = 56.3 kcal/mol)^21^ resulted in no conversion to **4**, although moderate broadening of the ^1^H NMR signals associated with **3** was noted (Supporting Information Section 4.2.). We suggest that this is the result of the BDFE values of **3** and 1,8-dichloroanthraquinone being within error of one another, allowing the reaction to reach equilibrium. However, significant conversion to **4** was not observed upon the addition of further equivalents of 1,8-dichloroanthraquinone to **3**.

**Scheme 2:**
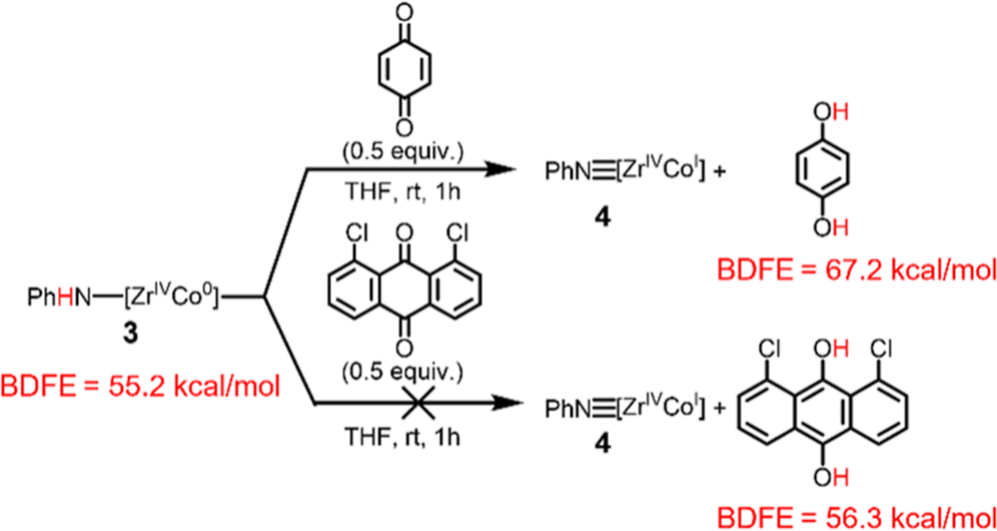
Reactivity of Complex 3 towards H Atom Abstraction Reagents

To gather more information about the contributions of redox potential to the remarkably low BDFE_N–H_ values of **2** and **3**, cyclic voltammetry (CV) measurements were performed for complexes **2–4** (Figure 4A). The cyclic voltammogram of complex **2** showed a reversible oxidation at −1.34 V (vs ferrocene/ferrocenium, Fc^+/0^) assigned to the Co^–I/0^ redox couple. The cyclic voltammogram of **2** also contained prominent features for a reversible reduction (*E*_1/2_ = −1.89 V) and a reversible oxidation (*E*_1/2_ = −0.92 V). The presence of these two features was initially puzzling since the Co center in **2** is already in its most reduced form (Co^–I^). This led us to hypothesize that the reduction event originates from a different Zr/Co species either present in the analyte solution or generated under electrochemical conditions. We previously observed interconversion between Co/Zr-oxo and Co/Zr-hydroxo complexes under oxidizing conditions as a result of spontaneous H atom transfer,^9^ lending credence to the latter possibility. To probe this hypothesis, cyclic voltammetry was performed with different initial potentials and scan directions, revealing that the two reversible features observed in the cyclic voltammogram of **2** are more prominent after scanning to positive potentials, implying that these features correspond to a new Zr/Co species generated from a chemical step that follows oxidation of **2** (Figure 4B).

**Figure 4:**
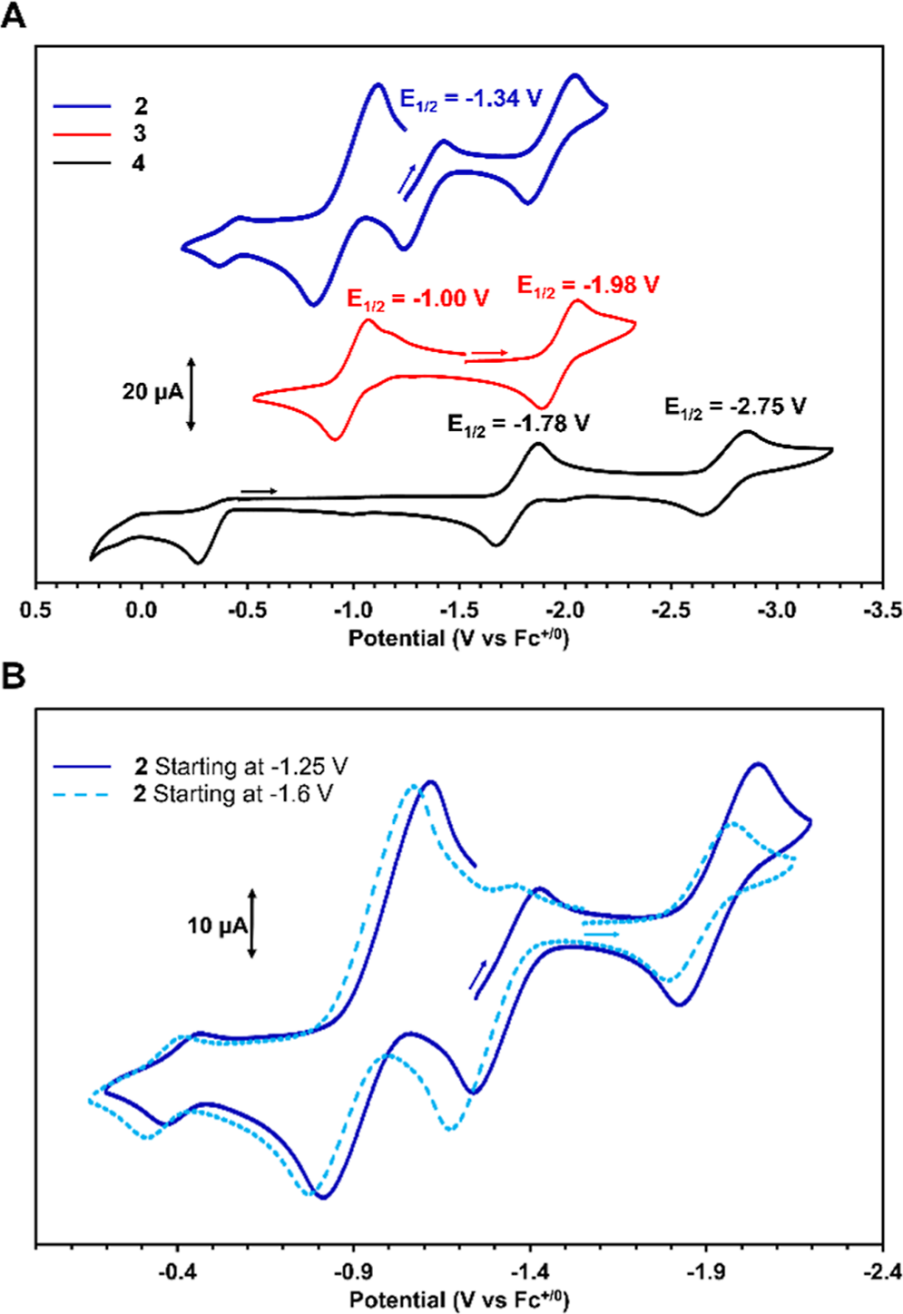
(A) Cyclic voltammograms of complexes **2–4** in a 0.1 M [^*n*^Bu_4_N]­[PF_6_] THF solution, (top, blue, **2**), (middle, red, **3**), (bottom, black, **4**). (B) Stacked cyclic voltammograms of complex **2** in a 0.1 M [^*n*^Bu_4_N]­[PF_6_] THF solution (blue, solid, **2**, starting at −1.25), (light blue, dashed, **2**, starting at −1.6 V).

In its cyclic voltammogram, complex **3** displays a reversible reduction at −1.98 V assigned to a Co^0/–I^ process and a reversible oxidation at −1.00 V assigned to oxidation from Co^0^ to Co^I^ (Figure 4A). These two redox events are nearly identical to the additional features observed in the voltammogram of **2**, suggesting that the latter features correspond to a Zr/Co amido species. Upon oxidation of complex **2**, we propose that the resulting cationic complex [PhH_2_N–Zr^IV^(MesNP^*i*^Pr_2_)_3_Co^–I^CN^*t*^Bu]^+^ spontaneously releases H^•^ (as 1/2 H_2_) to produce [PhHN-Zr­(MesNP^*i*^Pr_2_)_3_CoCN^*t*^Bu]^+^ (**7**, vide infra) whose cyclic voltammogram would be identical to that of **3**.

The cyclic voltammogram of **4** exhibits two reversible reductions at −1.78 V and −2.75 V, assigned to Co^I/0^ and Co^0/–I^ redox couples, respectively (Figure 4A). The Co^I/0^ potential of **4** is slightly more negative than the Co^I/0^ potential of the analogous Co/Zr-oxo complex O≡Zr­(MesNP^*i*^Pr_2_)_3_CoCN^*t*^Bu (−1.71 V),^9^ as would be expected for a relatively mild influence of the more basic and electron-donating nature of the imido ligand on the appended Co center in **4**. In contrast to **4**, a second reversible Co^0/–I^ reduction was not observed in the CV of the Co/Zr-oxo complex.

### Estimating p*K*_a_ Values

Compounds **2**, **3**, and **4** are interrelated by two corner-linked square schemes that relate the three compounds via sequential electron transfer and proton transfer events, or by hydrogen atom transfer along the diagonal (Scheme 3). Compound **3** is shared between both squares. Using the BDFE_N–H_ values determined via open circuit potential measurements for **2** and **3**, and the redox potentials determined from CV of complexes **2**–**4**, the relationship between these values established by Bordwell (eq 2) can provide estimated p*K*_a_ values for the N–H bonds in complexes **2** and **3**. The p*K*_a_ of **2** was estimated to be 16.6, while the p*K*_a_ of **3** was estimated to be 26.4. The limitation to these estimates is that the *E*°_OCP_ and CV measurements were performed in buffered electrolyte solution while the *C*_g,THF_ value of 59.9 kcal/mol corresponds to pure THF.^21^

2 $$BDFE\left(N-H\right)=23.06{\;E}^{{}^{\circ}}\left(X^{0/-}\right)+1.37\;pK_{a}\left(N-H\right)+C_{g,sol}$$

**Scheme 3:**
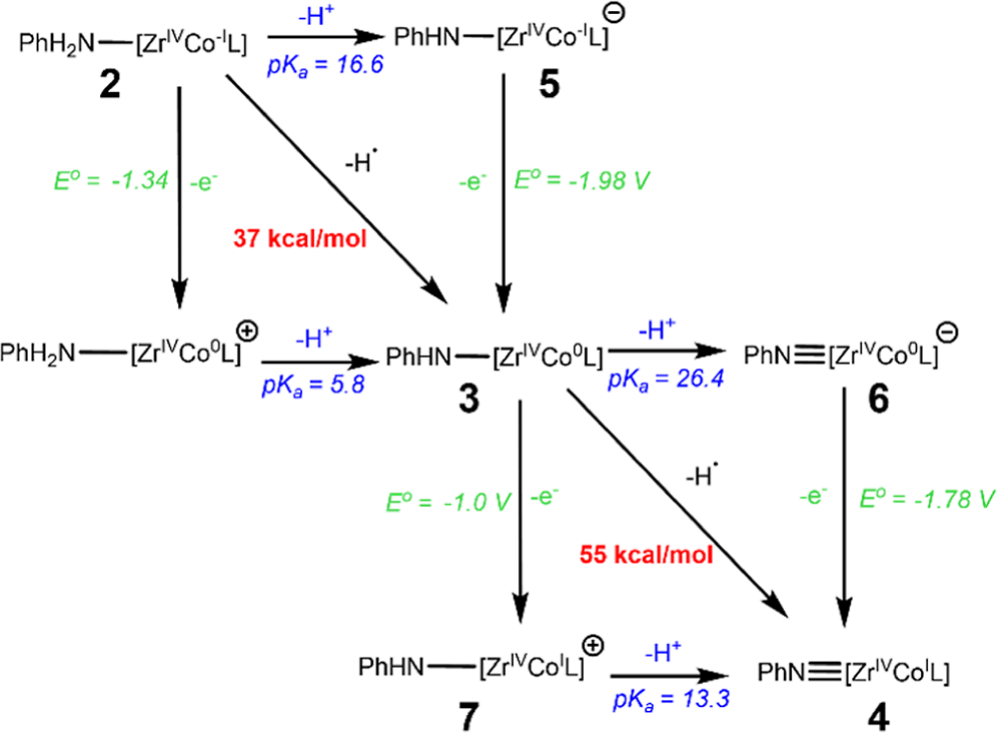
Square Scheme Showing the Stepwise and Concerted Interconversion of **2–4** ^a^

a The BDFE_N–H_ values were experimentally determined using *E*°_OCP_ measurements and the p*K*_a_ values are estimated from the BDFE_N–H_ and the *E*° values using eq 2.

A series of protonation/deprotonation reactions were performed in an attempt to generate the remaining, off-diagonal compounds in the double-square scheme in Scheme 3 and to provide some indirect validation of the determined BDFE_N–H_ values. The one-electron oxidized aniline adduct, [PhH_2_N–Zr­(MesNP^*i*^Pr_2_)_3_CoCN^*t*^Bu]^+^, was deemed too reactive to target owing to the aforementioned electrochemical behavior of **2**. When **2** was treated with Li­[N­(SiMe_3_)_2_] a deprotonation reaction occured to generate the lithium salt of [PhHN-Zr­(MesNP^*i*^Pr_2_)_3_CoCN^*t*^Bu]^−^ (**5**, Scheme 4). The sodium salt of compound **5** can be synthesized independently in 93% yield via the reduction of **3** with Na/Hg (Supporting Information, Section 1.6). Complex **5** is diamagnetic and, although it cannot be isolated owing to its instability toward oxidation by adventitious impurities, single crystals of **5** suitable for X-ray diffraction could be obtained. The solid-state structure of **5** (Scheme 4) reveals that, upon deprotonation, the Zr–N–C angle increases from 123.8(1)° in **2** to 142.3(5)° in **5** and the Zr–N distance decreases from 2.587(2) Å in **2** to 2.287(6) Å in **5**, consistent with sp^2^ hybridization of the N atom and π-donation to Zr in **5** (Table 1). The resulting increase in electron density on Zr in **5** compared to **2** limits Co→Zr donation, resulting in an ∼ 0.1 Å increase in the Zr–Co distance.

**Scheme 4:**
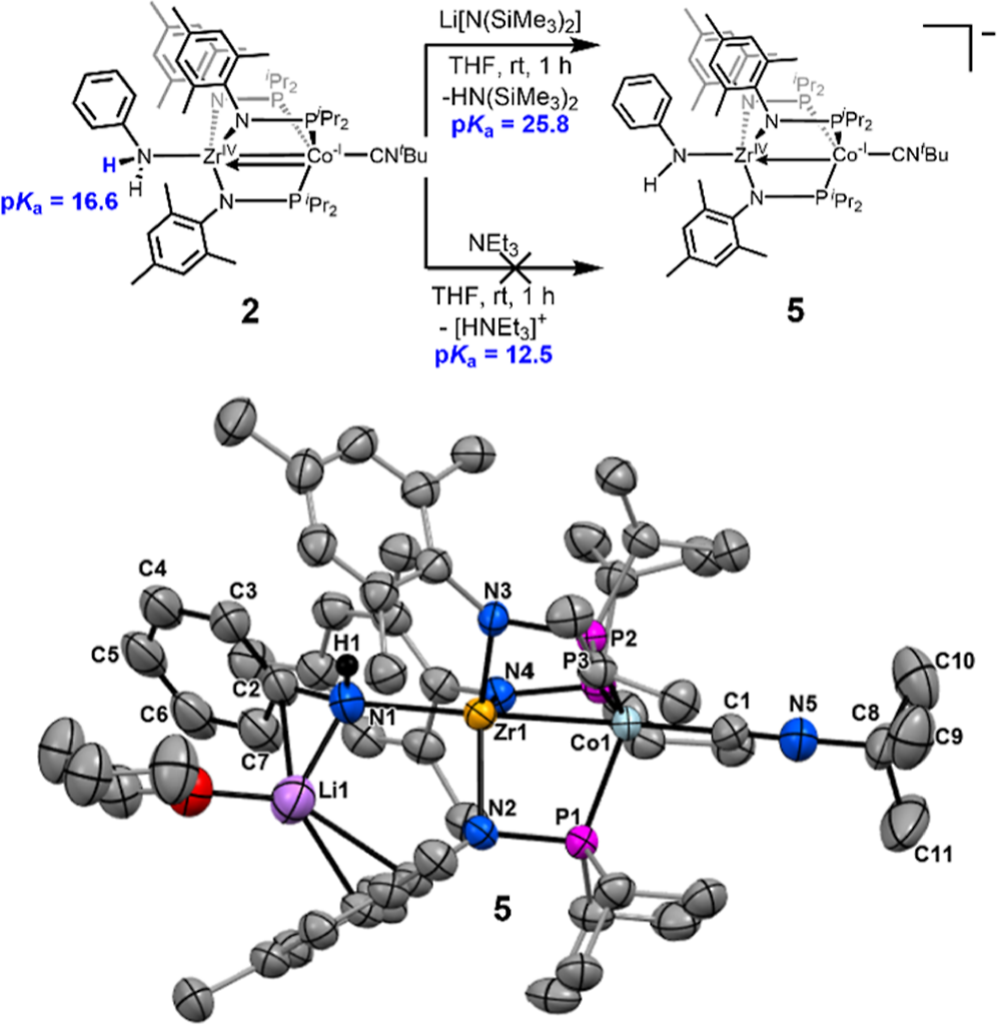
(Top) Reactions of 2 with Different Bases to Estimate the Upper and Lower Bounds of the p*K*_a_ of Complex **2**; (Bottom) Solid-state structure of 5 obtained using single crystal X-ray diffraction^a^

a Hydrogen atoms, except for the N–H hydrogen that was located in the difference map, have been omitted for clarity.

Compound **3**, whose p*K*_a_ was estimated to be 26.4, could also be readily deprotonated by Li­[N­(SiMe_3_)_2_] to generate the lithium salt of [PhN≡Zr­(MesNP^*i*^Pr_2_)_3_CoCN^*t*^Bu]^−^ (**6**, Scheme 5). Alternatively, compound **6** can be generated more cleanly via deprotonation of **3** with ^*n*^BuLi, albeit in low yield (4%, Supporting Information, Section 1.7). Complex **6** remains *S* = 1/2 with a similar pattern of broad and paramagnetically shifted resonances in its ^1^H NMR spectrum compared to **3**; however, the chemical shifts of **6** are distinct and shifted up to 0.5 ppm from those of **3** (Figure S10). Structural characterization of **6** revealed a significant decrease in the Zr–N distance upon deprotonation from 2.150(2) Å in **3** to 1.970(9) Å in **6**, consistent with increased Zr–N multiple bond character; however, the expected Zr–N triple bond is disrupted by interaction of the Li^+^ counterion of **6** with the basic imido nitrogen atom, resulting in a longer Zr–N distance (1.970(9) Å vs 1.877(2) Å) and less linear Zr–N–C angle (161.9(7)° vs 171.9(1)°) compared to neutral imido complex **4**. Nonetheless, the enhanced π-donation from the imido nitrogen atom decreases the Lewis acidity of the Zr^IV^ center, leading to diminished Co→Zr donation and a Zr–Co distance >0.2 Å longer compared to **3**.

**Scheme 5:**
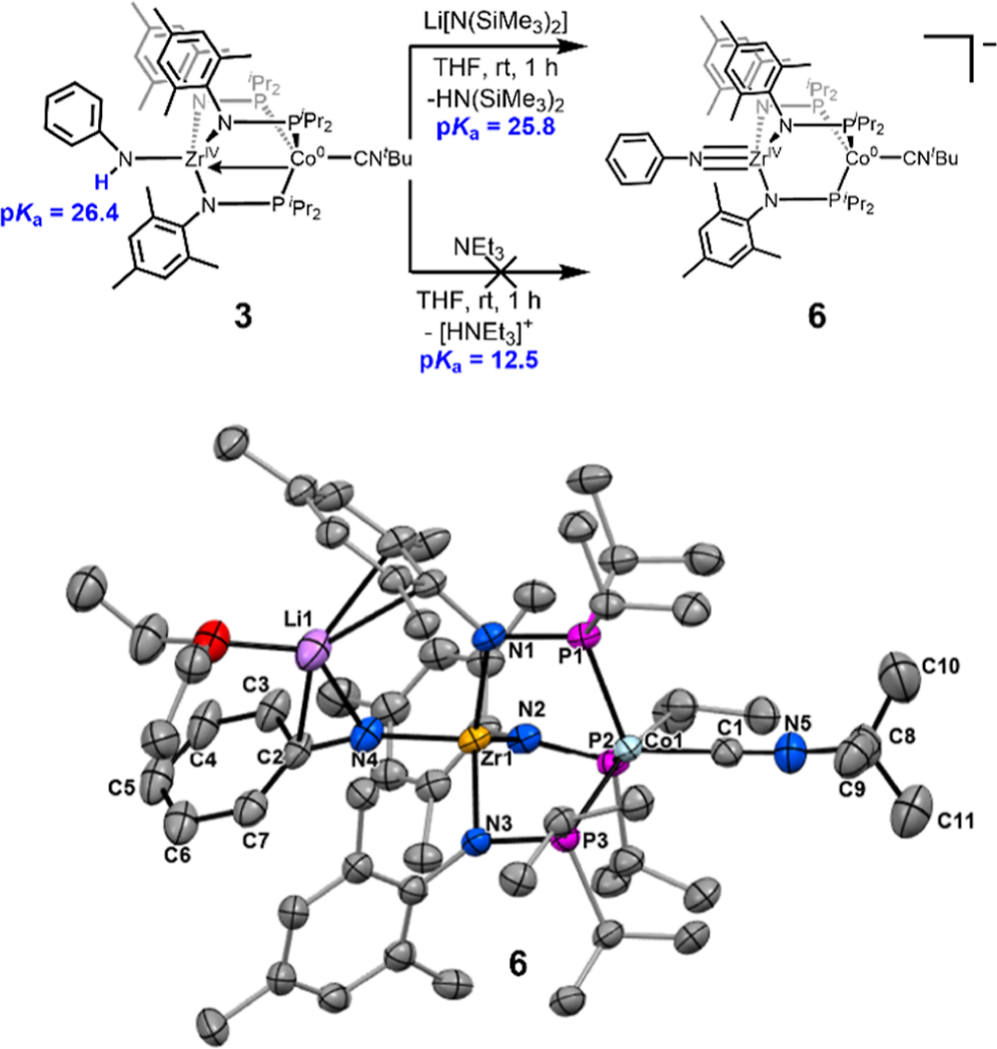
(Top) Reactions of 3 with Different Bases to Estimate the Upper and Lower Bounds of the p*K*_a_ of Complex 3; (Bottom) solid-state structure of **6** obtained using single crystal X-ray diffraction^a^

a Hydrogen atoms have been omitted for clarity.

The successful deprotonation of **2** and **3** with Li­[N­(SiMe_3_)_2_] indicates that the p*K*_a_ of both compounds is less than that of the conjugate acid of Li­[N­(SiMe_3_)_2_] (p*K*_a_ = 25.8).^22^ However, triethylamine was not found to deprotonate **2** or **3**, indicating that both compounds have a higher p*K*_a_ than HNEt_3_^+^ (p*K*_a_ = 12.5).^23^ Thus, stoichiometric deprotonation reactions provided a p*K*_a_ range for complexes **2** and **3** of 25.8 > p*K*_a_ > 12.5, which agrees with the estimated value of 16.6 for **2**. However, the p*K*_a_ range does not agree with the p*K*_a_ estimated for **3** using the measured BDFE_N–H_ of **3** and reduction potential of **4**. This disagreement may be due to the fact that p*K*_a_ values calculated using eq 2 represent the p*K*_a_ values in a buffered electrolyte solution while the reactions in Scheme 5 were performed in pure THF.

In a similar series of experiments, we examined protonation of imido complex **4** using acids with known p*K*_a_ values. Complex **4** was treated with [^*t*^BuHN=P­(pyrr)]­[BPh_4_] (pyrr = pyrrolidinyl, p*K*_a_ = 20.8)^23^ and no reaction occurred. In contrast, **4** was found to react with [HNEt_3_]­[BPh_4_] (p*K*_a_ = 12.5)^23^ to generate [PhHN-Zr­(MesNP^*i*^Pr_2_)_3_CoCN^*t*^Bu]­[BPh_4_] (**7**) in 64% yield (Scheme 6). Complex **7** is S = 1 (μ_eff_ = 3.1 μ_B_) and its ^1^H NMR spectrum features a similar pattern but distinct chemical shifts compared to **4**. The solid-state structure of **7** shown in Scheme 6 reveals that the Zr–N–C angled decreases upon protonation from 171.9 (1)° in **4** to 151.5(3)° in **7** with a concomitant elongation of the Zr–N distance from 1.877(2) Å to 2.041(3) Å (Table 1). Much like **4**, the Zr–Co distance (2.7904(8) Å) is too long to indicate direct Zr–Co bonding. The outcomes of these stoichiometric protonation test reactions indicate that the p*K*_a_ of **7** falls in the range of 20.8 > p*K*_a_ > 12.5, which agrees with the estimated value of 13.3.

**Scheme 6:**
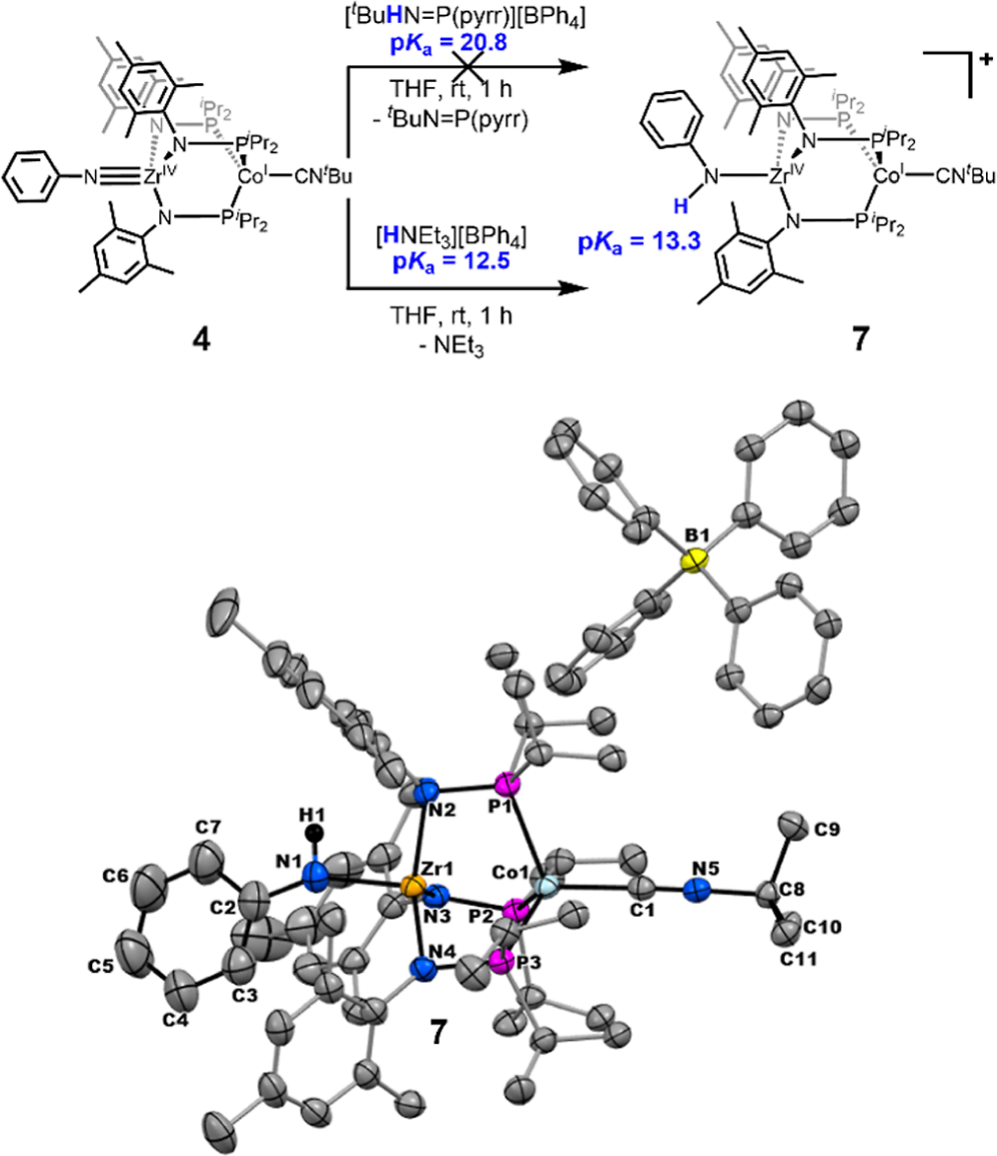
(Top) Reactions of 4 with Different Acids to Estimate the Upper and Lower Bounds of the pKa of Complex 7; (Bottom) Solid-state structure of 7 obtained using single crystal X-ray diffraction^a^

a Hydrogen atoms, except for the N–H hydrogen that was located in the difference map, have been omitted for clarity.

## Conclusions

In summary, we were able to successfully synthesize and characterize a series of Zr/Co aniline, amido, and imido species that can be interconverted by proton, electron, and hydrogen atom transfer steps to study fundamental aspects of N–H bond cleavage. The Zr^IV^/Co^–I^ aniline adduct **2** serves as a precursor to the Zr^IV^/Co^0^ amido complex **3** and Zr^IV^/Co^I^ imido complex **4**, generated via treatment of **2** with one or two equivalents of the 2,4,6-tris-*tert*-butylphenoxyl radical. With the complexes **2**, **3** and **4** in hand, *E*°_OCP_ measurements were performed in buffered electrolyte solution to determine the BDFE_N–H_ for the two consecutive H^•^ abstraction processes. Significant coordination-induced bond weakening was observed for the N–H bonds by pairing the electron-transfer capacity of the appended Co center with the redox-inactive (Zr^IV^, d^0^) substrate binding site in these molecules. The weakened N–H bonds in **2** join a small number of examples of BDFE_N–H_ values in the literature below 50 kcal/mol.^1,2,6,7^ The BDFE_N–H_ values of **2** and **3** are nearly 50 and 25 kcal/mol lower when compared to free PhNH_2_ (86 kcal/mol)^21^ and PhNH^–^ (80 kcal/mol), respectively.^21^ Using the measured BDFE_N–H_ values of **2** and **3** and the redox potentials of **2**–**4** obtained using CV, the p*K*_a_s of the aniline and amido complexes **2** and **3** were estimated to be 16.6 and 26.4, and these estimates were verified through deprotonation/protonation reactions to further support the obtained thermochemical data.

The BDFE_N–H_ value of the Zr-bound PhNH^–^ ligand in **3** was found to be 9 kcal/mol lower than the previously reported BDFE_O–H_ for the analogous hydroxo complex HO–Zr­(MesNP^*i*^Pr_2_)_3_CoCN^*t*^Bu (64 kcal/mol).^12^ In both of these examples the weakening of element-hydrogen bonds is driven by the low Co^I/0^ redox potentials, which are similar (within ∼100 mV) for analogous H_*x*_O–Zr/Co^0/I^ and PhH_*x*_N–Zr/Co^0/I^ species. The minor differences in redox potential mean that the lower BDFE_E–H_ found for **3** compared to the hydroxo analogue is attributed to differences in the p*K*_a_ of the element-hydrogen bonds. Indeed, the estimated p*K*_a_ values of the N–H bonds of **3** and **7** (26.4 and 13.3) are significantly lower than the p*K*_a_ values estimated for the O–H bonds of the corresponding neutral and cationic hydroxo compounds (31.5 and 21.7 kcal/mol, respectively).^12^ The difference in acidity is attributed to the electron-withdrawing phenyl group, which serves to stabilize the deprotonated dianionic imido ligand. It is therefore, anticipated that parent NH_3_/NH_2_^–^ derivatives will have higher p*K*_a_ and BDFE_N–H_ values, and this hypothesis will be explored in forthcoming studies.

This study establishes that the Zr/Co heterobimetallic system employs the redox-active Co site to drive significant coordination-induced bond weakening across different element-hydrogen bonds. By separating the sites of deprotonation and oxidation across two metal centers, the contributions of p*K*_a_ and redox potential to decrease substrate BDFE_E–H_ values can be varied separately, providing a means to fine-tune the degree of coordination-induced bond weakening involved in the oxidation of small molecule substrates.

## Experimental Section

### General Consideration

All experimental manipulations were carried out in a nitrogen-filled glovebox or via standard Schlenk techniques, unless otherwise noted. All glassware was oven-dried for a minimum of 2 h prior to use. All protio solvents were degassed with ultrahigh purity argon, dried using a Pure Process Technology solvent purification system, and stored over activated 3 Å molecular sieves. Benzene-*d*_6_ was degassed via repeated free-pump-thaw cycles using an oven-dried Schlenk flask and dried by storing over activated 3 Å molecular sieves. Aniline (PhNH_2_), lutidine (lut), and hexamethyldisilazane ((Me_3_Si)_2_NH) were dried over CaH_2_, distilled into an oven-dried vessel, and degassed by repeated freeze–pump–thaw cycles prior to bringing into the glovebox and stored over 3 Å molecular sieves. Lithium bis­(trimethylsiyl)­amide (Li­[N­(SiMe_3_)_2_]) was recrystallized from a saturated pentane or hexane solution stored at −35 °C and dried under vacuum at 70 °C prior to use. Tetrabutylammonium hexafluorophosphate ([^*n*^Bu_4_N]­[PF_6_]) was recrystallized twice from a saturated ethanol solution and dried under vacuum at 60 °C for 3 days before bringing into a glovebox. All other chemicals were purchased from commercial vendors and used without further purification. NMR spectra were recorded at ambient temperature on a Bruker Advance Neo 400 MHz (^11^B: 128 MHz, ^31^P: 162 MHz), a Bruker AVIII 600 MHz, or a Bruker AVANCE II HD Ascend 700 MHz NMR spectrometer. For ^1^H NMR and ^13^C NMR spectra, the solvent resonance was used as an internal standard. For ^11^B NMR spectra, boron trifluoride etherate (BF_3_^•^Et_2_O) was used as an external standard (0 ppm). For ^31^P NMR experiments, 85% phosphoric acid (H_3_PO_4_) was used as an external standard (0 ppm). Solid-state attenuated total reflection (ATR) infrared spectra (IR) were recorded using a Bruker Alpha II spectrometer controlled by OPUS software. Evans’ method was used for solution magnetic measurements by disregarding any diamagnetic contributions (Pascal’s constants were not used).^24,25^

### Synthetic Procedures

(THF)­Zr^IV^(MesNP^*i*^Pr_2_)_3_Co^–I^CN^*t*^Bu (**1**),^13^ ^*t*^Bu_3_ArO^•^,^26^ and [^*t*^BuHN=P­(pyrr)]­[BPh_4_][Bibr ref12] were synthesized following literature procedures. Triethylammonium tetraphenylborate ([HNEt_3_]­[BPh_4_]) and lutidinium tetraphenylborate ([Hlut]­[BPh_4_]) were synthesized via a modification of literature procedures^27,28^ by means of salt metathesis of [HNEt_3_]Cl and [Hlut]­Cl, respectively, with sodium tetraphenylborate in water for [HNEt_3_]­[BPh_4_] and in methanol for [Hlut]­[BPh_4_]. The solids were filtered, washed with water for [HNEt_3_]­[BPh_4_] and with ether for [Hlut]­[BPh_4_], and then dried in vacuo at 150 and 70 °C, respectively, for 3 days prior to bringing them into a glovebox.

Caution! extreme care should be taken both in the handling of the cryogen liquid nitrogen and its use in the Schlenk line or glovebox trap to avoid the condensation of oxygen from air.

Caution! *tert*-butylisocyanide is highly toxic and poses an inhalation hazard. It should be handled exclusively in a fume hood or glovebox.

#### Synthesis of PhH_2_N–Zr^IV^(MesNP^*i*^Pr_2_)_3_Co^–I^CN^*t*^Bu **(2)**

PhNH_2_ (57 μL mg, 0.62 mmol) was measured with a glass syringe and delivered directly to a dissolved solution of **1** (626.2 mg, 0.5927 mmol) in Et_2_O (5 mL) inside a scintillation vial containing a stirbar. Upon addition of PhNH_2_, the reaction mixture remained a red-brown color. After 1 h of stirring, the volatile components were removed in vacuo. The remaining red/brown solid was washed with Et_2_O (3 × 2 mL) and stored at −30 °C each time prior to decanting the Et_2_O to give analytically pure **2**. Yield: 255.5 mg, 40%. Pentane was diffused into a concentrated solution of **2** in Et_2_O at −30 °C to afford a red crystalline product, whose crystals were suitable for X-ray diffraction. ^1^H NMR (600 MHz, C_6_D_6_): δ 6.76–6.70 (m, 8H, Mes overlapping with *meta*-PhNH_2_), 6.60 (t, *J* = 7.4 Hz, 1H, *para*-PhNH_2_), 5.60 (d, *J* = 7.7 Hz, 2H, *ortho*-PhNH_2_), 2.91–2.85 (m, 6H, C*H*(CH_3_)_2_), 2.66 (s, 2H, N*H*_2_-PhNH_2_), 2.40 (s, 18H, Mes-Me), 2.16 (s, 9H, Mes-Me), 1.80–1.72 (m, 18H,CH­(C*H*_3_)_2_), 1.65–1.58 (m, 18H, CH­(C*H*_3_)_2_), 1.14 (s, 9H, C­(C*H*_3_)_3_). ^31^P­{^1^H} NMR (162 MHz, C_6_D_6_): δ very broad signal from 40 to 70 ppm. ^13^C­{^1^H} NMR­(176.0 MHz, C_6_D_6_): δ 149.30 (*ipso*-Mes), 143.01 (*C*NC­(CH_3_)_3_), 142.00 (*ipso*-PhNH_2_), 133.71 (Mes), 130.24 (Mes), 129.69 (Mes), 128.82 (*meta*-PhNH_2_), 121.53 (*para*-PhNH_2_), 118.13 (*ortho*-PhNH_2_), 53.91 (CN*C*(CH_3_)_3_, 45.07 (P*C*(CH_3_)_2_), 30.57 (CNC­(*C*H_3_)_3_), 23.65 (PC­(*C*H_3_)_2_), 22.97 (Mes­(*C*H_3_)_3_), 22.88 (PC­(*C*H_3_)_2_), 20.61 (Mes­(*C*H_3_)_3_). ATR IR: 3337 and 3265 cm^–1^ (ν_N–H_), 1974 cm^–1^ (ν_C≡N_). Owing to the reactivity of **2** with air, moisture, and protic solvents, satisfactory elemental analysis data could not be obtained.

#### Synthesis of PhHN-Zr^IV^(MesNP^*i*^Pr_2_)_3_Co^0^CN^*t*^Bu **(3)**

PhNH_2_ (41 μL, 0.45 mmol) was measured with a glass syringe and delivered directly to a dissolved solution of **1** (450.7 mg, 0.4266 mmol) in Et_2_O (5 mL) inside a scintillation vial containing a stirbar. Upon addition of PhNH_2_, the reaction mixture remained a red-brown color. After 5 min of stirring, ^*t*^Bu_3_ArO^•^ (105.3 mg, 0.4028 mmol) was dissolved in Et_2_O (5 mL) and then added to the reaction mixture; a color change to orange-yellow was observed. After 1 h of stirring, the volatile components were removed in vacuo. The remaining orange-yellow solid was washed with Et_2_O (3 × 2 mL) and stored at −35 °C each time prior to decanting the Et_2_O to give analytically pure **3** as a red crystalline product. Yield: 194.5 mg, 45%. A concentrated solution of **3** in Et_2_O stored at −30 °C afforded orange/yellow crystalline product. ^1^H NMR (600 MHz, C_6_D_6_): δ 7.53, 6.85 (t, *J* = 6.8 Hz), 6.67 (d, *J* = 8.2 Hz), 6.11, 5.37 (br), 4.60, 2.82, 2.08, −1.62 (br). Evans’ method (μ_eff_, C_6_D_6_): average value of 1.7(1) B.M. ATR IR: 2016 cm^–1^ (ν_C≡N_). Owing to the reactivity of **3** with air, moisture, and protic solvents, satisfactory elemental analysis data could not be obtained.

#### Synthesis of PhN≡Zr^IV^(MesNP^*i*^Pr_2_)_3_Co^I^CN^*t*^Bu **(4)**

PhNH_2_ (12 μL, 0.13 mmol) was measured with a glass syringe and delivered directly to a dissolved solution of **1** (134.7 mg, 0.1275 mmol) in Et_2_O (5 mL) inside a scintillation vial containing a stirbar. Upon addition of PhNH_2_, the reaction mixture remained a red-brown color. After 5 min of stirring, ^*t*^Bu_3_ArO^•^ (68.3 mg, 0.261 mmol) was dissolved in Et_2_O (1 mL) and then added to the reaction mixture; a green solid precipitated from the solution. After 1 h of stirring, the solution was decanted, and the green precipitate was washed with Et_2_O (3 × 1 mL). Any remaining volatile components were removed from the remaining solid in vacuo. Pentane was diffused into a concentrated solution of **4** in THF at room temperature to afford green crystalline product, whose crystals were suitable for X-ray diffraction. Yield: 79.8 mg, 58%. ^1^H NMR (600 MHz, C_6_D_6_): δ 18.85, 8.78, 8.34 (t, *J* = 6.8 Hz), 8.10, 7.13 (t, *J* = 6.8 Hz), 3.42, 2.25, −3.26 (br), −5.13 (br). Evans’ method (μ_eff_, CD_2_Cl_2_): Average value of 2.8(1) B.M. ATR IR: 2108 cm^–1^ (ν_C≡N_). Owing to the reactivity of **4** with air, moisture, and protic solvents, satisfactory elemental analysis data could not be obtained.

#### Synthesis of [PhHN-Zr^IV^(MesNP^*i*^Pr_2_)_3_Co^–I^CN^*t*^Bu]^−^ **(5)**

Method 1: complex **2** (41.0 mg, 0.0381 mmol) was dissolved in THF (5 mL) and the solution was stirred. Li­[N­(SiMe_3_)_2_] (6.8 mg, 0.041 mmol) was dissolved in THF (1 mL) and added to the stirring solution of **2**. The solution remained dark red. After 1 h of stirring, the volatile components were removed in vacuo. A concentrated solution of **5** in Et_2_O stored at −35 °C afforded red crystalline product, whose crystals were suitable for X-ray diffraction. Method 2: A 0.5% Na/Hg amalgam was prepared in THF (2 mL) from 2.7 mg Na (0.12 mmol) and Hg (0.5456 g) and stirred. A solution of **3** (0.1310 mg, 0.12 mmol) in THF (∼2 mL) was added to the stirring amalgam. After 1 h of stirring, the solution was decanted from the amalgam and filtered through Celite. The volatile components were removed from the filtrate in vacuo. The resulting solid was extracted into benzene (2 mL) and filtered through Celite. The volatile components were removed from the filtrate in vacuo, yielding **5** as a red product. Yield as isolated: 0.1321 mg, 92.6%. ^1^H NMR (600 MHz, C_6_D_6_): δ 6.72 (s, 6H, Mes), 6.42 (t, 2H, *meta*-PhNH), 6.16–6.14 (t, 1H, *para*-PhNH), 5.18 (d, *J* = 8.2 Hz, 2H, *ortho*-PhNH), 2.94 (m, 6H, C*H*(CH_3_)_2_), 2.64 (s, 18H, Mes-Me), 2.54 (s, 1H, N*H*-PhNH_2_), 2.11 (s, 9H, Mes-Me), 1.97–1.93 (m, 18H, CH­(C*H*_3_)_2_), 1.71 (m, 18H, CH­(C*H*_3_)_2_), 1.33 (s, 9H, C­(C*H*_3_)_3_). The ^31^P­{^1^H} NMR signal was too broad to observe. Owing to the instability of **5**, no further spectroscopic characterization data was obtained.

#### Synthesis of [PhN≡Zr^IV^(MesNP^*i*^Pr_2_)_3_Co^0^CN^*t*^Bu]^−^ **(6)**

Complex **3** (164.3 mg, 0.1526 mmol) was dissolved in Et_2_O (3 mL) and the solution was stirred. ^*n*^BuLi (100 μL, 1.6 M in hexanes, 0.16 mmol) was measured with a glass syringe and delivered to a scintillation vial containing Et_2_O (1 mL). The diluted ^*n*^BuLi solution was then added dropwise to the stirring solution of **3**. Upon addition of ^*n*^BuLi, the solution changed from a red color to a dark green color. After 5 min of stirring, the volatile components were removed in vacuo. Pentane was diffused into a concentrated solution of **6** in Et_2_O at −35 °C to afford yellow crystalline product, whose crystals were suitable for X-ray diffraction. Crystalline yield: 6.8 mg, 4.1%. ^1^H NMR (600 MHz, C_6_D_6_): δ 7.49, 7.34, 6.77, 6.39, 5.13, 3.27, 2.96, 2.16, −2.13. Owing to the instability of **6**, no further spectroscopic characterization data was obtained.

#### Synthesis of [PhHN-Zr^IV^(MesNP^*i*^Pr_2_)_3_Co^I^CN^*t*^Bu]­[BPh_4_] **(7)**

[HNEt_3_]­[BPh_4_] (5.2 mg, 0.012 mmol) was dissolved in THF (1 mL) and then added to a stirring solution of **4** (13.3 mg, 0.0124 mol) in THF (2 mL). Upon addition of the acid, the reaction mixture changed color from green to yellow. The reaction was allowed to stir for 1 h prior to removing the volatile components in vacuo. The remaining yellow solid was washed with cold (−35 °C) Et_2_O (3 × 1 mL) to give analytically pure **7**. Yield: 11.4 mg, 64%. Pentane was diffused into a concentrated solution of **7** in PhF at −35 °C to afford yellow crystalline product, whose crystals were suitable for X-ray diffraction. ^1^H NMR (400 MHz, C_6_D_6_): δ 23.03 (br), 11.49 (br), 9.42 (br), 8.19 (s, 2H), 7.41 (d, *J* = 15.4 Hz, *para*-BPh_4_), 7.23 (br, *meta*-BPh_4_), 7.05 (br, *ortho*-BPh_4_), 2.70 (br), 2.12, −4.03 (br). ^11^B NMR (128 MHz, C_6_D_6_): δ −5.74. Evans’ method (μ_eff_, PhF): average value of 3.1(1) B.M. ATR IR: 2154 cm^–1^ (ν_C≡N_). Owing to the reactivity of **7** with air, moisture, and protic solvents, satisfactory elemental analysis data could not be obtained.

### X-ray Crystallography Procedures

Single-crystal X-ray diffraction studies were carried out on a Bruker Kappa Photon II CPAD diffractometer equipped with Mo K_α_ radiation (λ = 0.71073 Å) or Cu K_α_ radiation (λ = 1.54178 Å). Data were collected in a nitrogen gas stream at 100(2) K (Oxford Cryosystems Crysostream 700) using φ and ω scans. The data were integrated using the Bruker SAINT software program and scaled using the SADABS software program within the APEX4 GUI. Solution by dual-space method (SHELXT) produced a complete phasing model for refinement.^29^ Refinement was performed within the OLEX2 ^30^ GUI using SHELXL.^31^ All nonhydrogen atoms were refined anisotropically by full-matrix least-squares (SHELXL).^31^ All carbon-bonded hydrogen atoms were placed using a riding model. All other hydrogen atoms (N-bonded) were located in the difference map. Their positions were constrained relative to their parent atom using the appropriate FTIX command in SHELXL. Publication figures were generated with Mercury.^32^ Crystallographic details are summarized in Tables S1–S3 of the Supporting Information.

### Computational Details

All DFT calculations were carried out with the ORCA 6.0.1 program package^33,34^ in the gas phase, with all geometry optimizations and single point calculations performed using the ωb97x-D3 functional.^35^ For geometry optimizations, the basis set Def2-TZVPP was used on the zirconium and cobalt atoms, and the Def2-SVP basis set was used for the carbon, hydrogen, phosphorus, and nitrogen atoms.^36–38^ Single point calculations on optimized geometries were performed for all atoms with the Def2-TZVPP basis set. Local minima were confirmed by the absence of imaginary (negative) frequencies in analytical frequency calculations. Free energies of complexes were calculated by adjusting single point energies (calculated at the Def2-TZVPP level for all atoms) using the thermochemical factors obtained from frequency calculations (calculated at the Def2-SVP level for nonmetal atoms).

## Supplementary Material





## Notes

Detailed procedures and additional data for electrochemical measurements, spectroscopic data, computational details, X-ray crystallography details for **2**–**7** (PDF)
Cartesian coordinates for all DFT-optimized structures (XYZ)

## References

[1] Boekell N. G., Flowers R. A. (2022). Coordination-Induced Bond Weakening. Chem. Rev..

[2] Bezdek M. J., Guo S., Chirik P. J. (2016). Coordination-Induced
Weakening of Ammonia, Water, and
Hydrazine X–H Bonds in a Molybdenum Complex. Sci..

[3] Dunn P. L., Cook B. J., Johnson S. I., Appel A. M., Bullock R. M. (2020). Oxidation
of Ammonia with Molecular Complexes. J. Am.
Chem. Soc..

[4] Milsmann C., Semproni S. P., Chirik P. J. (2014). N–N Bond Cleavage of 1,2-Diarylhydrazines
and N–H Bond Formation via H-Atom Transfer in Vanadium Complexes
Supported by a Redox-Active Ligand. J. Am. Chem.
Soc..

[5] Pavlidis S., Alasadi J., Opis-Basilio A., Abbenseth J. (2025). Two-Fold Proton
Coupled Electron Transfer of a Ta­(v) Aniline Complex Mediated by a
Redox Active NNN Pincer Ligand. Dalton Trans..

[6] Pappas I., Chirik P. J. (2015). Ammonia Synthesis by Hydrogenolysis of Titanium–Nitrogen
Bonds Using Proton Coupled Electron Transfer. J. Am. Chem. Soc..

[7] Ramírez-Solís A., Boekell N. G., León-Pimentel C. I., Saint-Martin H., Bartulovich C. O., Flowers R. A. (2022). Ammonia Solvation vs Aqueous Solvation
of Samarium Diiodide. A Theoretical and Experimental Approach to Understanding
Bond Activation Upon Coordination to Sm­(II). J. Org. Chem..

[8] Chalkley M. J., Garrido-Barros P., Peters J. C. (2020). A Molecular Mediator for Reductive
Concerted Proton-Electron Transfers via Electrocatalysis. Science.

[9] Zhang H., Hatzis G. P., Dickie D. A., Moore C. E., Thomas C. M. (2020). Redox Chemistry
and H-Atom Abstraction Reactivity of a Terminal Zirconium­(IV) Oxo
Compound Mediated by an Appended Cobalt­(I) Center. Chem. Sci..

[10] Krogman J. P., Bezpalko M. W., Foxman B. M., Thomas C. M. (2013). Synthesis, Structure,
and Reactivity of an Anionic Zr–Oxo Relevant to CO_2_ Reduction by a Zr/Co Heterobimetallic Complex. Inorg. Chem..

[11] Napoline J.
W., Krogman J. P., Shi R., Kuppuswamy S., Bezpalko M. W., Foxman B. M., Thomas C. M. (2013). Activation
of E–H
and E–E (E = S, O) Bonds by Heterobimetallic Zr/Co Complexes:
Evidence for Both One- and Two-Electron Processes. Eur. J. Inorg. Chem..

[12] Feresin J., Barden B. A., Reyes J. A., Abhyankar P. C., Barrett S. M., Thomas C. M. (2025). Heterobimetallic Multi-Site Concerted
Proton Electron Transfer (MS-CPET) Promotes Coordination-Induced O–H
Bond Weakening. Chem. Sci..

[13] Krogman J. P., Bezpalko M. W., Foxman B. M., Thomas C. M. (2016). Multi-Electron Redox
Processes at a Zr­(IV) Center Facilitated by an Appended Redox-Active
Cobalt-Containing Metalloligand. Dalton Trans..

[14] Greenwood B. P., Rowe G. T., Chen C.-H., Foxman B. M., Thomas C. M. (2010). Metal–Metal
Multiple Bonds in Early/Late Heterobimetallics Support Unusual Trigonal
Monopyramidal Geometries at Both Zr and Co. J. Am. Chem. Soc..

[15] Zhang H., Hatzis G. P., Moore C. E., Dickie D. A., Bezpalko M. W., Foxman B. M., Thomas C. M. (2019). O_2_ Activation by a Heterobimetallic
Zr/Co Complex. J. Am. Chem. Soc..

[16] Wise C. F., Agarwal R. G., Mayer J. M. (2020). Determining
Proton-Coupled Standard
Potentials and X–H Bond Dissociation Free Energies in Nonaqueous
Solvents Using Open-Circuit Potential Measurements. J. Am. Chem. Soc..

[17] Mondal S., Zhang W., Zhang S. (2024). Thermodynamics of Proton-Coupled
Electron Transfer at Tricopper μ-Oxo/Hydroxo/Aqua Complexes. J. Am. Chem. Soc..

[18] Wu T., Puri A., Qiu Y. L., Ye D., Sarma R., Wang Y., Kowalewski T., Siegler M. A., Swart M., Garcia-Bosch I. (2024). Tuning the Thermochemistry and Reactivity of a Series
of Cu-Based 4H^+^/4e^–^ Electron-Coupled-Proton
Buffers. Inorg. Chem..

[19] Smith A. M., Miller A. J. M. (2024). Open Circuit
Potential Method for Thermodynamic Hydricity
Measurements of Metal Hydrides. Organometallics.

[20] Groff B. D., Cattaneo M., Coste S. C., Pressley C. A., Mercado B. Q., Mayer J. M. (2023). Independent Tuning of the p*K*
_a_ or the *E*
_1/2_ in
a Family of Ruthenium
Pyridine–Imidazole Complexes. Inorg.
Chem..

[21] Agarwal R. G., Coste S. C., Groff B. D., Heuer A. M., Noh H., Parada G. A., Wise C. F., Nichols E. M., Warren J. J., Mayer J. M. (2022). Free Energies of Proton-Coupled Electron Transfer Reagents
and Their Applications. Chem. Rev..

[22] Fraser R. R., Mansour T. S., Savard S. (1985). Acidity Measurements
on Pyridines
in Tetrahydrofuran Using Lithiated Silylamines. J. Org. Chem..

[23] Kaljurand I., Rodima T., Pihl A., Mäemets V., Leito I., Koppel I. A., Mishima M. (2003). Acid–Base Equilibria
in Nonpolar Media. 4. Extension of the Self-Consistent Basicity Scale
in THF Medium. Gas-Phase Basicities of Phosphazenes. J. Org. Chem..

[24] Evans D. F. (1959). 400. The
Determination of the Paramagnetic Susceptibility of Substances in
Solution by Nuclear Magnetic Resonance. J. Chem.
Soc..

[25] Sur S. K. (1989). Measurement
of Magnetic Susceptibility and Magnetic Moment of Paramagnetic Molecules
in Solution by High-Field Fourier Transform NMR Spectroscopy. J. Magn. Reson..

[26] Manner V. W., Markle T. F., Freudenthal J. H., Roth J. P., Mayer J. M. (2008). The First
Crystal Structure of a Monomeric Phenoxyl Radical: 2,4,6-Tri-Tert-Butylphenoxyl
Radical. Chem. Commun..

[27] Grönberg K. L. C., Henderson R. A., Oglieve K. E. (1998). A Unified Mechanism for the Stoichiometric
Reduction of H^+^ and C_2_H_2_ by [Fe_4_S_4_(SPh)_4_]^3–^ in MeCN. J. Chem. Soc., Dalton Trans..

[28] Dilworth J. R., Henderson R. A., Dahlstrom P., Nicholson T., Zubieta J. A. (1987). The Chemistry of the Hydrazido(1−)-Ligand. Preparations
and Crystal Structures of [Mo­(NHNHCO_2_ Me)­(NNCO_2_Me)­(S _2_CNMe_2_)_2_], [Mo­(NHNMePh)­(NNMePh)­(S_2_CNMe_2_)_2_ ]­BPh_4_, and [ReCl_2_(NHNHCOPh)­(NNHCOPh)­(PPh_3_)_2_]. Mechanism
of Formation of Substituted Hydrazines from [Mo­(NNRPh)_2_(S_2_CNMe_2_)_2_ ]­(R = Me or Ph). J. Chem. Soc., Dalton Trans..

[29] Sheldrick G. M. (2015). *SHELXT* – Integrated Space-Group and Crystal-Structure
Determination. Acta Crystallogr., Sect. A:Found.
Adv..

[30] Dolomanov O. V., Bourhis L. J., Gildea R. J., Howard J. A. K., Puschmann H. (2009). *OLEX2*: A Complete
Structure Solution, Refinement and Analysis Program. J. Appl. Crystallogr..

[31] Sheldrick G. M. (2015). Crystal
Structure Refinement with *SHELXL*. Acta Crystallogr., Sect. C:Struct. Chem..

[32] Macrae C. F., Bruno I. J., Chisholm J. A., Edgington P. R., McCabe P., Pidcock E., Rodriguez-Monge L., Taylor R., Van De Streek J., Wood P. A. (2008). *Mercury
CSD 2.0* – New Features for the Visualization and Investigation
of Crystal Structures. J. Appl. Crystallogr..

[33] Neese F. (2012). The ORCA Program
System. WIREs Comput. Mol. Sci..

[34] Neese F. (2025). Software Update:
The ORCA Program System–Version 6.0. WIREs Comput. Mol. Sci..

[35] Chai J.-D., Head-Gordon M. (2008). Long-Range Corrected Hybrid Density Functionals with
Damped Atom–Atom Dispersion Corrections. Phys. Chem. Chem. Phys..

[36] Rassolov V. A., Pople J. A., Ratner M. A., Windus T. L. (1998). 6–31G* Basis
Set for Atoms K through Zn. J. Chem. Phys..

[37] Weigend F., Ahlrichs R. (2005). Balanced Basis Sets
of Split Valence, Triple Zeta Valence
and Quadruple Zeta Valence Quality for H to Rn: Design and Assessment
of Accuracy. Phys. Chem. Chem. Phys..

[38] Zheng J., Xu X., Truhlar D. G. (2011). Minimally Augmented Karlsruhe Basis Sets. Theor. Chem. Acc..

