# Supplementary material for: Coordination-Induced Weakening of N–H Bonds Driven by Bimetallic Cooperativity in Zr/Co Compounds
Source: Inorg Chem. 2026 Jan 27;65(5):3115–24. doi: 10.1021/acs.inorgchem.5c05652 (PMC12892316; doi:10.1021/acs.inorgchem.5c05652)
Supplement: Supplementary file 1 [file ic5c05652_si_001.pdf]

SUPPORTING INFORMATION

Coordination-Induced Weakening of N—H bonds Driven by Bimetallic Cooperativity in Zr/Co Compounds

Julia Feresin,[a] Megan. C. Ford,[a] Matthew K. Vascura,[b] Curtis E. Moore,[a] Seth M. Barrett,[b*] and Christine M. Thomas[a*]

[a]Department of Chemistry and Biochemistry, The Ohio State University, 100 W. 18th Ave, Columbus, OH 43210, USA

[b]Department of Chemistry, Muskingum University, 260 Stadium Drive, New Concord, OH 43762, USA

thomasc@chemistry.ohio-state.edu; sbarrett@muskingum.edu

TABLE OF CONTENTS

1. Synthesis and Characterization S3

1.1. Characterization of $(PhH_2N)Zr^{IV}(MesNP^iPr_2)_3Co^{-I}CN^tBu$ (2) S3

1.2. Characterization of $(PhHN)Zr^{IV}(MesNP^iPr_2)_3Co^{0}CN^tBu$ (3) S7

1.3. Characterization of $(PhN)Zr^{IV}(MesNP^iPr_2)_3Co^{I}CN^tBu$ (4) S9

1.4. Characterization of $[PhHNZr^{IV}(MesNP^iPr_2)_3Co^{-I}CN^tBu]^-$ (5) S11

1.5. Characterization of $[PhNZr^{IV}(MesNP^iPr_2)_3Co^{0}CN^tBu]^-$ (6) S12

1.6. Characterization of $[PhHNZr^{IV}(MesNP^iPr_2)_3Co^{I}CN^tBu][BPh_4]$ (7) S13

2. Cyclic Voltammetry & Open-Circuit Potential Measurements S16

2.1. $E°_{OCP}$ (NH_2/NH) vs H_2 .... S16

2.2. $E°_{OCP}$ (NH/N) vs H_2 .... S26

2.3. Cyclic voltammetry of $(PhH_2N)Zr^{IV}(MesNP^iPr_2)_3Co^{-I}CN^tBu$ (2) S39

2.4. Cyclic voltammetry of $(PhHN)Zr^{IV}(MesNP^iPr_2)_3Co^{0}CN^tBu$ (3) S40

2.5. Cyclic voltammetry of $(PhN)Zr^{IV}(MesNP^iPr_2)_3Co^{I}CN^tBu$ (4) S41

2.6. Cyclic voltammetry of $[PhHNZr^{IV}(MesNP^iPr_2)_3Co^{I}CN^tBu][BPh_4]$ (7) S42

3. Direct conversion of OCP (V vs H_2) to $BDFE_{N–H}$ S43

3.1. $BDFE_{N–H}$ sample calculation of first H atom abstraction between complexes $(PhH_2N)Zr^{IV}(MesNP^iPr_2)_3Co^{-I}CN^tBu$ (2) to $(PhHN)Zr^{IV}(MesNP^iPr_2)_3Co^{0}CN^tBu$ (3) S44

3.2. $BDFE_{N–H}$ sample calculation of second H atom abstraction between complexes $(PhH)NZr^{IV}(MesNP^iPr_2)_3Co^{0}CN^tBu$ (3) to $(PhN)Zr^{IV}(MesNP^iPr_2)_3Co^{I}CN^tBu$ (4) S44

3.3. pK_a sample calculation S45

3.4. Uncertainty in $BDFE_{N–H}$ S46

4. $BDFE_{N–H}$ test reactions S47

4.1. $(PhH_2N)Zr^{IV}(MesNP^iPr_2)_3Co^{-I}CN^tBu$ (2) with 1,8-dichloroanthraquinone S47

4.2. $(PhH)NZr^{IV}(MesNP^iPr_2)_3Co^{0}CN^tBu$ (3) with 1,8-dichloroanthraquinone S51

4.3. (PhH)NZr^{IV}(MesNP^{i}Pr$_2$)$_3$Co^{0}CN^{t}Bu (3) with *p*-benzoquinone S54
5. pK_a test reaction S58
5.1. Reaction of (PhH$_2$N)Zr^{IV}(MesNP^{i}Pr$_2$)$_3$Co^{-I}CN^{t}Bu (2) with LiN(SiMe$_3$)$_2$ S58
5.2. (PhH$_2$N)Zr^{IV}(MesNP^{i}Pr$_2$)$_3$Co^{-I}CN^{t}Bu (2) with NEt$_3$ S62
5.3. Reaction of (PhHN)Zr^{IV}(MesNP^{i}Pr$_2$)$_3$Co^{0}CN^{t}Bu (3) with LiN(SiMe$_3$)$_2$ S64
5.4. Reaction of (PhHN)Zr^{IV}(MesNP^{i}Pr$_2$)$_3$Co^{0}CN^{t}Bu (3) with NEt$_3$ S68
5.5. Reaction of [PhNZr^{IV}(MesNP^{i}Pr$_2$)$_3$Co^{I}CN^{t}Bu] (4) with [HNEt$_3$][BPh$_4$] S70
5.6. Reaction between [PhNZr^{IV}(MesNP^{i}Pr$_2$)$_3$Co^{I}CN^{t}Bu] (4) with [^{t}BuHN]P(pyrr)[BPh$_4$]. S73
6. Crystallographic Data S76
6.1 Crystallographic Data of (PhH$_2$N)Zr^{IV}(MesNP^{i}Pr$_2$)$_3$Co^{-I}CN^{t}Bu (2) S76
6.2. Crystallographic Data of (PhHN)Zr^{IV}(MesNP^{i}Pr$_2$)$_3$Co^{0}CN^{t}Bu (3) S78
6.3. Crystallographic Data of (PhN)Zr^{IV}(MesNP^{i}Pr$_2$)$_3$Co^{I}CN^{t}Bu (4) S80
6.4. Crystallographic Data of [PhHNZr^{IV}(MesNP^{i}Pr$_2$)$_3$Co^{-I}CN^{t}Bu]$^-$ (5) S82
6.5. Crystallographic Data of [PhNZr^{IV}(MesNP^{i}Pr$_2$)$_3$Co^{0}CN^{t}Bu]$^-$ (6) S84
6.6. Crystallographic Data of [PhHNZr^{IV}(MesNP^{i}Pr$_2$)$_3$Co^{I}CN^{t}Bu][BPh$_4$] (7) S86
7. Computational Details S91
8. References S93

1. Synthesis and Characterization

1.1. Characterization of (PhH$_2$N)Zr^{IV}(MesNP^{i}Pr$_2$)$_3$Co^{-I}CN^{t}Bu (2)

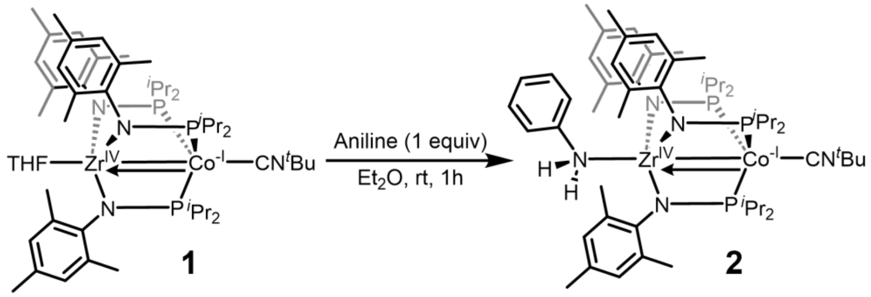

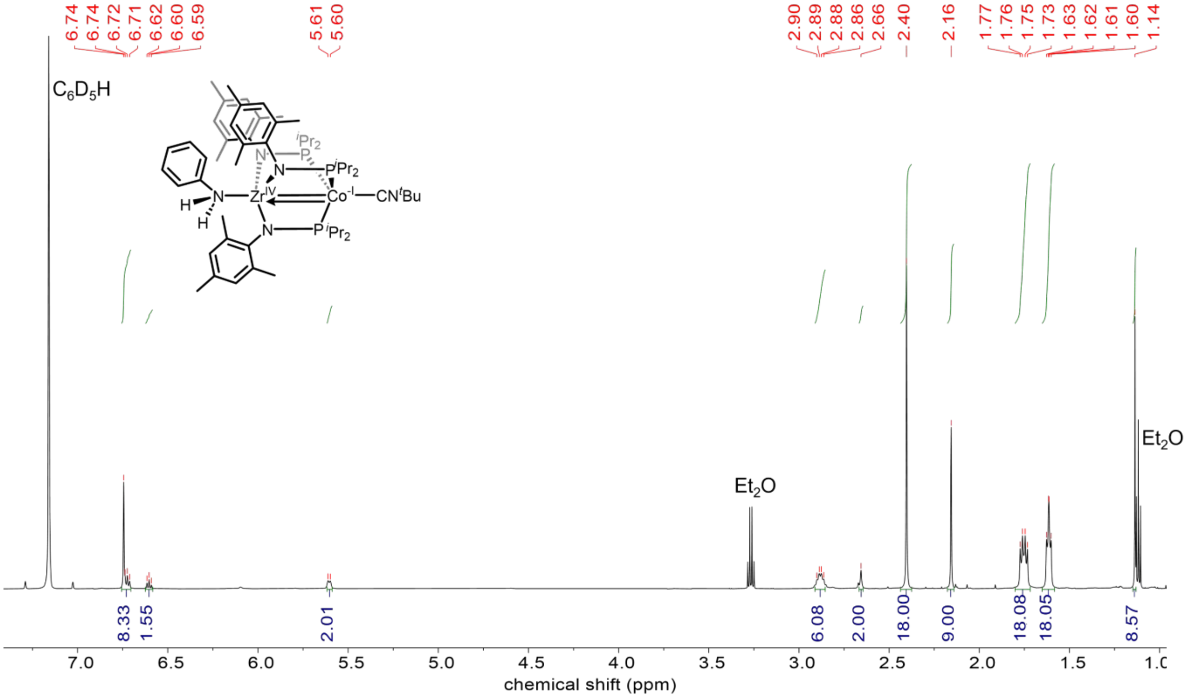

Figure S1. ^{1}H NMR spectrum (600MHz, C_6D_6) of (PhH$_2$N)Zr^{IV}(MesNP^{i}Pr$_2$)$_3$Co^{-I}CN^{t}Bu (**2**).

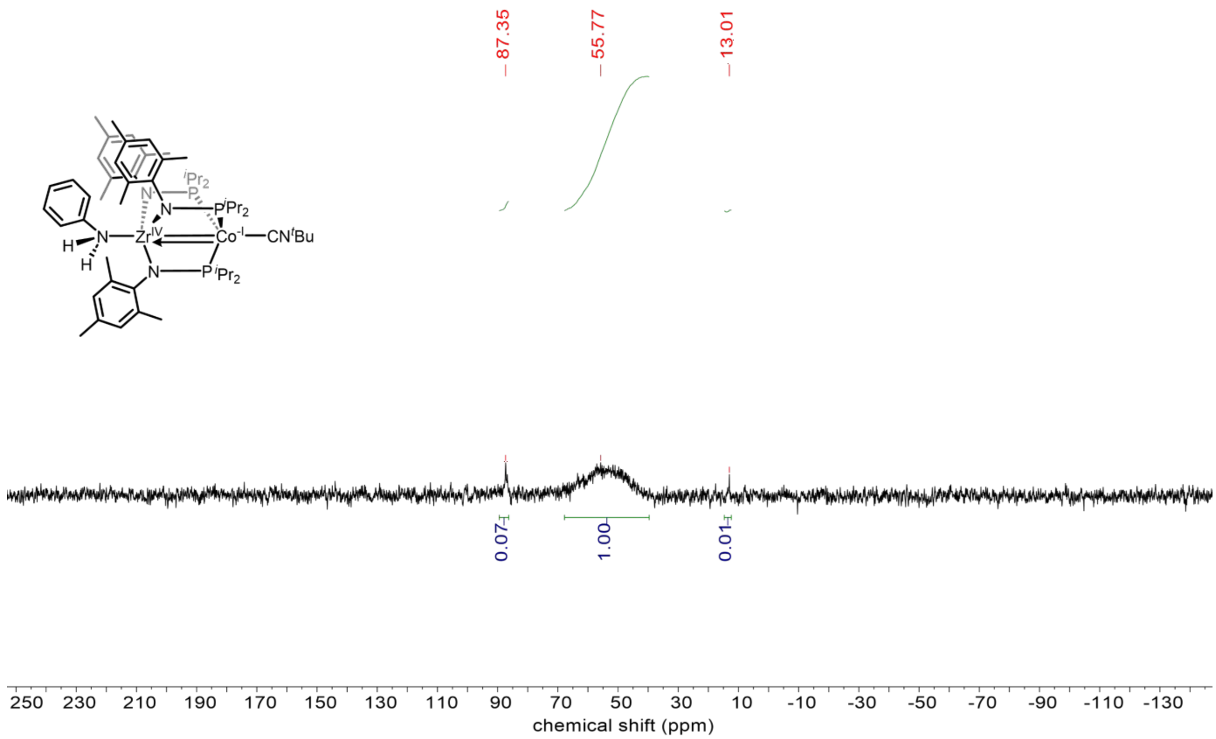

Figure S2. $^{31}P\{^{1}H\}$ NMR spectrum (162 MHz, C_6D_6) of $(PhH_2N)Zr^{IV}(MesNP^iPr_2)_3Co^{-I}CN^tBu$ (**2**). Broad signal from 40-70 ppm corresponds to compound **2**. Sharper resonances with much smaller integrations are also observed at 87.35 ppm and 13.01 ppm, although no additional diamagnetic species are observed by ^{1}H NMR. It is hypothesized that that those resonances correspond to an impurity generated upon binding of a second isocyanide ligand to complex **2**.

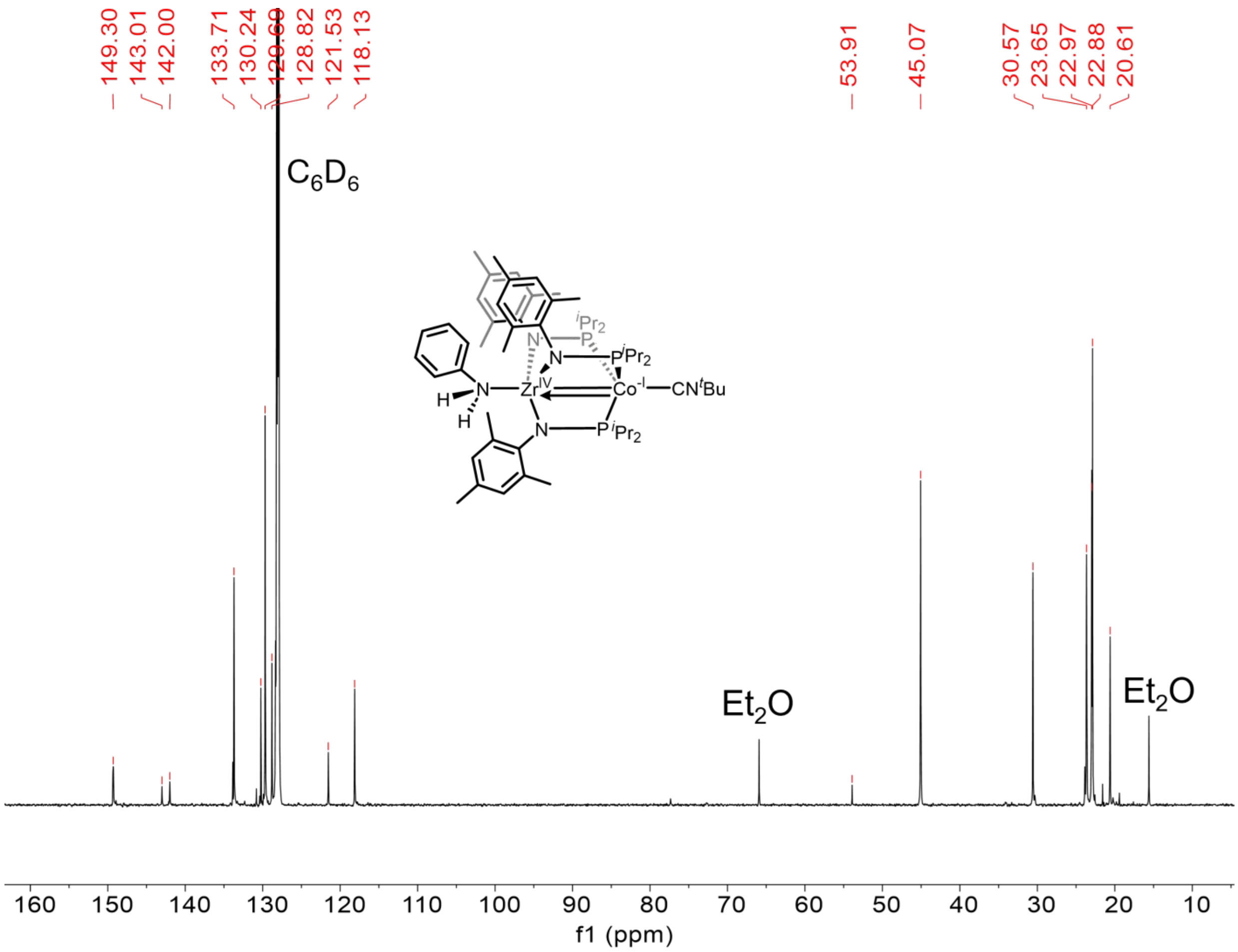

Figure S3. $^{13}C\{^1H\}$ NMR spectrum (176 MHz, C_6D_6) of $(PhH_2N)Zr^{IV}(MesNP^iPr_2)_3Co^{-I}CN^tBu$ (**2**).

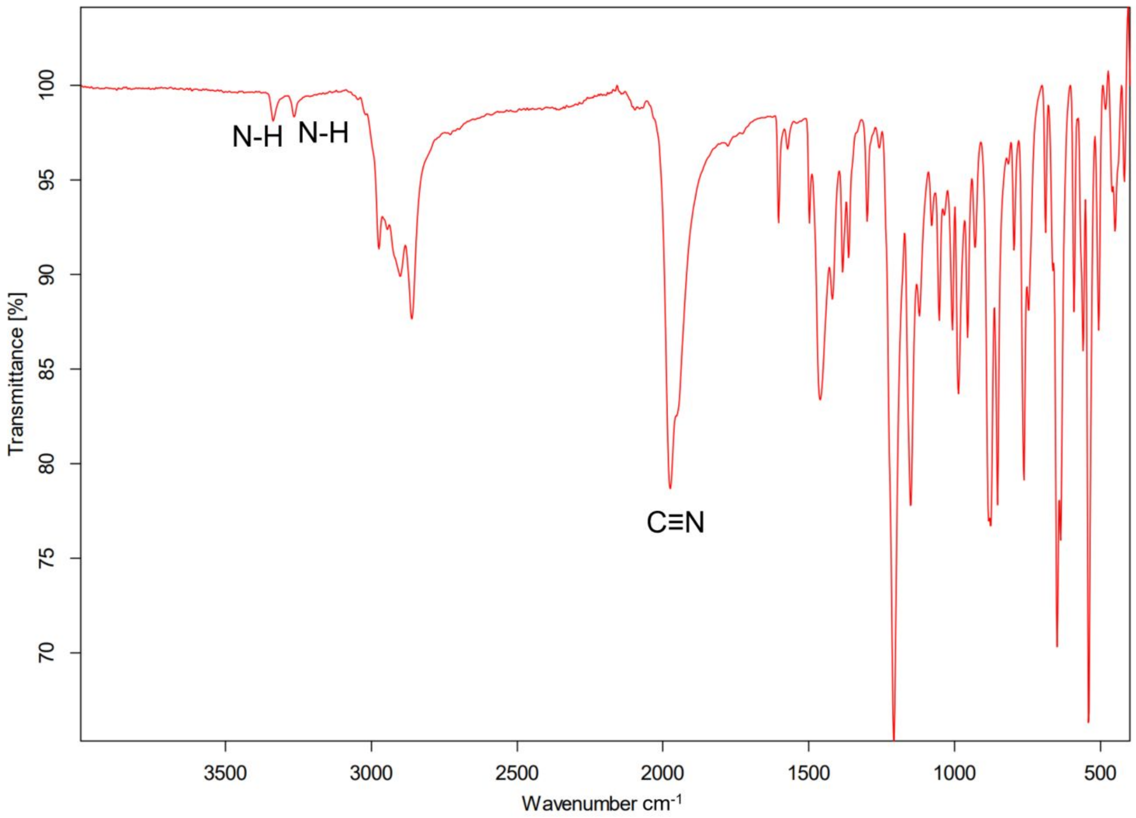

Figure S4. Solid-state (ATR) IR spectrum of $(PhH_2N)Zr^{IV}(MesNP^iPr_2)_3Co^{-I}CN^tBu$ (**2**).

1.2. Characterization of (PhHN)Zr^{IV}(MesNP^{i}Pr$_2$)$_3$Co^{0}CN^{t}Bu (3)

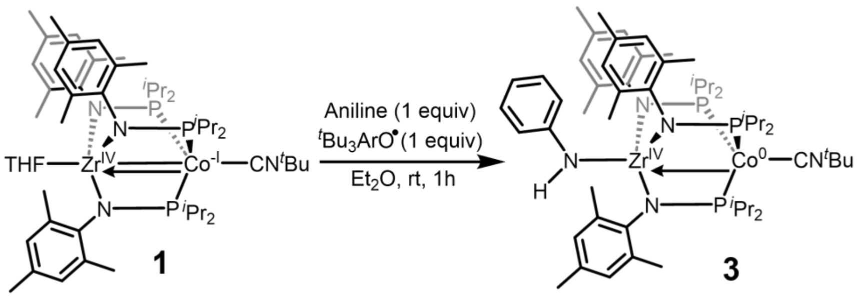

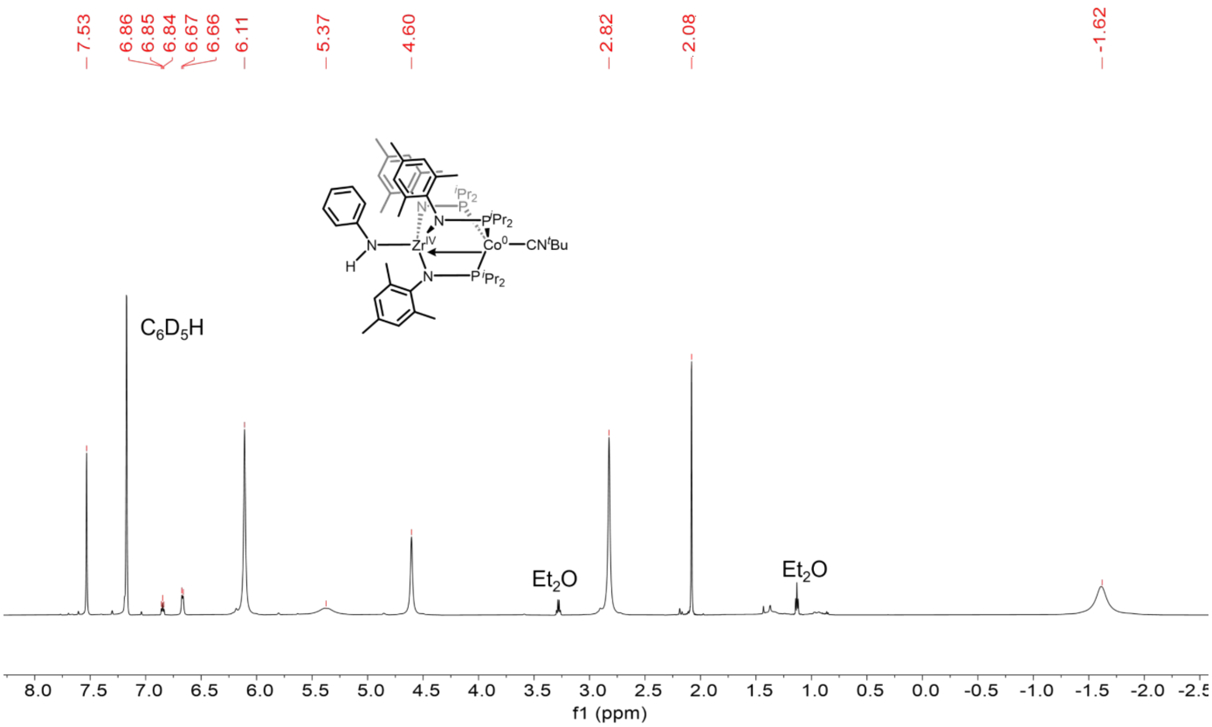

Figure S5. ^{1}H NMR spectrum (600 MHz, C$_6$D$_6$) of (PhHN)Zr^{IV}(MesNP^{i}Pr$_2$)$_3$Co^{0}CN^{t}Bu (**3**).

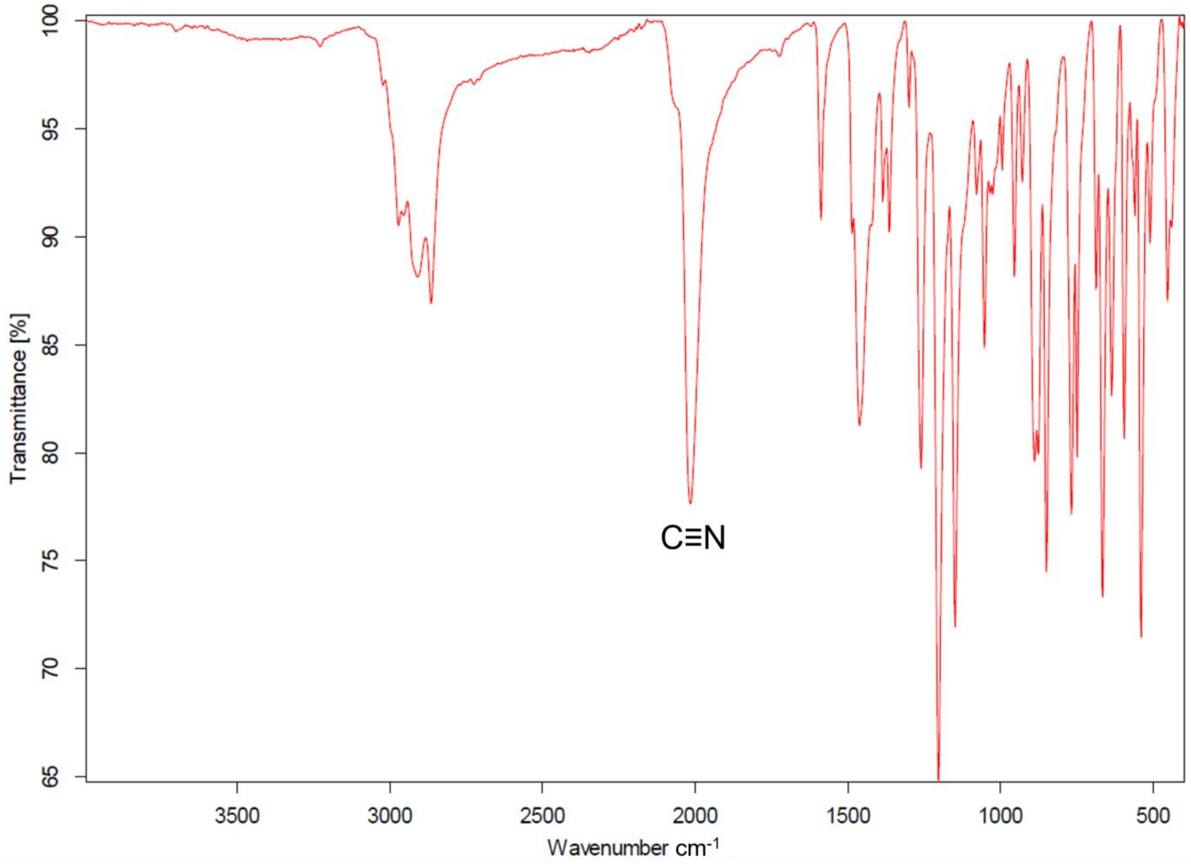

Figure S6. Solid-state (ATR) IR spectrum of (PhHN)Zr^{IV}(MesNP^{i}Pr_2)$_3$$Co^0$CN^{t}Bu (**3**).

1.3. Characterization of (PhN)Zr^{IV}(MesNP^{i}Pr$_2$)$_3$Co^{I}CN^{t}Bu (4)

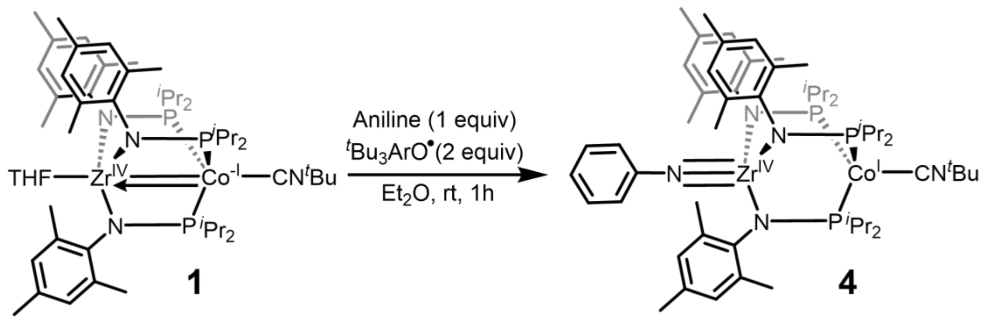

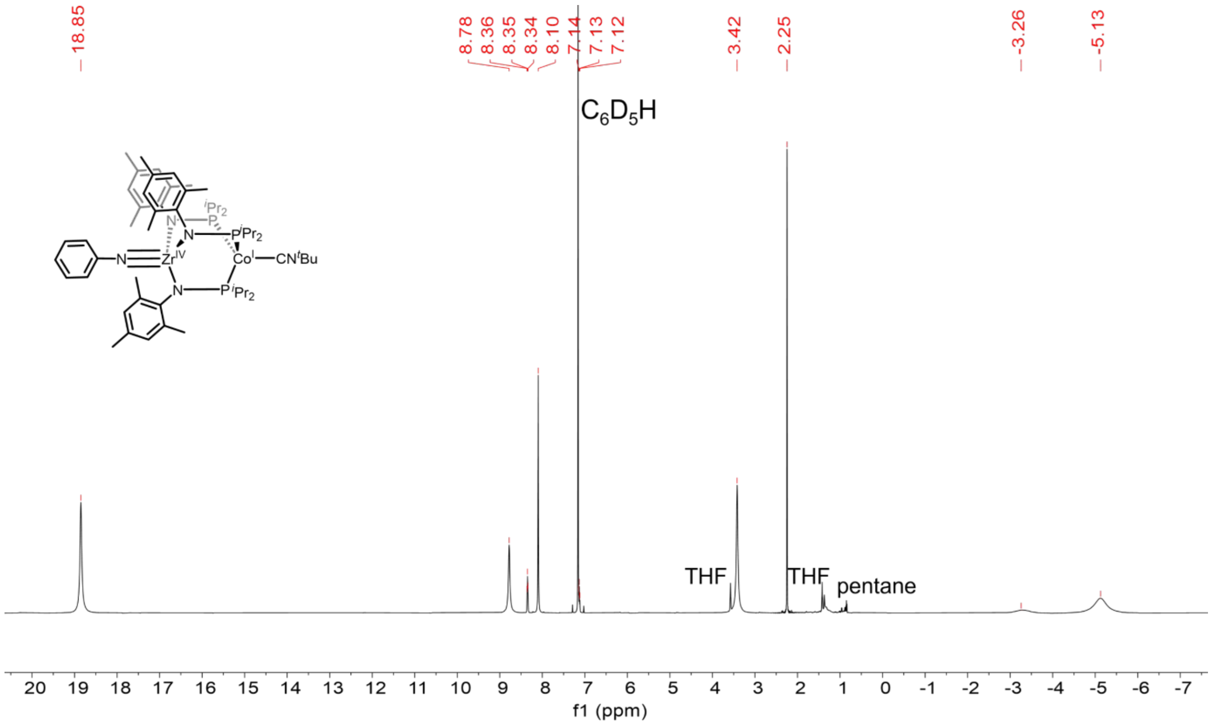

Figure S7. ^{1}H NMR spectrum (600MHz, C_6D_6) of (PhN)Zr^{IV}(MesNP^{i}Pr$_2$)$_3$Co^{I}CN^{t}Bu (**4**).

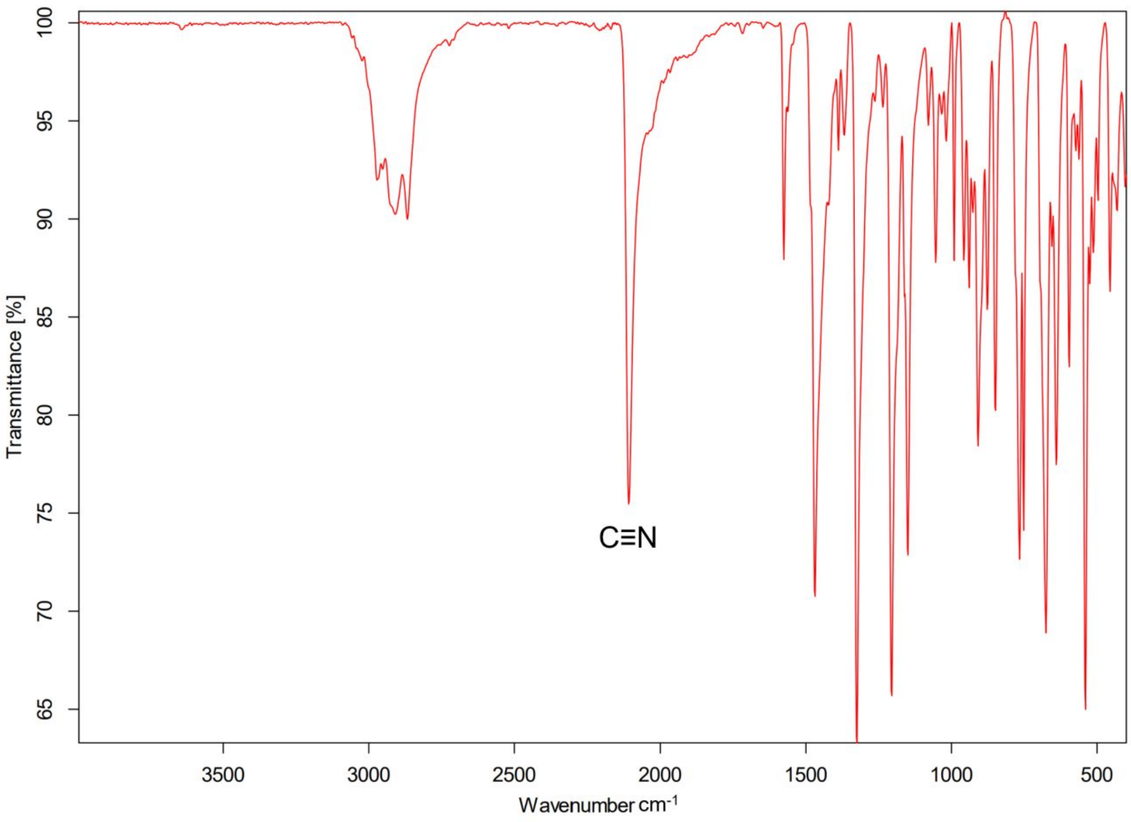

Figure S8. Solid-state (ATR) IR spectrum of (PhN)Zr^{IV}(MesNP^{i}Pr_2)$_3$$Co^{I}$CN^{t}Bu (**4**).

1.4. Characterization of [PhHNZr^{IV}(MesNP^{i}Pr$_2$)$_3$Co^{-I}CN^{t}Bu]$^-$ (5)

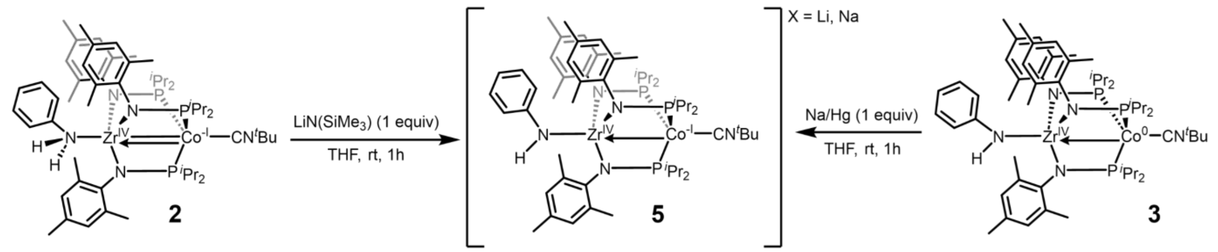

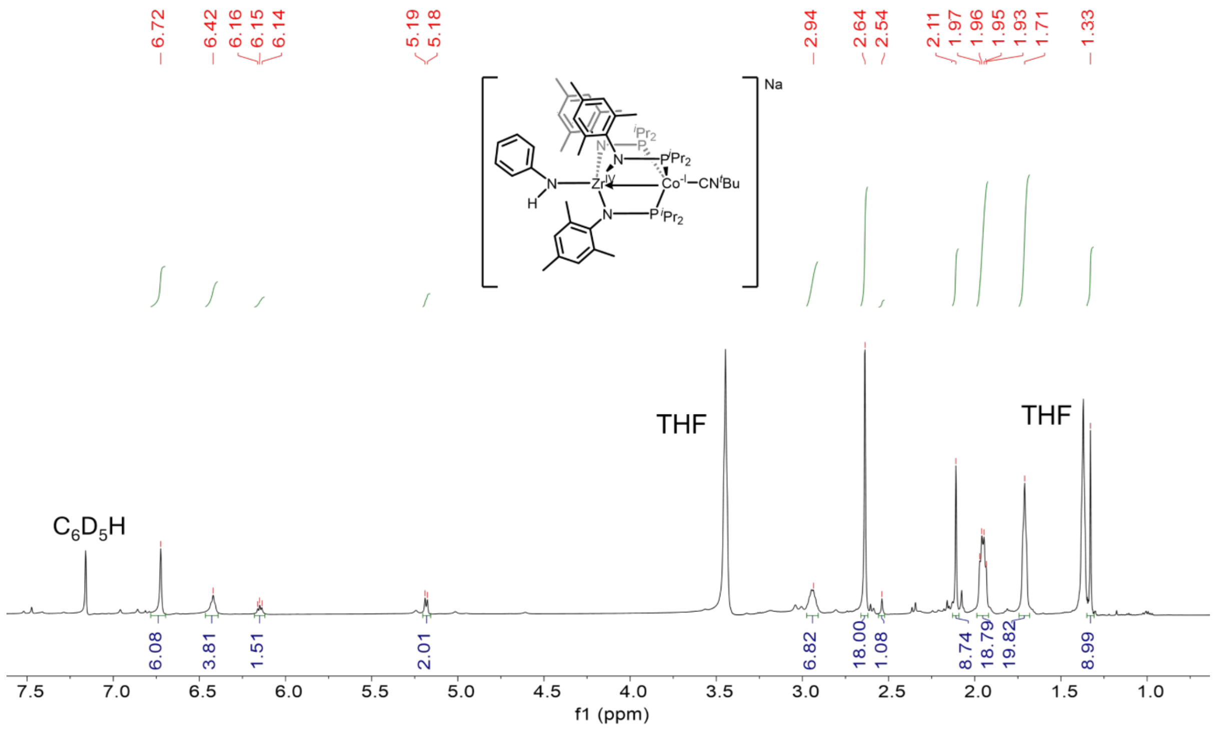

Figure S9. ^{1}H NMR spectrum (600 MHz, C_6D_6) of [PhHNZr^{IV}(MesNP^{i}Pr$_2$)$_3$Co^{-I}CN^{t}Bu]$^-$ (**5**).

1.5. Characterization of $[PhNZr^{IV}(MesNP^iPr_2)_3Co^0CN^tBu]^-$ (6)

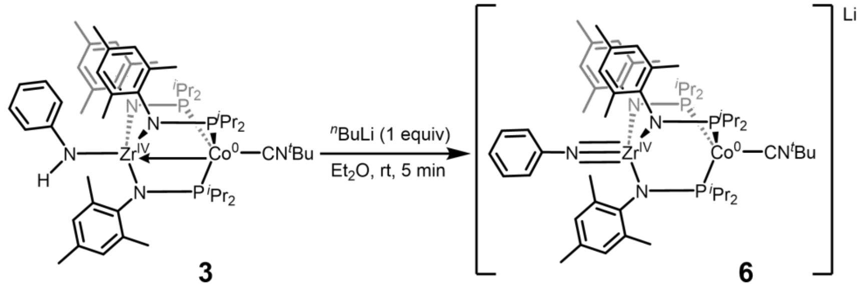

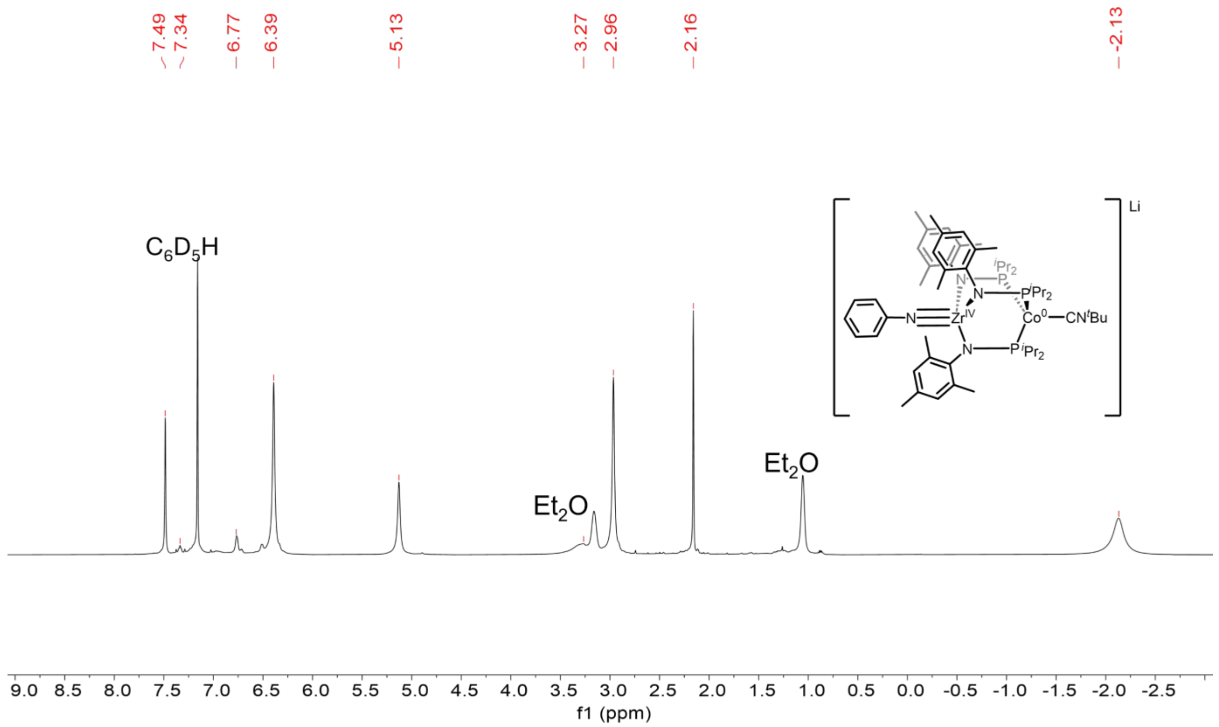

Figure S10. ^{1}H NMR spectrum (600 MHz, C_6D_6) of $[PhNZr^{IV}(MesNP^iPr_2)_3Co^0CN^tBu]^-$ (**6**).

1.6. Characterization of [PhHNZr^{IV}(MesNP^{i}Pr$_2$)$_3$Co^{I}CN^{t}Bu][BPh$_4$] (7)

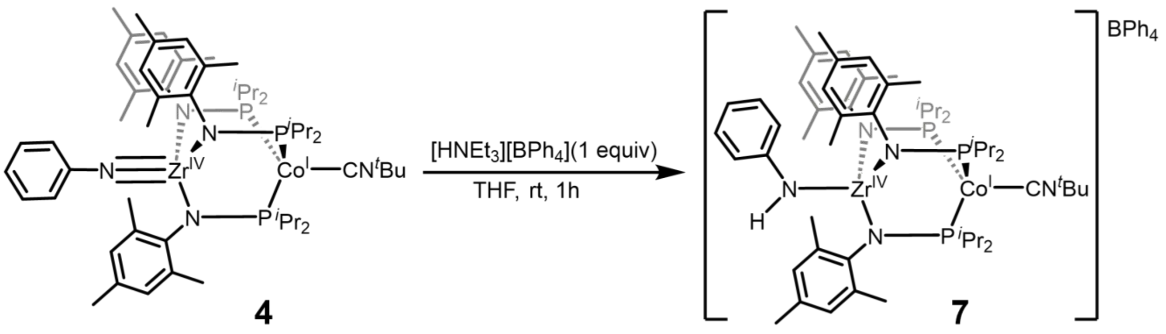

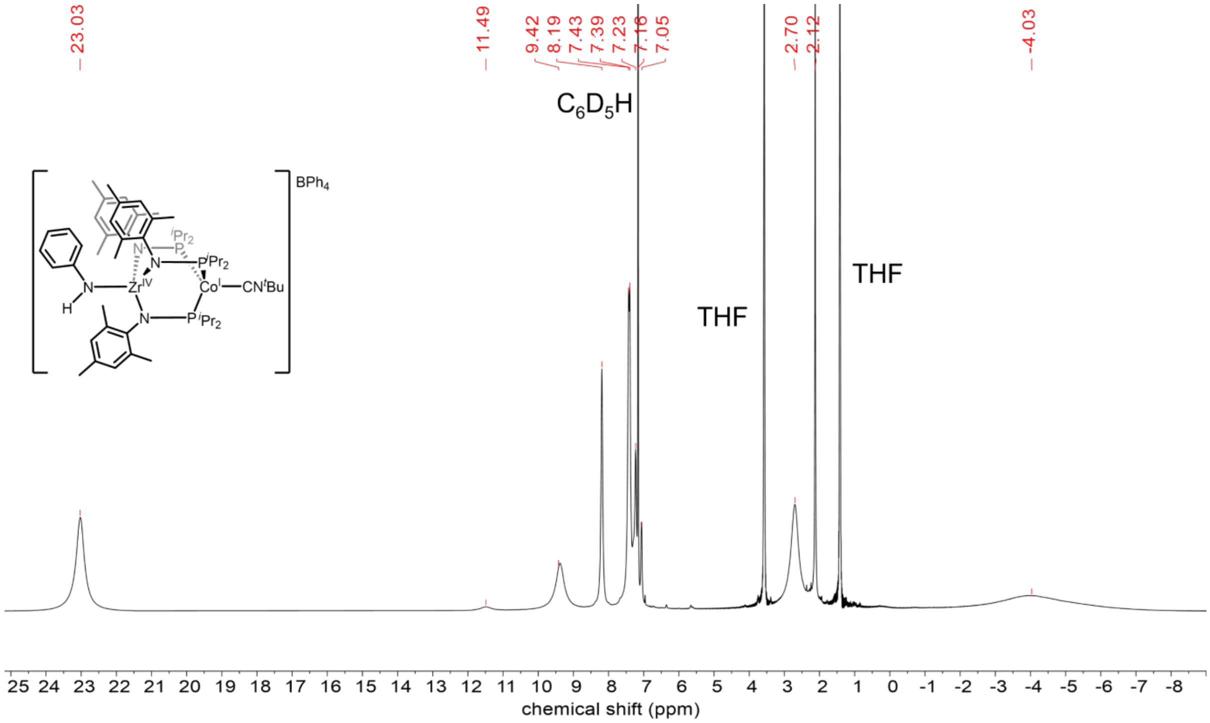

Figure S11. ^{1}H NMR spectrum (400 MHz, C_6D_6) of [PhHNZr^{IV}(MesNP^{i}Pr$_2$)$_3$Co^{I}CN^{t}Bu][BPh$_4$] (**7**).

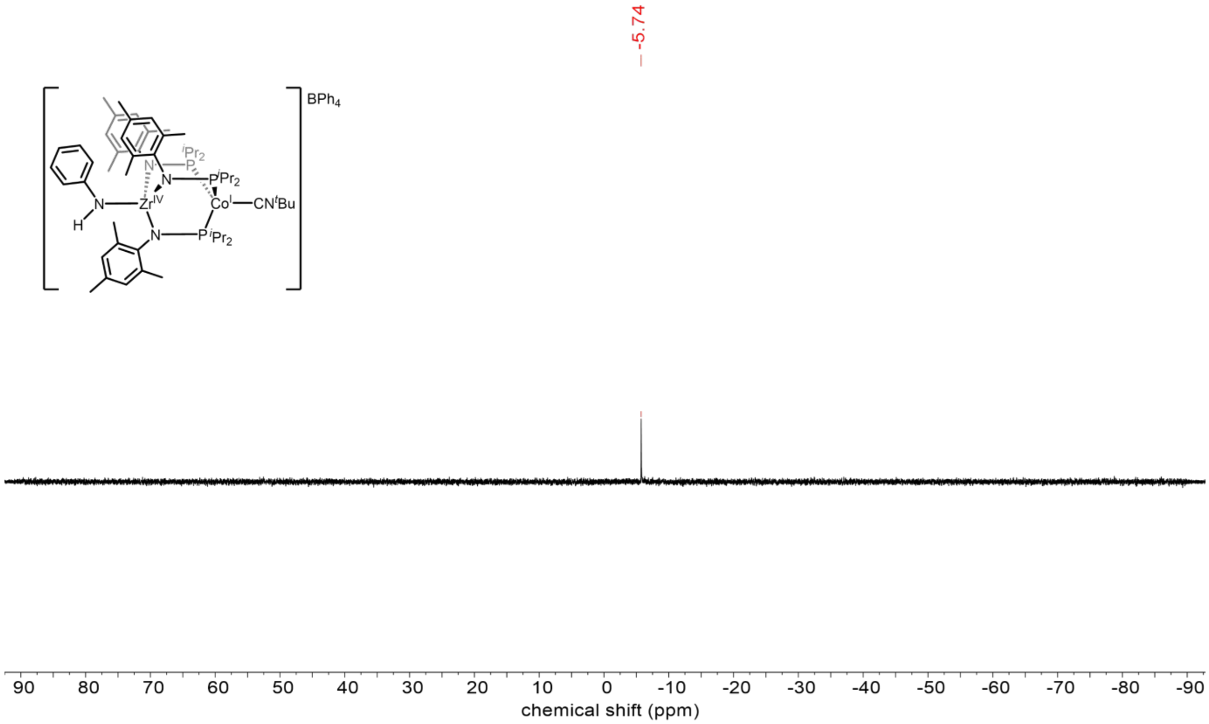

Figure S12. ^{11}B NMR spectrum (128 MHz, C_6D_6) of [PhHNZr^{IV}(MesNP^{i}Pr$_2$)$_3$Co^{I}CN^{t}Bu][BPh$_4$] (**7**).

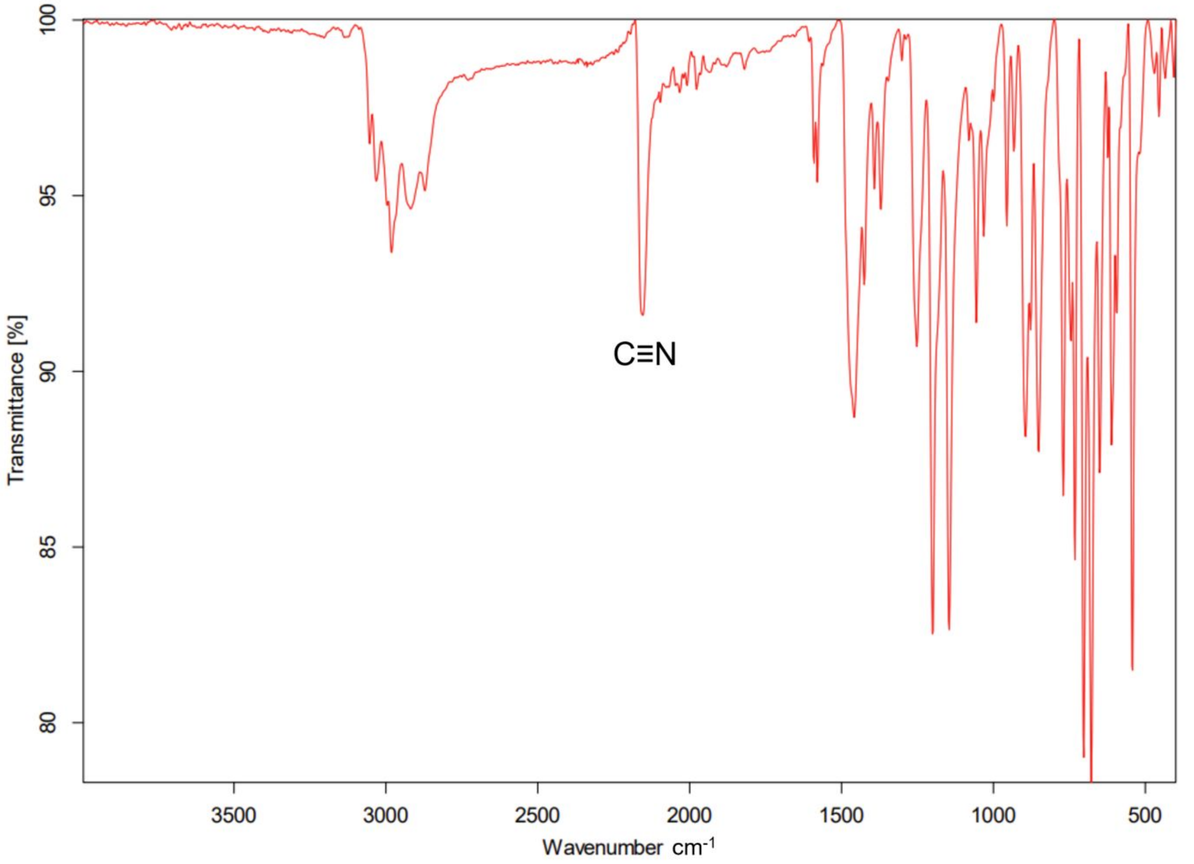

Figure S13. Solid-state (ATR) IR spectrum of [PhHNZr^{IV}(MesNP^{i}Pr$_2$)$_3$Co^{I}CN^{t}Bu][BPh$_4$] (**7**).

2. Cyclic Voltammetry & Open-Circuit Potential Measurements

Open-circuit potential (OCP) and cyclic voltammetry (CV) experiments were performed in a nitrogen-filled glovebox, unless otherwise noted, using a CHI 620E potentiostat (CH instruments Inc., Austin TX). The Pt wire (counter electrode) was flame-dried using a Bunsen burner. The glassy carbon electrode (working electrode) was polished with MicroPolish Powder (0.05 micron), rinsed with DI water, and then dried carefully with a Kimwipe prior to use. The Ag/$AgNO_3$ pseudoreference electrode was prepared in THF by stirring 10 mM $AgNO_3$ solution in 100 mM electrolyte solution ([^{n}Bu_4N][PF_6]) overnight. An additional 20 mL of 100 mM electrolyte solution ([^{n}Bu_4N][PF_6]) was also prepared and stored over freshly activated 3 Å molecular sieves overnight. The $AgNO_3$ solution was filtered prior to filling the reference electrode chamber. The electrode was then rinsed and stored in the 20 mL 100 mM ([^{n}Bu_4N][PF_6]) electrolyte solution.

2.1. $E°_{OCP}$ (NH_2/NH) vs H_2

In a nitrogen-filled glovebox, OCP measurements between the amino and amido complexes ($PhH_2NZr^{IV}(MesNP^iPr_2)_3Co^{-I}CN^tBu$ (**2**) and $PhHNZr^{IV}(MesNP^iPr_2)_3Co^{0}CN^tBu$ (**3**), respectively) were collected following published procedures.[1,2] An electrolyte/buffer stock solution of 100 mM [^{n}Bu_4N][PF_6], 50 mM lutidine, and 50 mM [Hlut][BPh_4] in THF was prepared using a 100 mL volumetric flask via the following procedure: [^{n}Bu_4N][PF_6], lutidine, and [Hlut][BPh_4] were measured in separate scintillation vials, dissolved in the electrolyte/buffer solution, and transferred to the volumetric flask. The electrolyte/buffer stock solution was stored over 3 Å molecular sieves prior to use. Two separate stock solutions of complexes **2** and **3** were prepared using 5 mL and 15 mL volumetric flasks, respectively, using the prepared electrolyte/buffer solution to dilute to the mark. Complex **3** was insoluble in 5 mL of the electrolyte/buffer solution, which was the basis of using the 15 mL volumetric flask instead. Solutions of desired the concentrations of **2** and **3** were prepared using the stock solutions described above. A glass syringe was used to deliver the appropriate volumes of the **2** and **3** stock solutions into a 5 mL volumetric flask. The same electrolyte/buffer stock solution was used to dilute the mark.

In an electrochemical cell containing the glassy carbon electrode (working electrode), the Ag^{+}/Ag pseudoreference electrode, and the Pt wire (counter electrode), 5 mL of the amino (**2**)/ amido (**3**) solution was added. OCP measurements were collected using five ratios of **2**:**3** (approximately 1.0:1.5, 1.25:1.5, 1.5:1.5, 1.75:1.5, 2.0:1.5, respectively). While stirring, OCP measurements were collected in 1 s intervals for 400 s. After each OCP run, the stirring was stopped, and Fc was added to the electrochemical cell to collect a CV to reference the NH_2/NH OCP vs $Fc^{+/0}$. The $E°_{OCP}$ (NH_2/NH) vs $Fc^{+/0}$ was then referenced to the E_{OCP} (H^+/H_2) (-0.469 V vs $Fc^{+/0}$) previously reported under the same conditions.[1] The OCP measurement at each **2**:**3** ratio was repeated to confirm reproducibility.

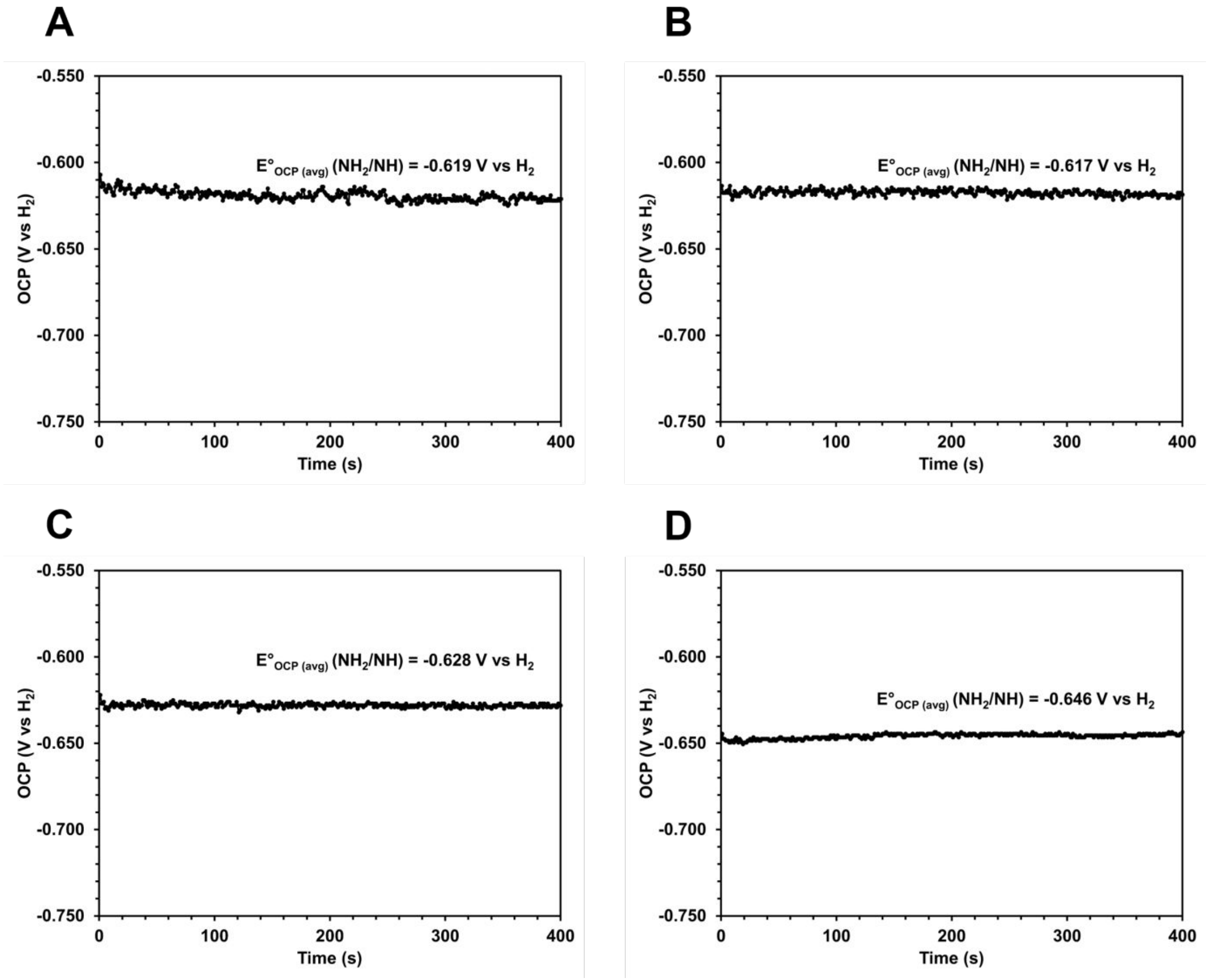

Figure S14. Trial 1: OCP (V vs H_2) over time of THF solutions containing 100 mM [$^n Bu_4N$][PF_6], 50 mM lutidine, 50 mM of [Hlut][BPh_4], and varying ratios of **2** and **3**. The OCP was measured every 1 s for 400 s. Each ratio is represented as follows: A) 1.22:1.45, B) 1.46:1.45, C) 1.70:1.45, and D) 1.94:1.45.

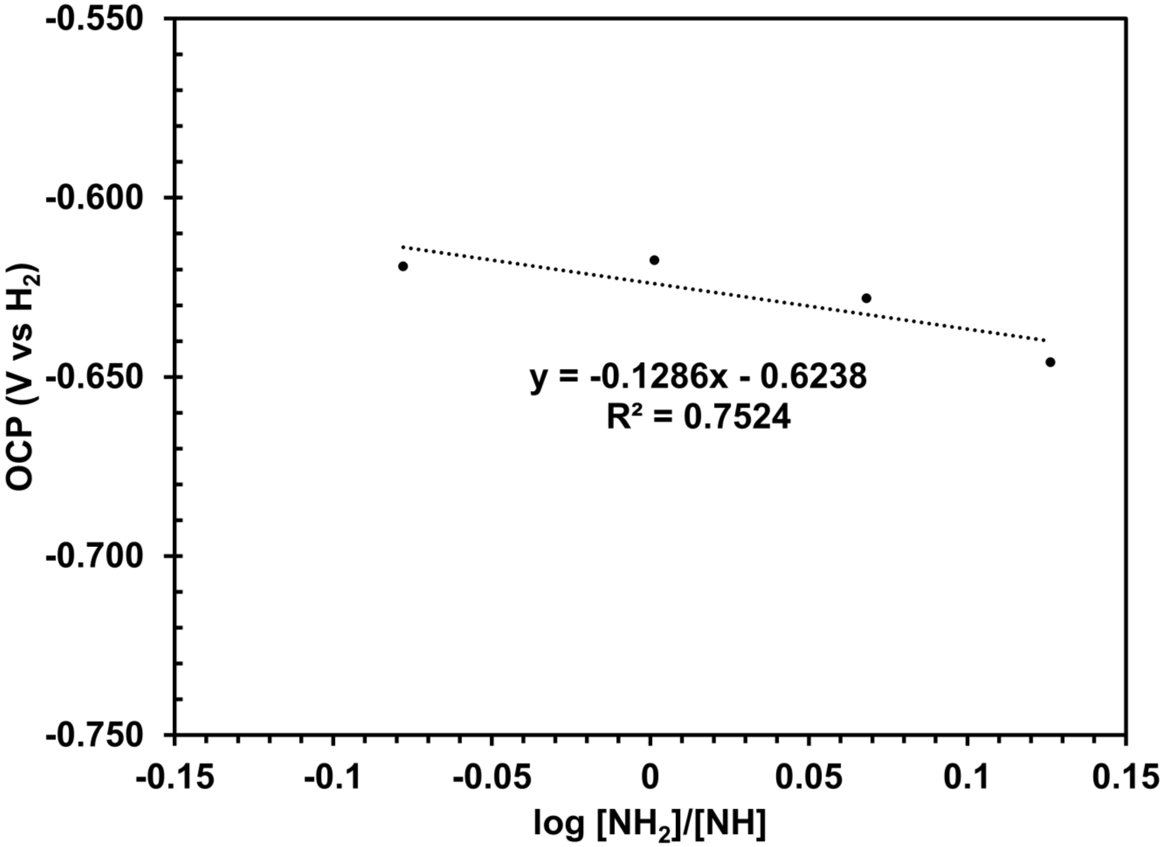

Figure S15. Trial 1: OCP (V vs H_2) vs. the log of varying ratios of complexes **2** and **3**. OCPs were determined by using the $E°_{OCP(avg)}$ (NH_2/NH) values at each given ratio from Figure S14.

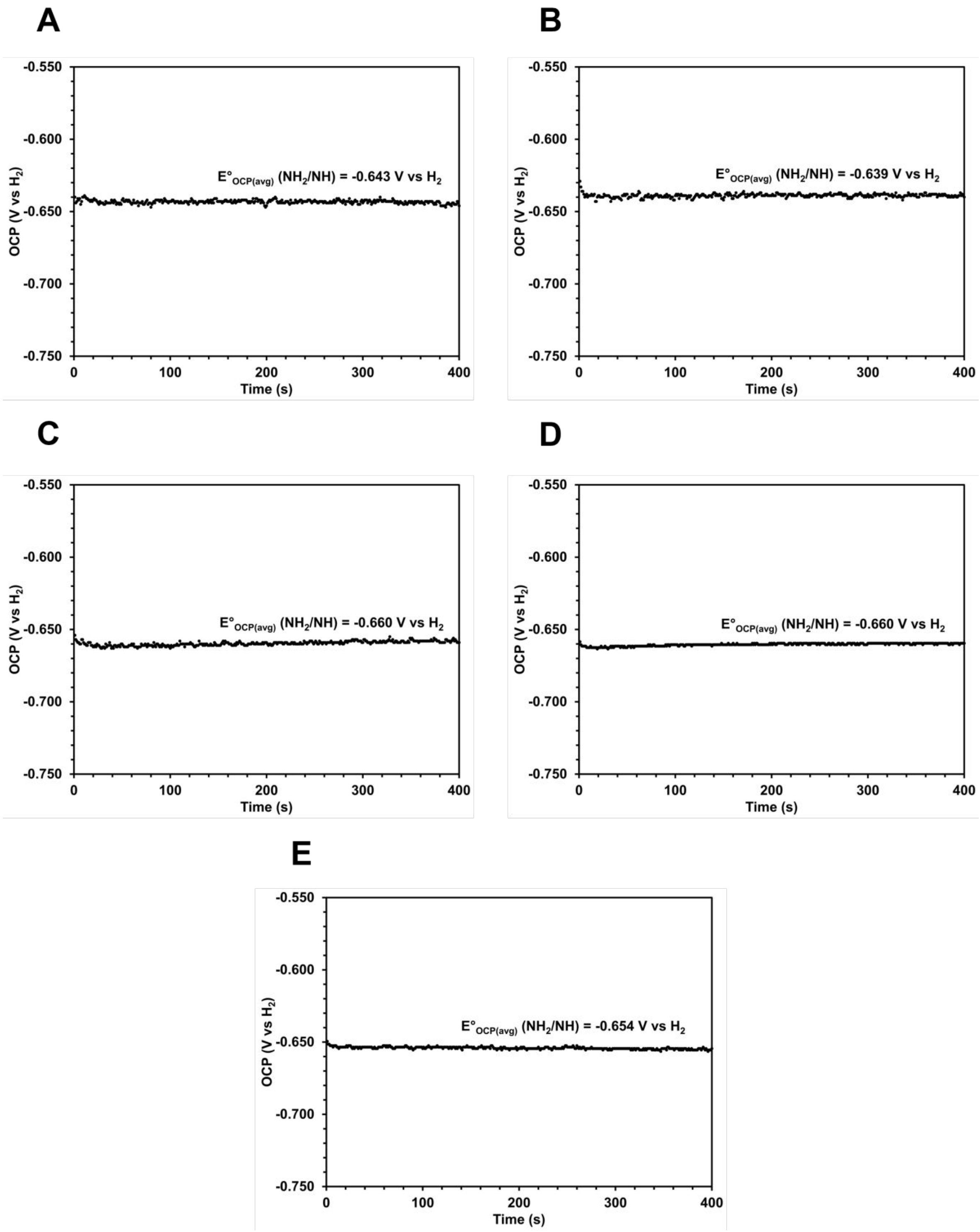

Figure S16. Trial 2: OCP (V vs H_2) over time of THF solutions containing 100 mM [^{n}Bu$_4$N][PF$_6$], 50 mM lutidine, 50 mM of [Hlut][BPh$_4$], and varying ratios of **2** and **3**. The OCP was measured every 1 s for 400 s. Each ratio is represented as follows: A) 0.97:1.45, B) 1.22:1.45, C) 1.46:1.45, D) 1.70:1.45, and E) 1.94:1.45.

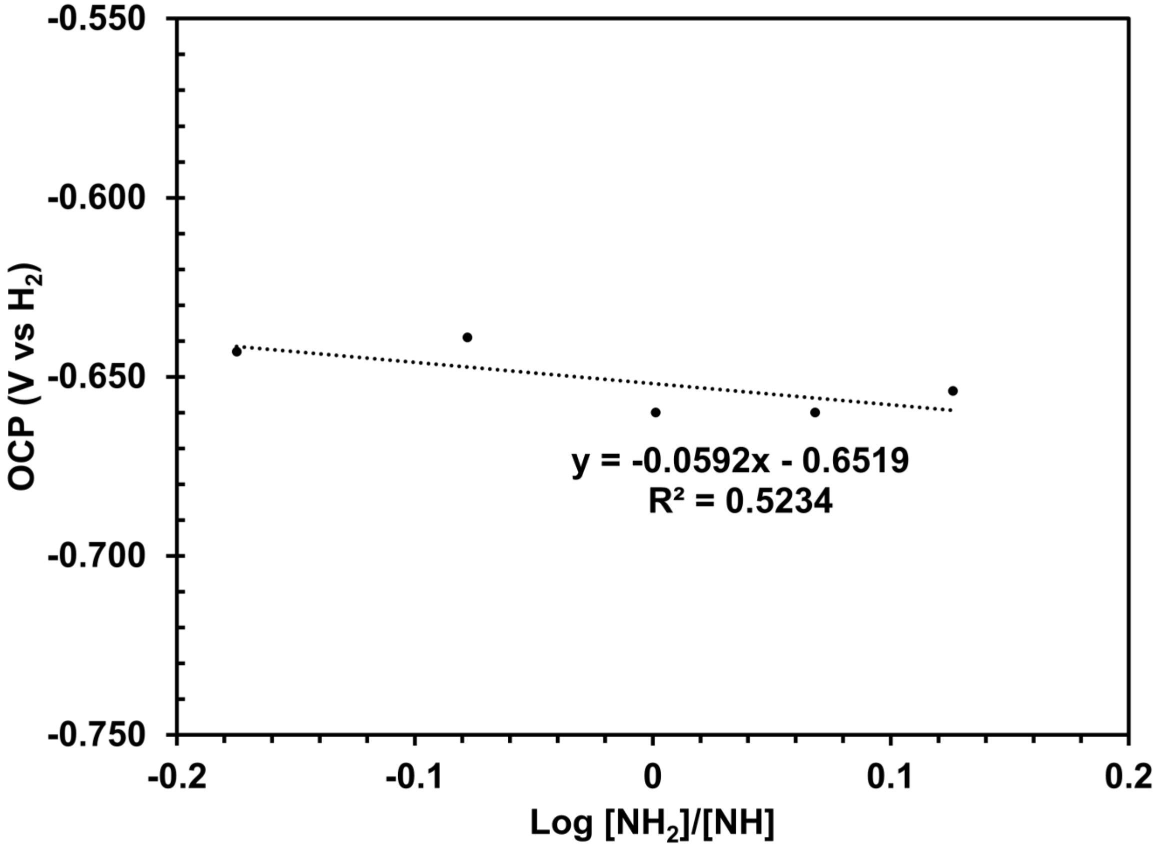

Figure S17. Trial 2: OCP (V vs H_2) vs. the log of varying ratios of complexes **2** and **3**. OCPs were determined by using the $E°_{OCP(avg)}$ (NH_2/NH) values at each given ratio from Figure S16.

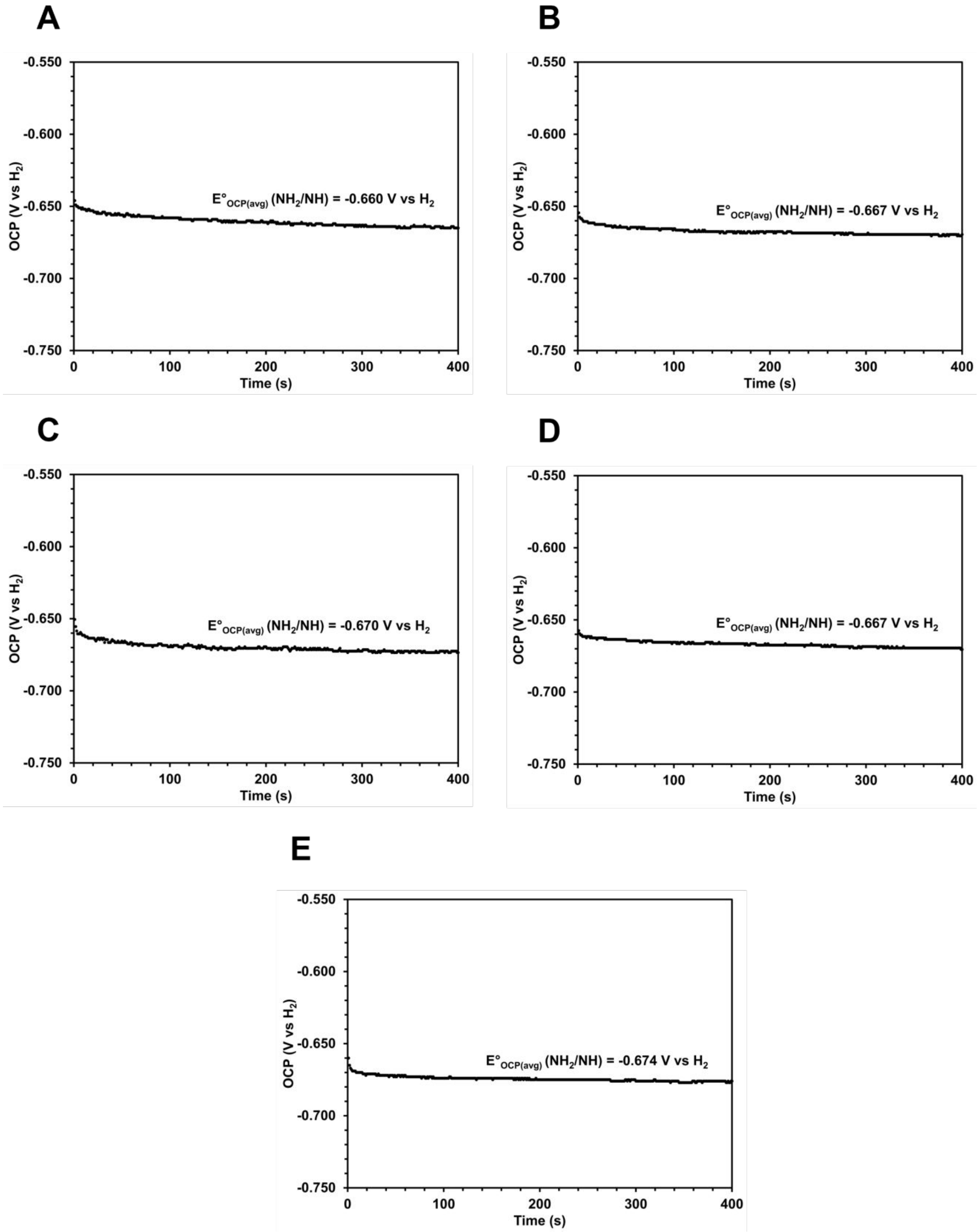

Figure S18. Trial 3: OCP (V vs H_2) over time of THF solutions containing 100 mM [^{n}Bu$_4$N][PF$_6$], 50 mM lutidine, 50 mM of [Hlut][BPh$_4$], and varying ratios of **2** and **3**. The OCP was measured every 1 s for 400 s. Each ratio is represented as follows: A) 0.97:1.45, B) 1.22:1.45, C) 1.46:1.45, D) 1.70:1.45, and E) 1.94:1.45.

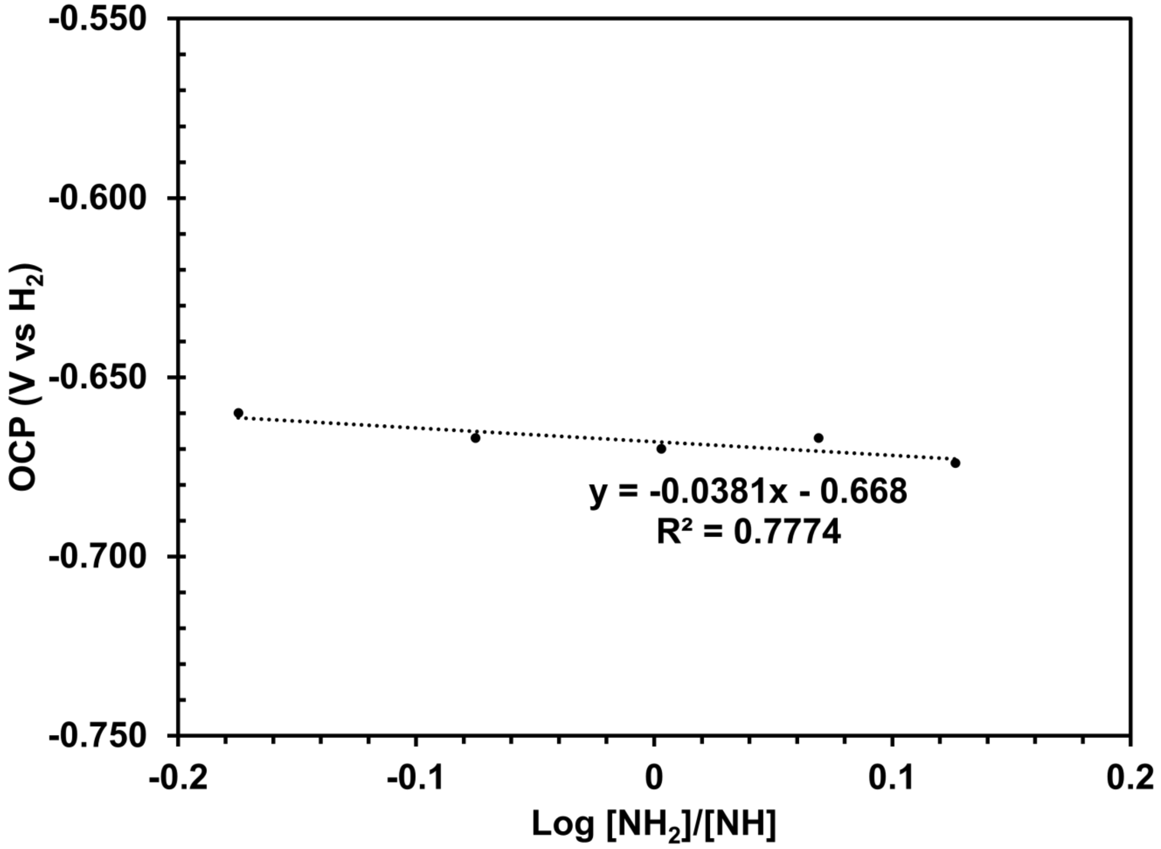

Figure S19. Trial 3: OCP (V vs H_2) vs. the log of varying ratios of complexes **2** and **3**. OCPs were determined by using the $E°_{OCP(avg)}$ (NH_2/NH) values at each given ratio from Figure S18.

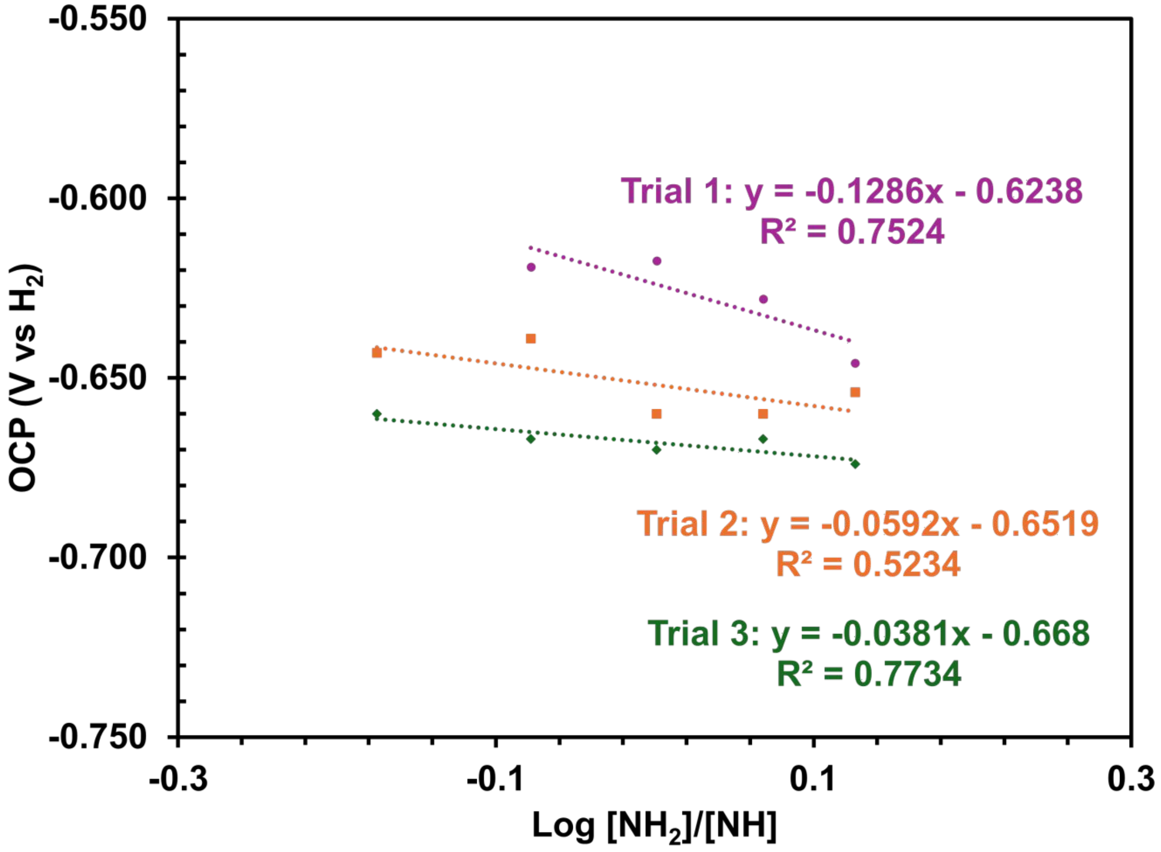

Figure S20. Summary of the OCP (V vs H_2) over the log of varying ratio of complexes **2** and **3**. OCP were determined by using the $E°_{OCP(avg)}$ (NH_2/NH) values at each given ratio from Figure S14, Figure S16, Figure S18.

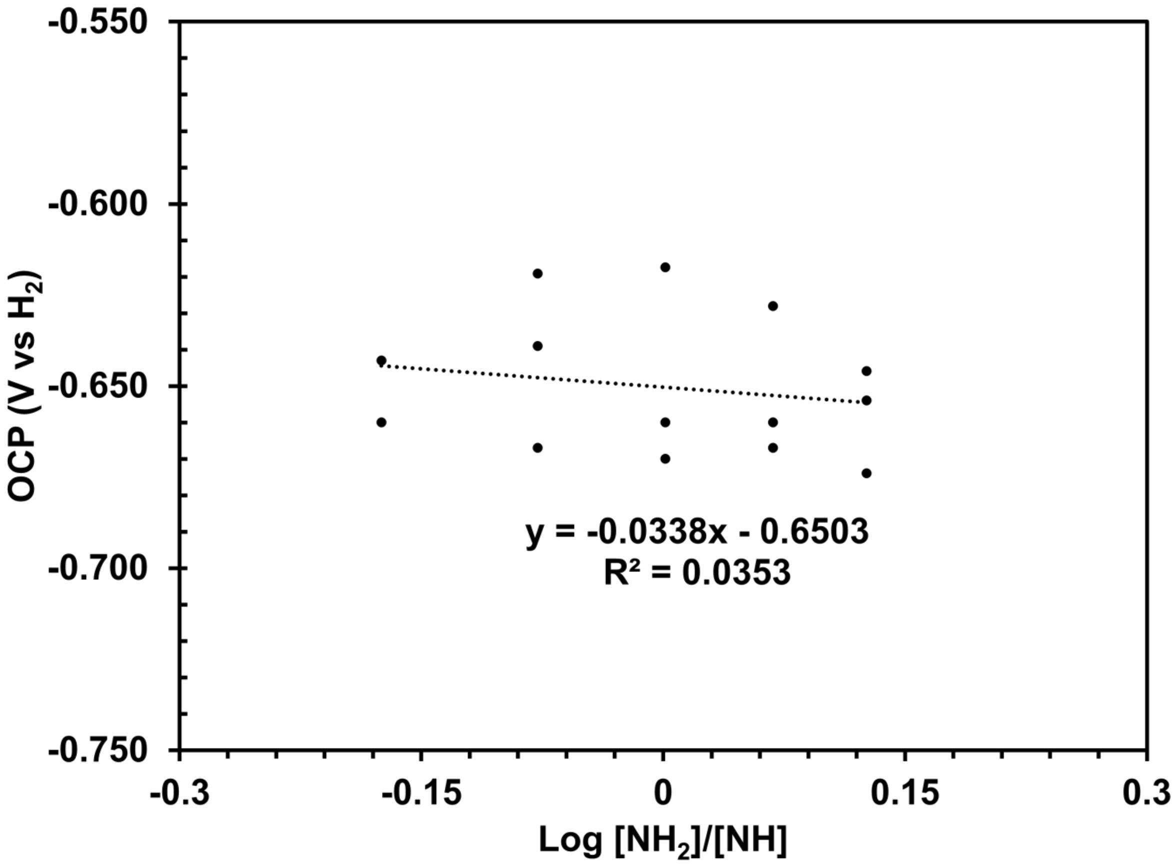

Figure S21. Data points for all three trials combined into a single plot of the OCP (V vs H_2) vs the log of varying ratios of complexes **2** and **3**. OCPs were determined by using the $E°_{OCP(avg)}$ (NH_2/NH) values at each given ratio from Figure S14, Figure S16, and Figure S18.

2.2. $E°_{OCP}$ (NH/N) vs H_2

In a nitrogen-filled glovebox, the same experimental procedures described in Section 2.1. were performed to obtain OCP measurements between the amido and imido complexes $PhHNZr^{IV}(MesNP^iPr_2)_3Co^{-I}CN^tBu$ (**3**) and $PhHNZr^{IV}(MesNP^iPr_2)_3Co^{0}CN^tBu$ (**4**), respectively. However, a different buffer system (50 mM $[HNEt_3][BPh_4]$, 50 mM NEt_3) was used instead due to unwanted reactivity of **4** with the previous buffer (50 mM [Hlut]$[BPh_4]$, 50 mM lutidine). The Hlut:lut buffer was found to protonate complex **4**, generating **7** (Figure S22, S23, and S24). The Mayer group reported that side reactions of the required substrate, under experimental conditions, are possible sources of error during OCP measurements that affects accurately determining the BDFE.[2] Thus, a different buffer (50 mM $[HNEt_3][BPh_4]$, 50 mM NEt_3) that was unreacted to **4** was used as well as its corresponding E_{OCP} (H^+/H_2) (-1.148(7) V vs $Fc^{+/0}$) obtained under the same electrolyte and buffer conditions by the Mayer group.[2]

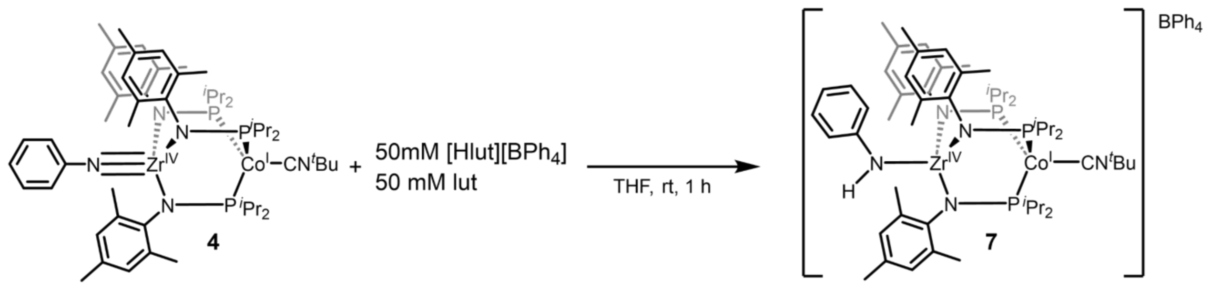

A buffer solution containing 50 mM [Hlut][BPh_4] and 50 mM lutidine solution was prepared by massing out [Hlut][BPh_4] (108.0 mg, 0.2527 mmol) and lutidine (33.6 mg, 0.3136 mmol) and dissolving in 5 mL of THF using a 5 mL volumetric flask. The buffer solution was then added to **4** (9.9 mg, 0.0092 mmol) and set to stir. No noticeable color change was observed. The reaction mixture was allowed to stir at room temperature for 1 hour. The volatiles were removed under vacuum prior to ^{1}H NMR analysis. Although the expected outcome was no reactivity between the buffer and **4**, the resulting ^{1}H NMR spectrum revealed complete conversion to **7**.

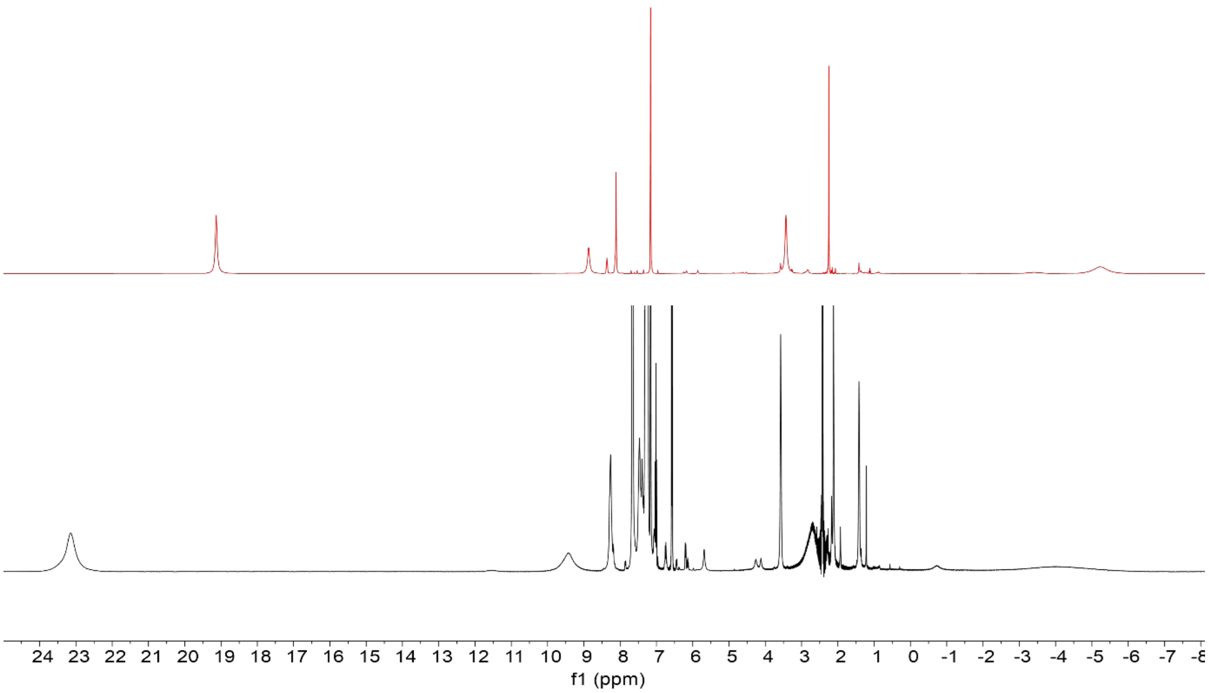

Figure S22. ^{1}H NMR spectra (400 MHz, C_6D_6) of **4** (top, red) and the crude reaction between **4** and 50 mM [Hlut][BPh_4], 50 mM lutidine after 1 h of stirring (bottom, black).

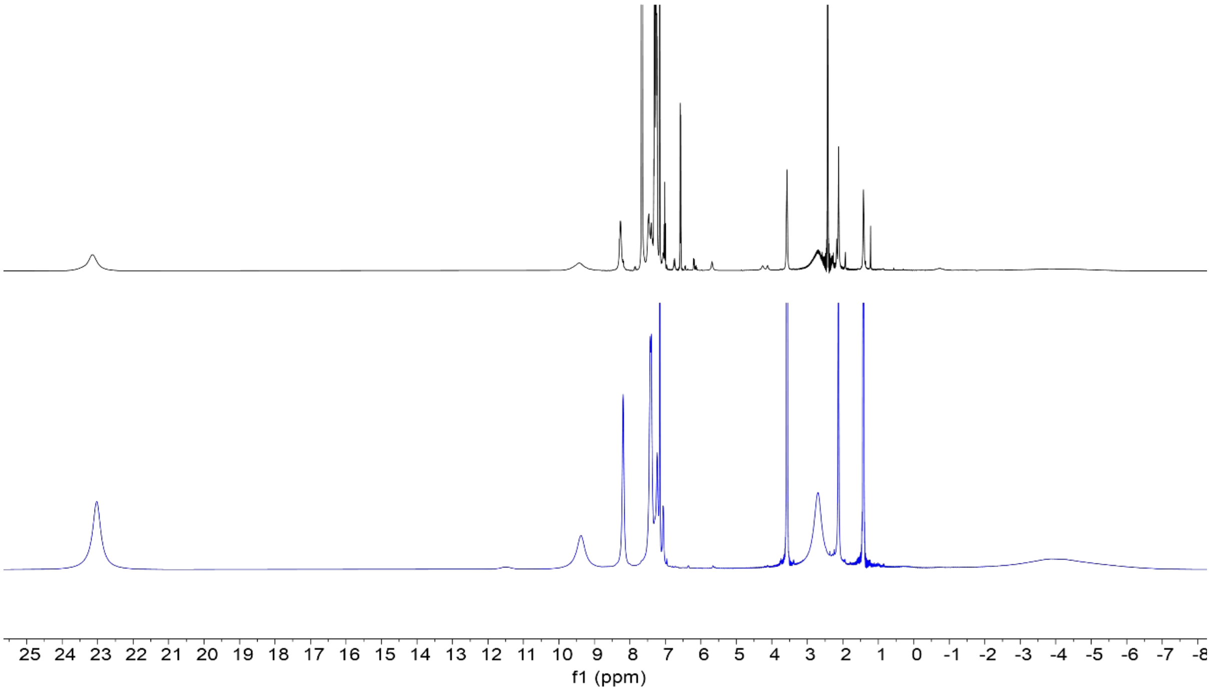

Figure S23. ^{1}H NMR spectra (400 MHz, C_6D_6) of the crude reaction between **4** and 50 mM [Hlut][BPh_4], 50 mM lutidine after 1 h of stirring (top, black) and the isolated product **7** (bottom, blue)

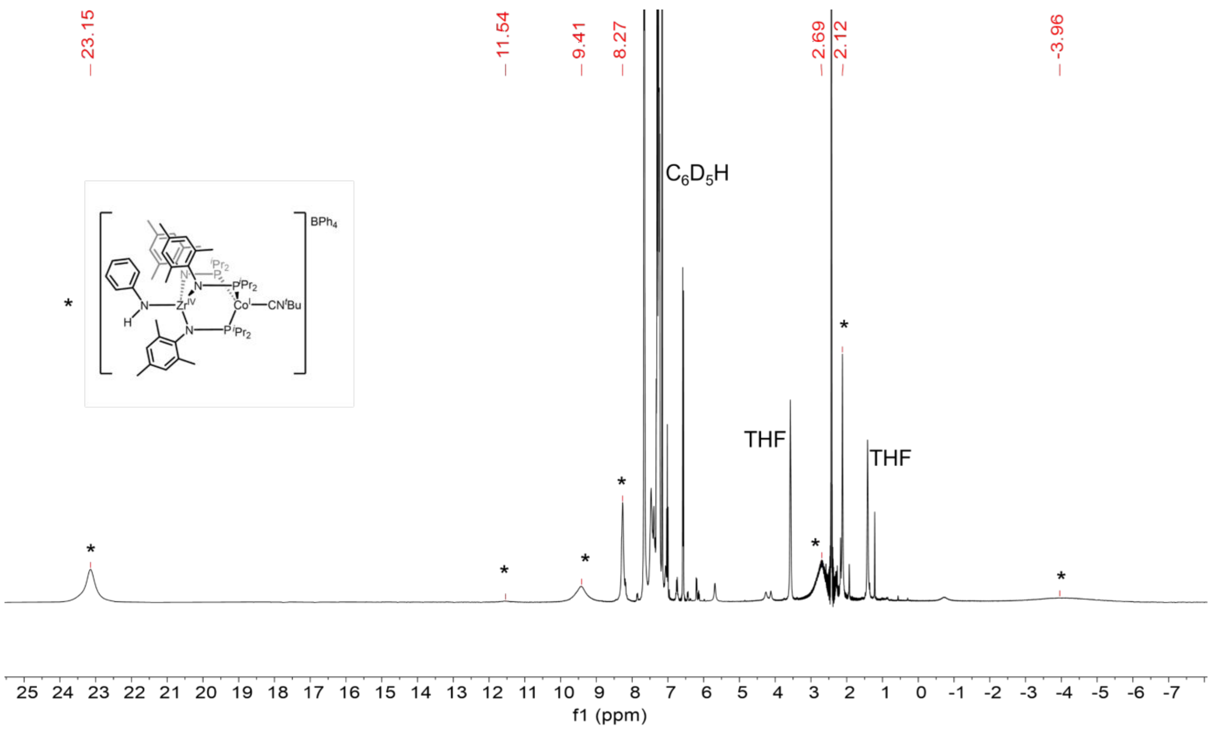

Figure S24. ^{1}H NMR spectra (400 MHz, C_6D_6) of the crude reaction between **4** and 50 mM [Hlut][BPh_4], 50 mM lutidine after 1 h of stirring.

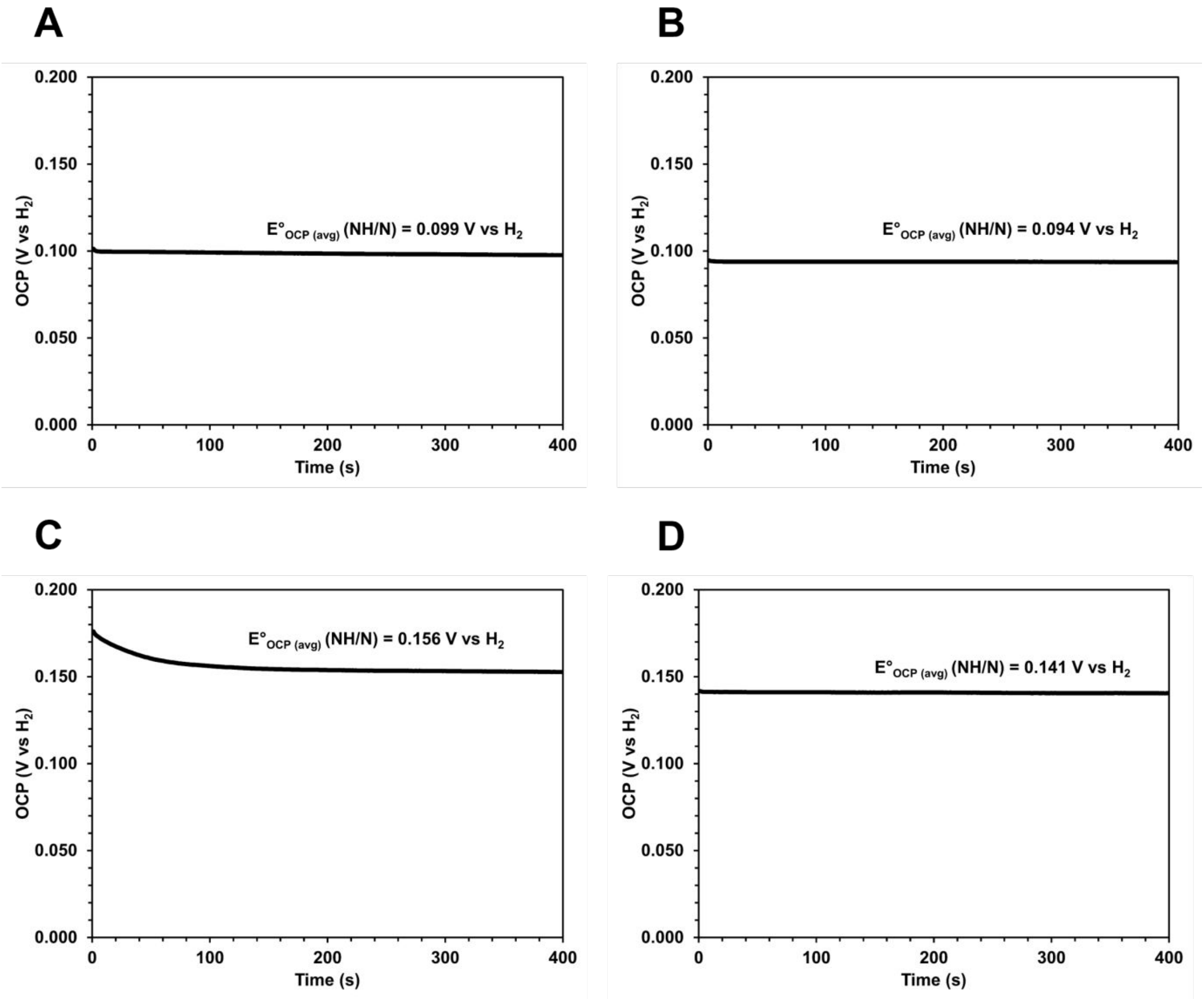

Figure S25. Trial 1: OCP (V vs H_2) over time of THF solutions containing 100 mM [^{n}Bu$_4$N][PF$_6$], 50 mM NEt_3, 50 mM of [HNEt$_3$][BPh$_4$], and varying ratios of **3** and **4**. The OCP was measured every 1 s for 400 s. Each ratio is represented as follows: A) 0.98:1.48, B) 1.31:1.48, C) 1.81:1.48, D) 1.97:1.48.

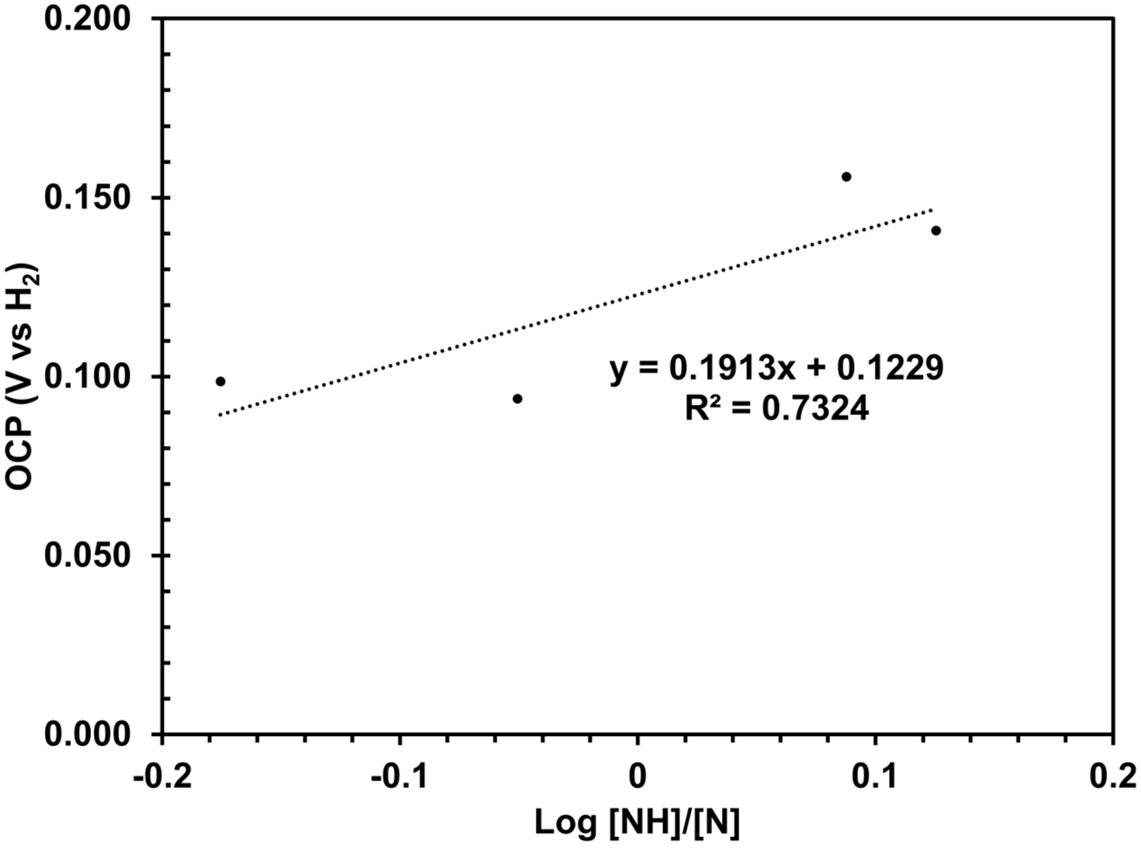

Figure S26. Trial 1: OCP (V vs H_2) vs. the log of varying ratios of complexes **3** and **4**. OCPs were determined by using the $E°_{OCP(avg)}$ (NH/N) values at each given ratio from Figure S25.

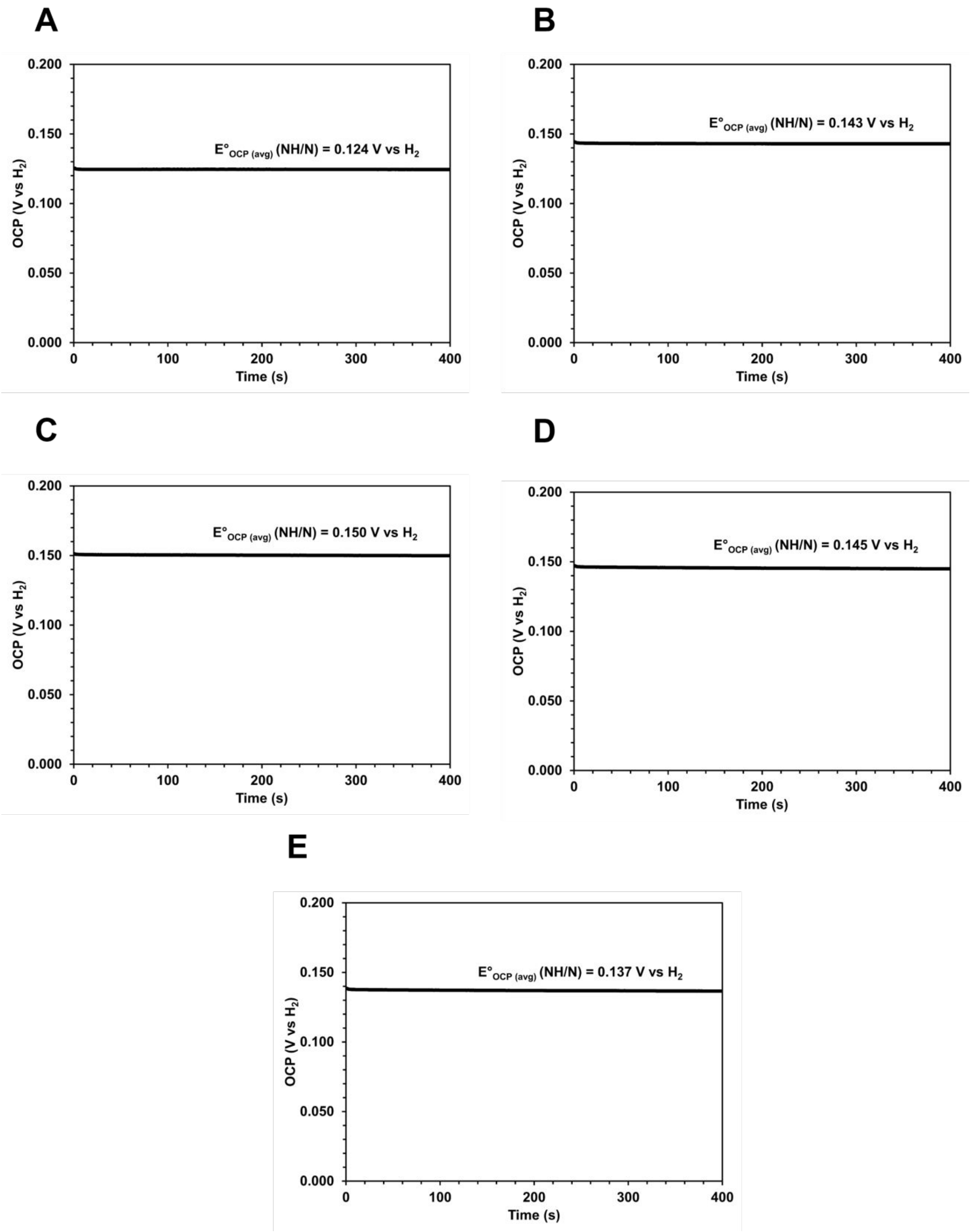

Figure S27. Trial 2: OCP (V vs H_2) over time of THF solutions containing 100 mM [^{n}Bu$_4$N][PF$_6$], 50 mM NEt_3, 50 mM of [$HNEt_3$][BPh_4], and varying ratios of **3** and **4**. The OCP was measured every 1 s for 400 s. Each ratio is represented as follows: A) 0.98:1.48, B) 1.31:1.48, C) 1.48:1.48, D) 1.81:1.48, and E) 1.97:1.48.

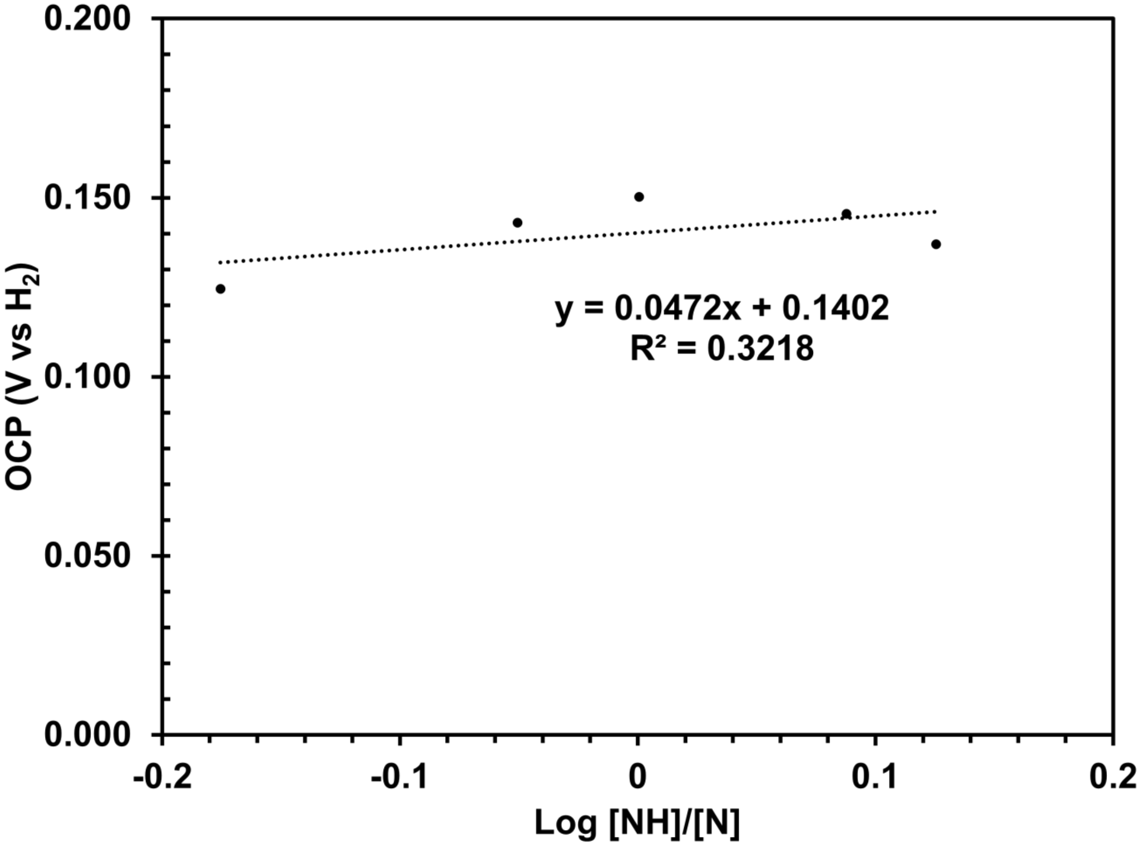

Figure S28. Trial 2: OCP (V vs H_2) vs. the log of varying ratios of complexes **3** and **4**. OCPs were determined by using the $E°_{OCP(avg)}$ (NH/N) values at each given ratio from Figure S27.

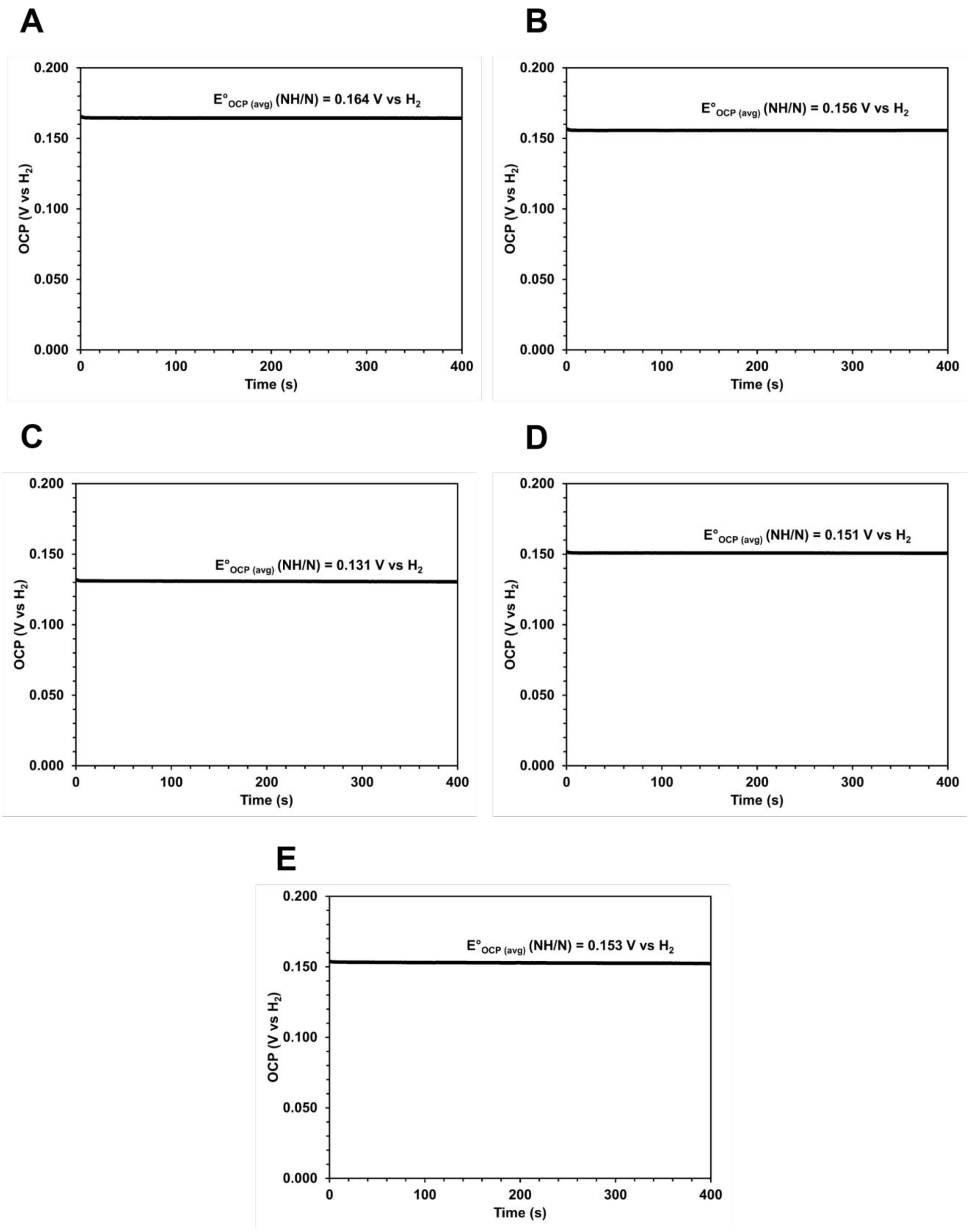

Figure S29. Trial 3: OCP (V vs H_2) over time of THF solutions containing 100 mM [^{n}Bu$_4$N][PF$_6$], 50 mM NEt$_3$, 50 mM of [HNEt$_3$][BPh$_4$], and varying ratios of **3** and **4**. The OCP was measured every 1 s for 400 s. Each ratio is represented as follows: A) 0.98:1.48, B) 1.31:1.48, C) 1.48:1.48, D) 1.81:1.48, and E) 1.97:1.48.

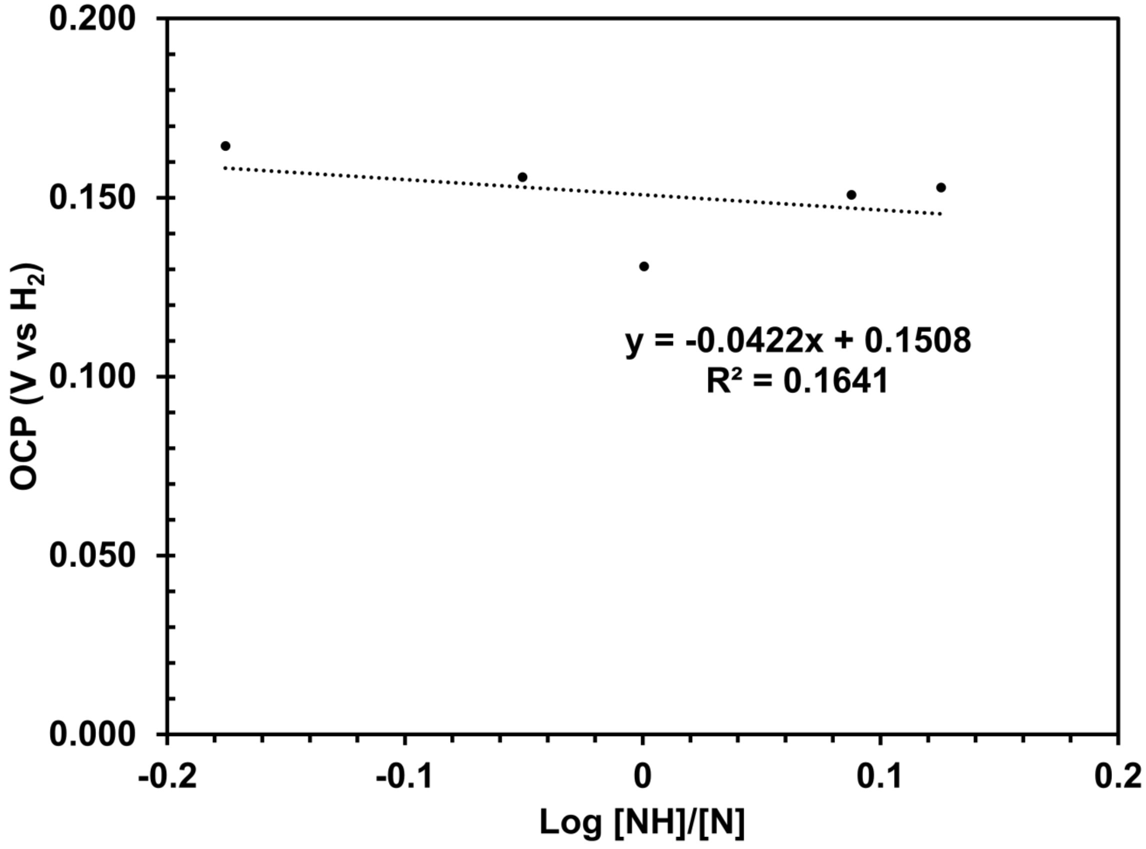

Figure S30. Trial 3: OCP (V vs H_2) vs. the log of varying ratios of complexes **3** and **4**. OCPs were determined by using the $E°_{OCP(avg)}$ (NH/N) values at each given ratio from Figure S29.

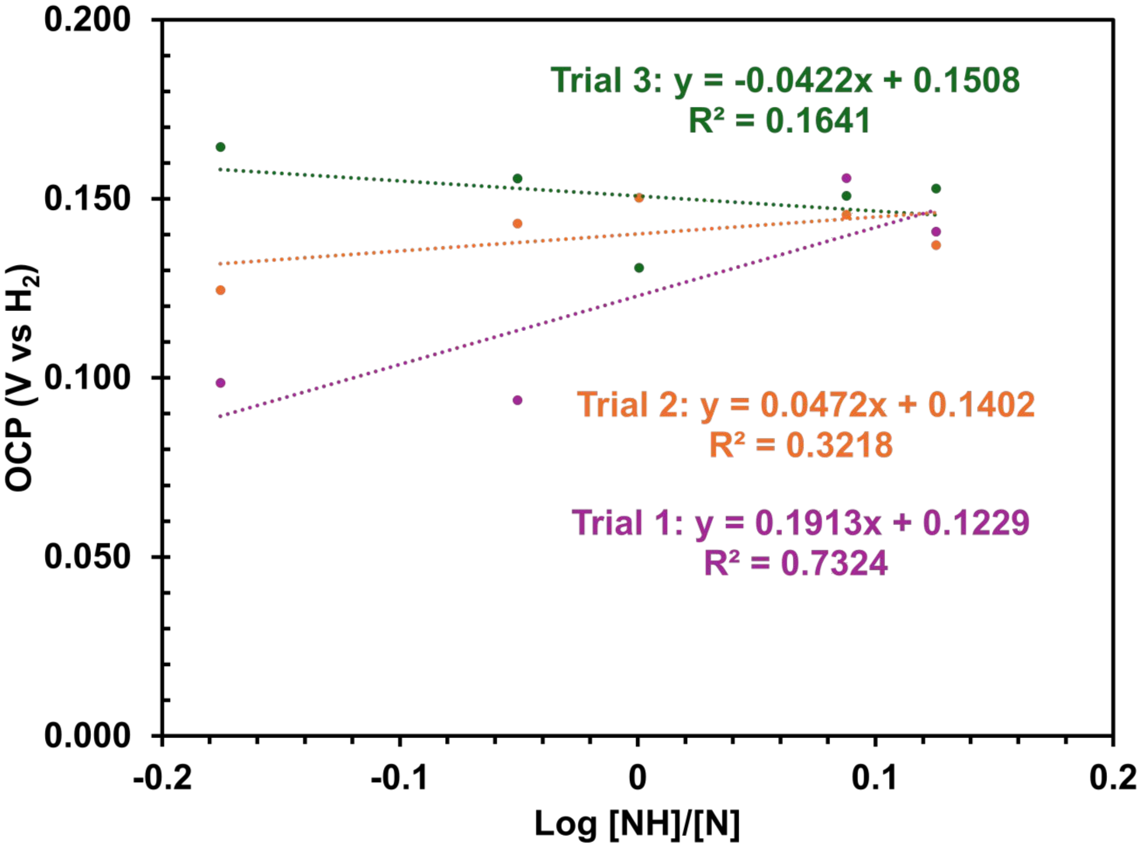

Figure S31. Summary of the OCP (V vs H_2) over the log of varying ratios of complexes **3** and **4**. OCP were determined by using the $E°_{OCP(avg)}$ (NH/N) values at each given ratio from Figure S25, Figure S27, Figure S29.

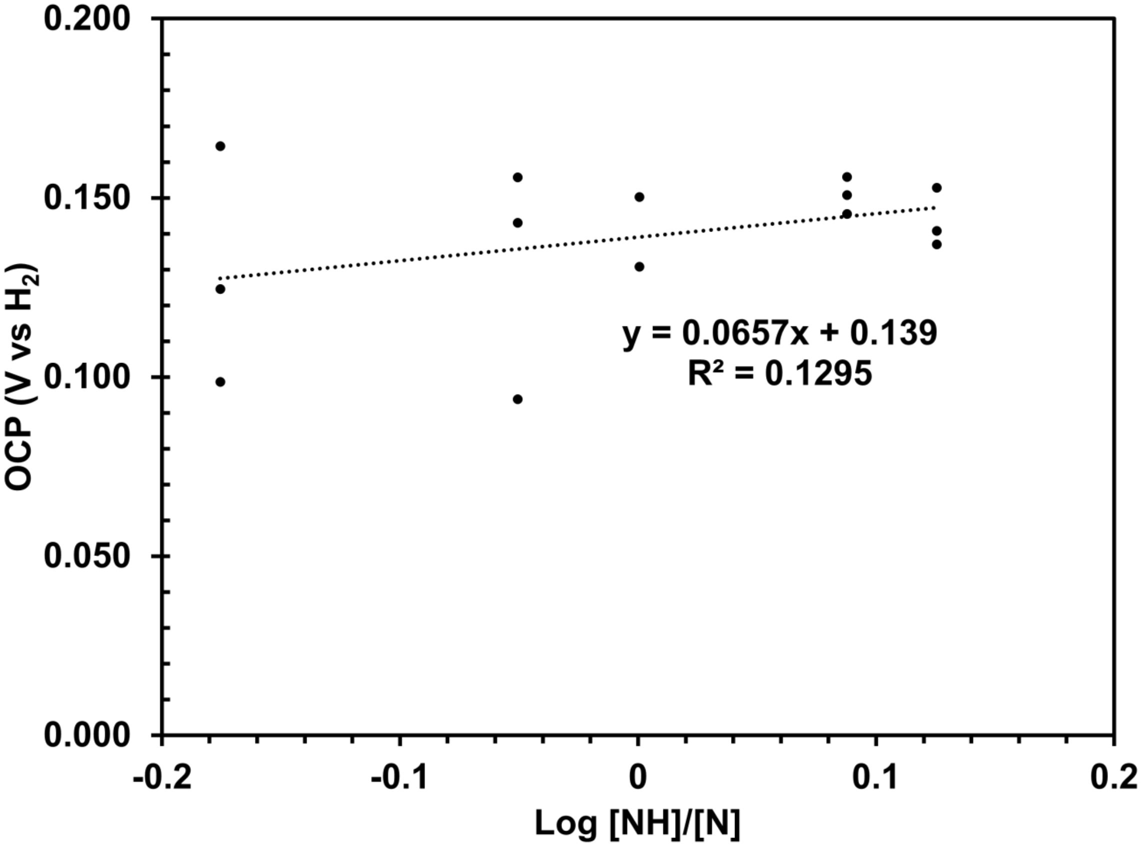

Figure S32. Data points for all three trials combined into a single plot of the OCP (V vs H_2) vs the log of varying ratios of complexes **3** and **4**. OCPs were determined by using the $E°_{OCP(avg)}$ (NH/N) values at each given ratio from Figure S25, Figure S27, and Figure S29.

2.3. Cyclic voltammetry of $(PhH_2N)Zr^{IV}(MesNP^iPr_2)_3Co^{-I}CN^tBu$ (2)

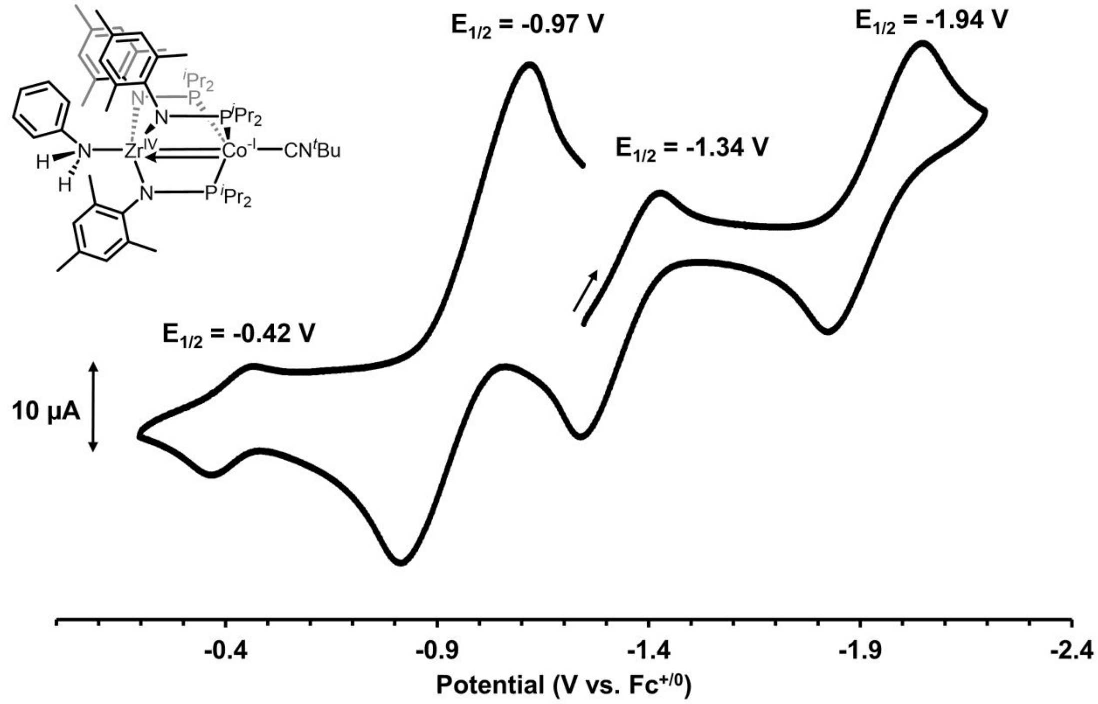

Figure S33. Cyclic voltammogram of **2** vs. $Fc^{+/0}$ collected in 0.1 M $[^nBu_4N][PF_6]$ THF solution, scanning cathodically starting from -1.25 V with a scan rate of 100 mV/s.

2.4. Cyclic voltammetry of (PhHN)Zr^{IV}(MesNP^iPr_2)$_3$$Co^0$CN^{t}Bu (3)

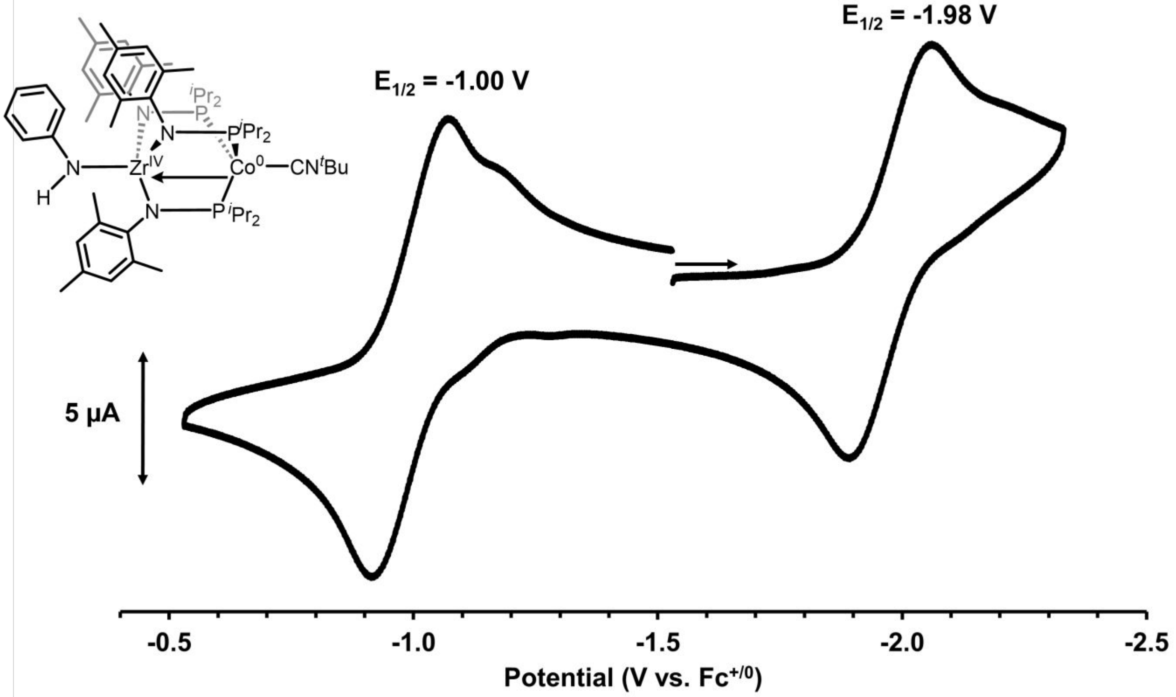

Figure S34. Cyclic voltammogram of **3** vs. $Fc^{+/0}$ collected in 0.1 M [^{n}Bu_4N][PF_6] THF solution, scanning cathodically starting from -1.60 V with a scan rate of 100 mV/s.

2.5. Cyclic voltammetry of (PhN)Zr^{IV}(MesNP^{i}Pr$_2$)$_3$Co^{I}CN^{t}Bu (4)

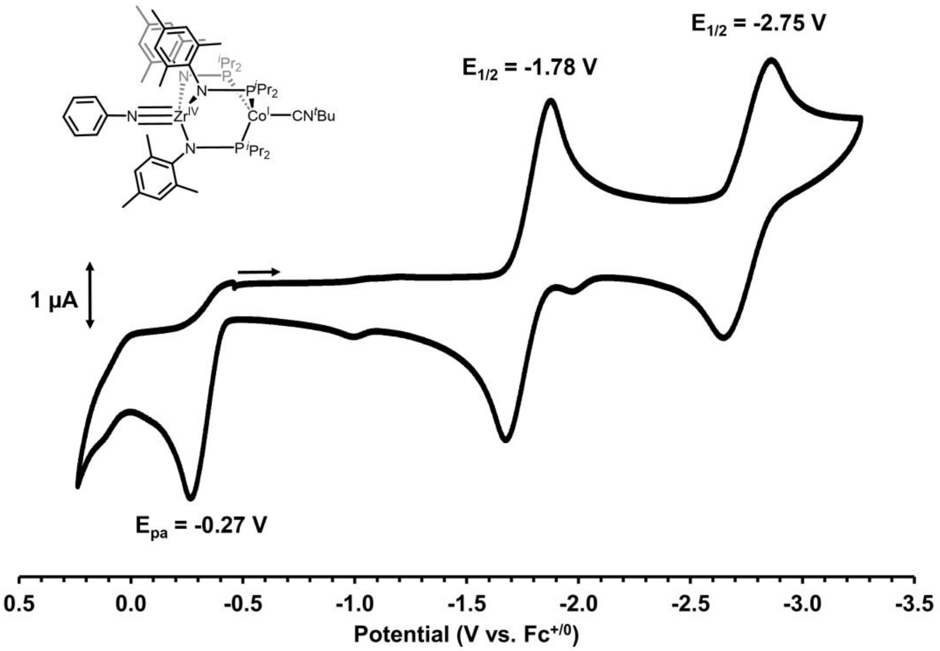

Figure S35. Cyclic voltammogram of **4** vs. $Fc^{+/0}$ collected in 0.1 M [$^{n}Bu_4N$][PF_6] THF solution, scanning cathodically starting from the open circuit potential with a scan rate of 100 mV/s.

2.6. Cyclic voltammetry of [PhHNZr^{IV}(MesNP^{i}Pr$_2$)$_3$Co^{I}CN^{t}Bu][BPh$_4$] (7)

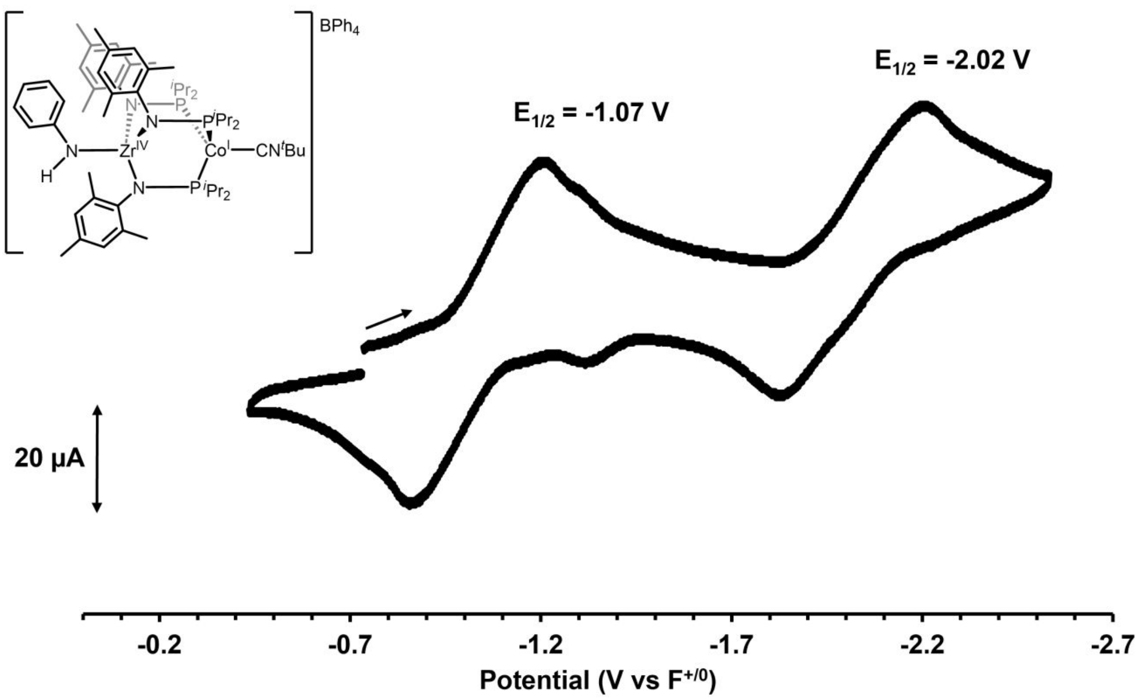

Figure S36. Cyclic voltammogram of **7** vs. Fc$^{+/0}$ collected in 0.1 M [^{n}Bu$_4$N][PF$_6$] THF solution, scanning cathodically starting from the open circuit potential with a scan rate of 300 mV/s.

3. Direct conversion of OCP (V vs H_2) to $BDFE_{N–H}$

$$X + nHA + ne^- \xrightleftharpoons{E^{\circ\prime}_{OCP}\ (\text{V vs Fc}^+/\text{Fc})} XH_n + nA^-$$

$$(^n/_2)H_2(g) + nA^- \xrightleftharpoons{-E^{\circ\prime}\ (\text{H}^+/\text{H}_2,\ \text{V vs Fc}^+/\text{Fc})} nHA + ne^-$$

$$X + (^n/_2)H_2(g) \xrightleftharpoons{E^{\circ\prime}\ (\text{X/XH}_n,\ \text{V vs H}_2)} XH_n$$

Scheme S1. Direct conversion of OCP (V vs H_2) to $BDFE_{X-H}$. Reprinted (adapted) with permission from Wise, C. F.; Agarwal, R. G.; Mayer, J. M.; *J. Am. Chem. Soc.*, **2020**, *142*, 10681-10691. Copyright 2020 American Chemical Society.

$$\mathrm{BDFE(X-H)} = 23.06\mathrm{E}^\circ(\mathrm{X/XH\ V\ vs\ H_2}) + \Delta\mathrm{G}^\circ(\tfrac{1}{2}\mathrm{H_2(g)/H^\bullet{}_{1M}} \quad \text{(eq. S1)}$$

$$\mathrm{BDFEsol(X-H)} = 1.37\mathrm{p}K_\mathrm{a} + 23.06\mathrm{E}^\circ(X^{0/-}) + \mathrm{C}_{G,sol} \quad \text{(eq. S2)}$$

The X/XH OCP referenced to the OCP of the H^+/H_2 solution, provides a direct route (Scheme S1) to calculate the $BDFE_{X-H}$ by substituting the OCP of X/XH vs H^+/H_2 (E° (V vs H_2)) in eq #.[2] The $\Delta G^\circ(\frac{1}{2}H_2\ (g)/H^\bullet{}_{1M})$ is a solvent-dependent value. In THF, $\Delta G^\circ(\frac{1}{2}H_2\ (g)/H^\bullet{}_{1M})$ is 52.0 kcal/mol.[2] With the $BDFE_{X-H}$ value in hand, the pK_a of metal complexes can be estimated with the Bordwell equation (eq. S2).[3,4] To employ the Bordwell equation, redox potentials of metal complexes determined by CV data (SI, Section 2.3-2.5) and the solvent-specific constant ($C_{g,sol}$ = 59.9 kcal/mol, in THF)[5] were used.

3.1. $BDFE_{N–H}$ sample calculation of first H atom abstraction between complexes $(PhH_2N)Zr^{IV}(MesNP^{i}Pr_2)_3Co^{-I}CN^{t}Bu$ (2) to $(PhHN)Zr^{IV}(MesNP^{i}Pr_2)_3Co^{0}CN^{t}Bu$ (3)

$$\mathrm{BDFE(N-H)} = 23.06(-0.650) + \Delta G°(52)$$

Where -0.650 is the average y-intercept that corresponds to a 1:1 ratio between complexes **2** and **3** (Figure S20)

$$\mathrm{BDFE(N-H)} = 37.0\ \mathrm{kcal/mol}$$

$$\mathrm{BDFE(N-H)} = 37\ \pm 1\ \mathrm{kcal/mol}$$

3.2. $BDFE_{N–H}$ sample calculation of second H atom abstraction between complexes $(PhH)NZr^{IV}(MesNP^{i}Pr_2)_3Co^{0}CN^{t}Bu$ (3) to $(PhN)Zr^{IV}(MesNP^{i}Pr_2)_3Co^{I}CN^{t}Bu$ (4)

$$\mathrm{BDFE(N-H)} = 23.06(0.139) + \Delta G°(52)$$

Where 0.138 is the average y-intercept that corresponds to a 1:1 ratio between complexes **3** and **4** (Figure S31)

$$\mathrm{BDFE(N-H)} = 55.2\ \mathrm{kcal/mol}$$

$$\mathrm{BDFE(N-H)} = 55\ \pm 1\ \mathrm{kcal/mol}$$

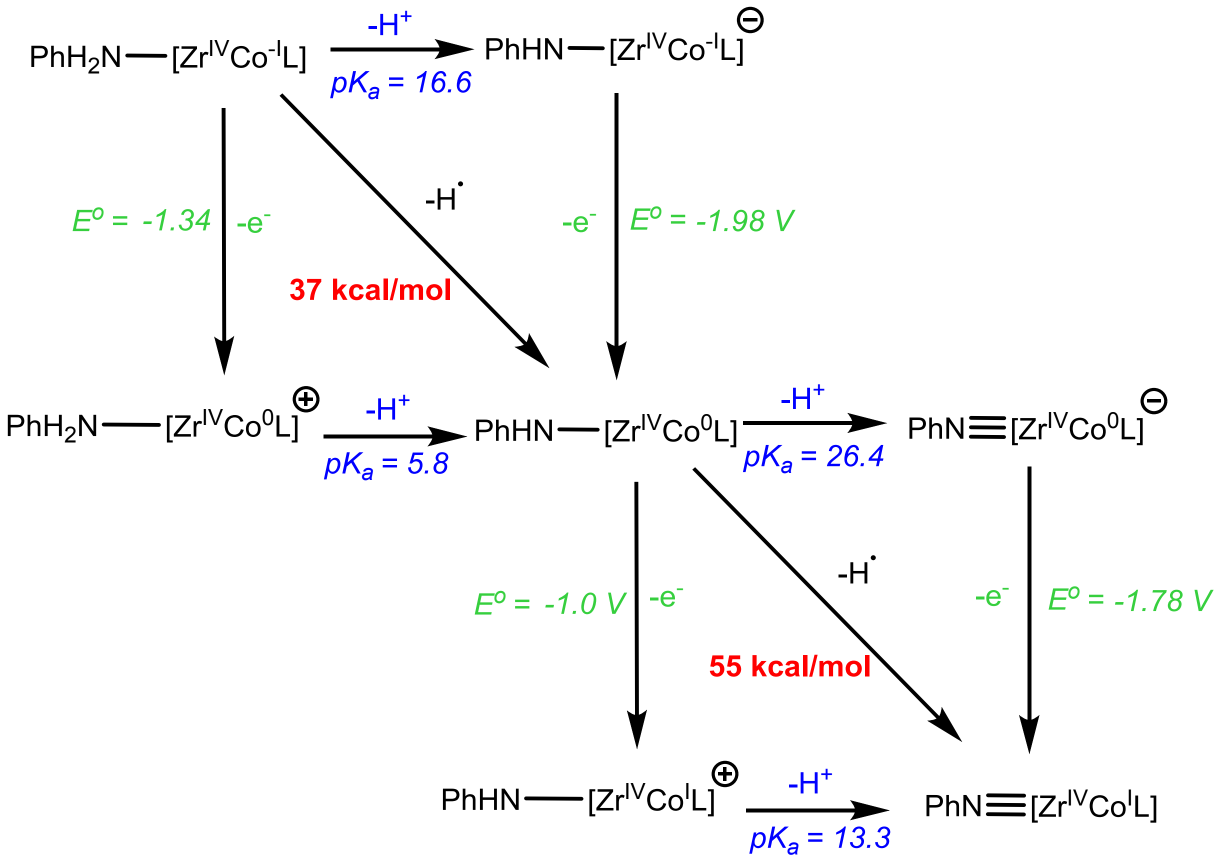

Scheme S2. Thermochemical data illustrated by a square scheme. Values in green were measured using CV; values in in red were determined using OCP measurements; values in blue were calculated using the potential and BDFE values using the Bordwell equation.

3.3. pK_a sample calculation

$$\mathrm{BDFEsol(X-H)} = 1.37\mathrm{p}K_\mathrm{a} + 23.06\mathrm{E}°(X^{0/-}) + \mathrm{C_{G,sol}} \quad \text{(eq. S2)}$$

$$\mathrm{p}K_\mathrm{a} = \frac{37 - 59.9 - 23.06(-1.98)}{1.37} = 16.6$$

$$\mathrm{p}K_\mathrm{a} = \frac{37 - 59.9 - 23.06(-1.34)}{1.37} = 5.8$$

$$\mathrm{p}K_\mathrm{a} = \frac{40 - 59.9 - 23.06(-1.78)}{1.37} = 26.4$$

$$\mathrm{p}K_\mathrm{a} = \frac{40 - 59.9 - 23.06(-1.00)}{1.37} = 13.3$$

3.4. Uncertainty in $BDFE_{N–H}$

The uncertainty in $BDFE_{N–H}$ was estimated using the observed deviation in both OCP experiments, the E_{OCP} (H^+/H_2) vs $Fc^{+/0}$ and the E_{OCP} (NH_2/NH) vs $Fc^{+/0}$. A standard deviation of 40.5 mV was observed for the E_{OCP} (H^+/H_2) vs $Fc^{+/0}$ for the 50 mM lutidine/ 50 mM [Hlut][BPh_4] buffer system as previously reported.[1] A standard deviation of 22.4 mV was observed for E_{OCP} (NH_2/NH) vs $Fc^{+/0}$ measurements. Taking these deviations as additive, a total of 62.9 mV corresponds to a BDFE uncertainty of 1.4 kcal/mol when multiplied by the 23.06 kcal/mol conversion factor. Additionally, if the y-intercept of each trial is used to calculate the BDFE (following the steps demonstrated in the sample calculation in section 3.1.) a range of values from 36.6 kcal/mol to 37.6 kcal/mol is computed, which is in agreement with the 1.4 kcal/mol uncertainty. Trial 1: y-intercept of -0.6238, BDFE = 37.6 kcal/mol, trial 2: y-intercept of -0.6519, BDFE = 37.0 kcal/mol, trial 3: y-intercept of -0.668, BDFE of 36.6 kcal/mol.

The same uncertainty analysis was done for the E_{OCP} (N/NH) vs $Fc^{+/0}$ measurements, in which a standard deviation of 11.2 mV was observed for E_{OCP} (NH/N) vs $Fc^{+/0}$ measurements. A standard deviation of 7 mV was observed for the E_{OCP} (H^+/H_2) vs $Fc^{+/0}$ for the 50 mM NEt_3/ 50 mM [$HNEt_3$][BPh_4] buffer system previously reported by Mayer. [2] Taking the 11.2 mV and the 7 mV as additive, a total of 18.2 mV corresponds to a BDFE uncertainty of 0.4 kcal/mol. Using the y-intercept of each trial a range of BDFE values of 54.8 kcal/mol to 55.5 kcal/mol is observed. Trial 1: y-intercept of 0.1229, BDFE = 54.8 kcal/mol, trial 2: y-intercept of 0.1402, BDFE = 55.2 kcal/mol, trial 3: y-intercept of 0.1508, BDFE of 55.5 kcal/mol.

The estimated uncertainty is similar to the uncertainty values we previously reported[1] and to other reported BDFE uncertainty values of 1.0[2] and 1.3[6] kcal/mol, which also include temperature differences and electrode drift over multiple days. The OCP measurements reported herein were collected on the same day using the same electrodes to minimize temperature differences and electrode drift.

4. $BDFE_{N–H}$ test reactions

4.1. $(PhH_2N)Zr^{IV}(MesNP^iPr_2)_3Co^{-I}CN^tBu$ (2) with 1,8-dichloroanthraquinone

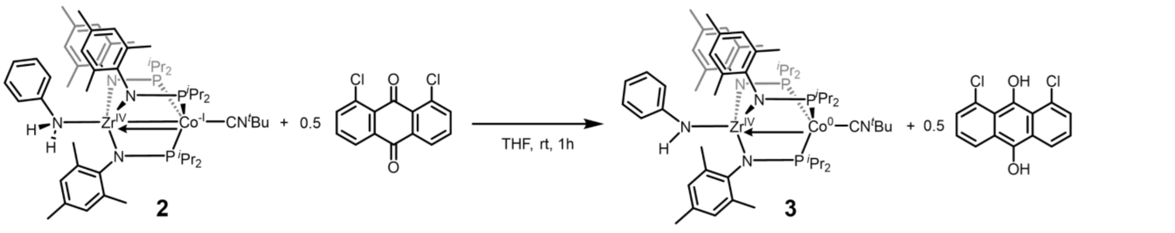

BDFE = 37.0 kcal/mol

BDFE = 56.3 kcal/mol

Complex **2** (12.8 mg, 0.0119 mmol) was dissolved in THF (~2 mL) and added to 1,8-dichloroanthraquinone (1.6 mg, 0.0058 mmol) with stirring. The solution became a deep red/purple color. The reaction mixture was allowed to stir at room temperature for 1 h. The THF was removed under vacuum prior to ^1H NMR analysis

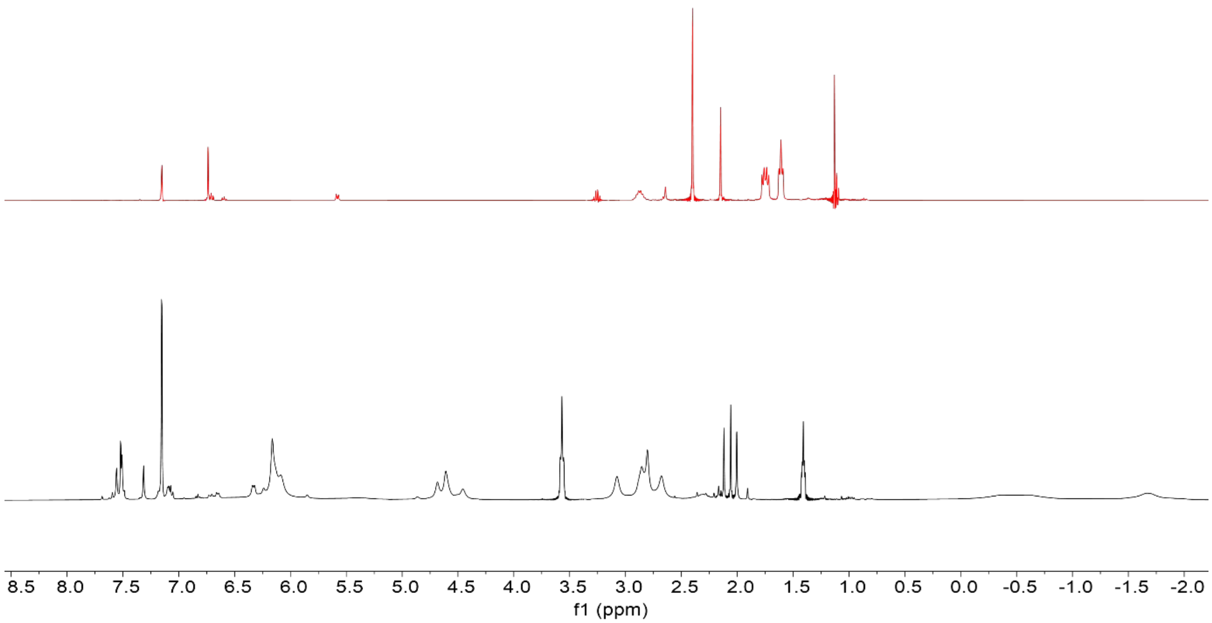

Figure S37. ^{1}H NMR spectra (400 MHz, C_6D_6) of **2** (top, red) and the crude reaction between **2** and 1,8-dichloroanthraquinone after 1 h of stirring (bottom, black).

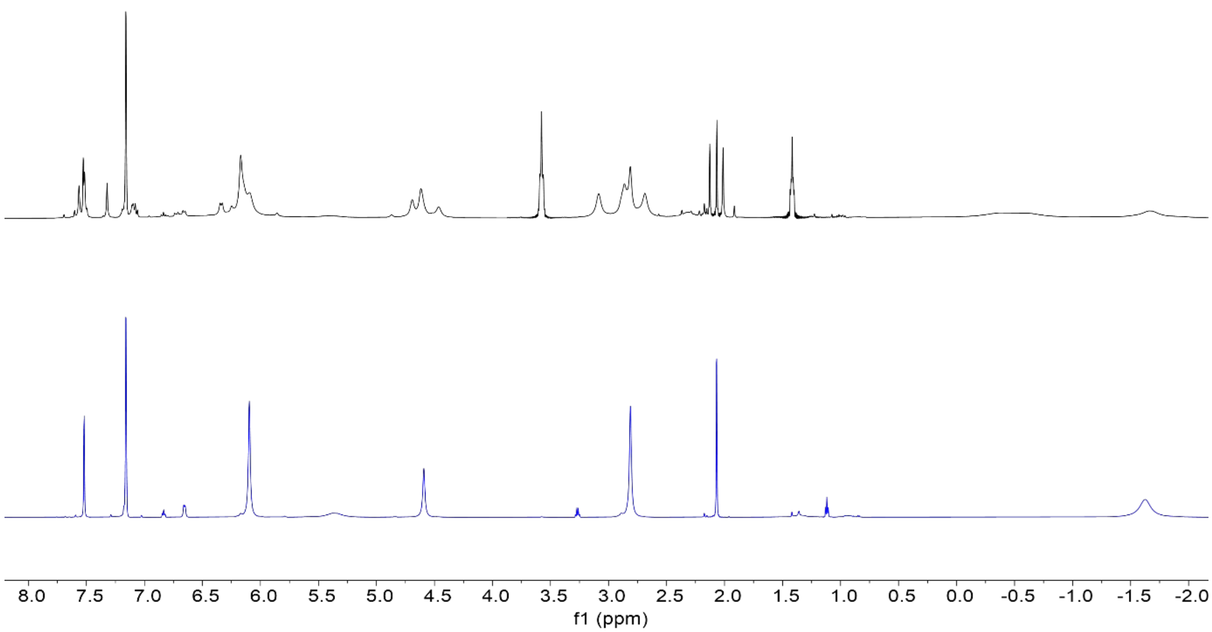

Figure S38. ^{1}H NMR spectra (400 MHz, C_6D_6) of the crude reaction between **2** and 1,8-dichloroanthraquinone after 1 h of stirring (top, black) and the isolated desired product **3** (bottom, blue).

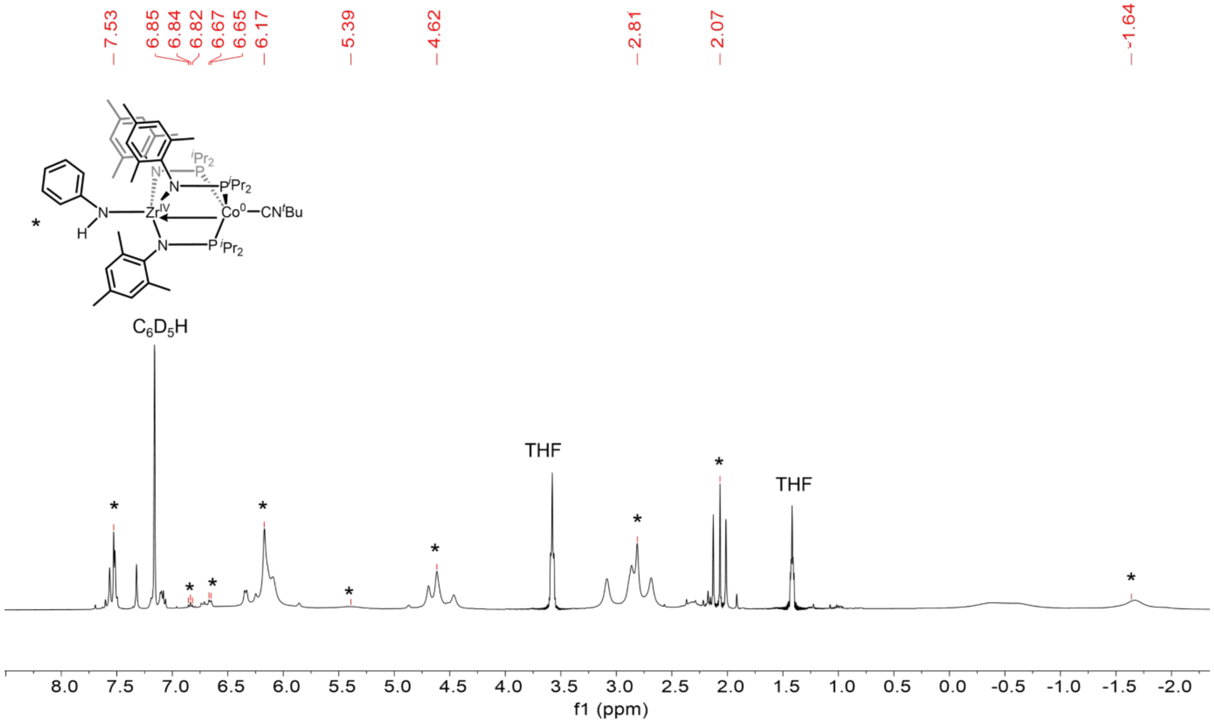

Figure S39. ^{1}H NMR spectra (400 MHz, C_6D_6) of the crude reaction between **2** and 1,8-dichloroanthraquinone after 1 h of stirring.

4.2. (PhH)NZr^{IV}(MesNP^{i}Pr$_2$)$_3$Co^{0}CN^{t}Bu (3) with 1,8-dichloroanthraquinone

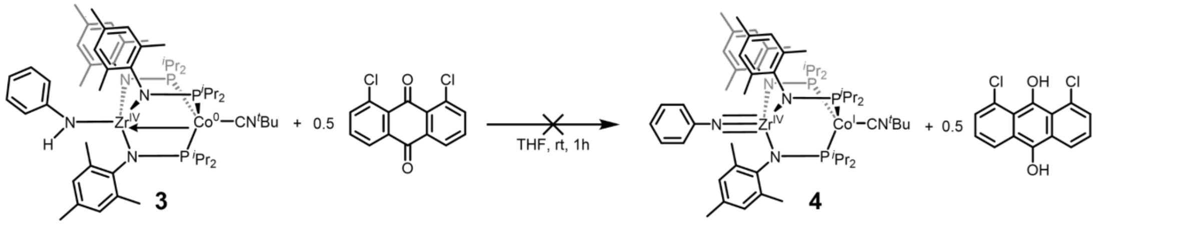

3 4

BDFE = 55.2 kcal/mol BDFE = 56.3 kcal/mol

Complex **3** (10.4 mg, 0.00788 mmol) was dissolved in THF (~2 mL) and added to 1,8-dichloroanthraquinone (1.4 mg, 0.0051 mmol) with stirring. The solution became a deep red color. The reaction mixture was allowed to stir at room temperature for 1 h. The THF was removed under vacuum prior to ^{1}H NMR analysis. Although the expected outcome was reactivity to generate **4**, the resulting ^{1}H NMR spectrum revealed no formation of **4**. This can be attributed to the ~1 kcal/mol difference between the BDFE values between complex **3** and the 1,8-dichloroanthraquinone reagent, allowing the reaction to reach and stay at an equilibrium based on the observed broadening of the ^{1}H NMR peaks. Adding an additional 0.5 equiv of 1,8-dichloroanthraquinone did not result in appreciable formation of **4**.

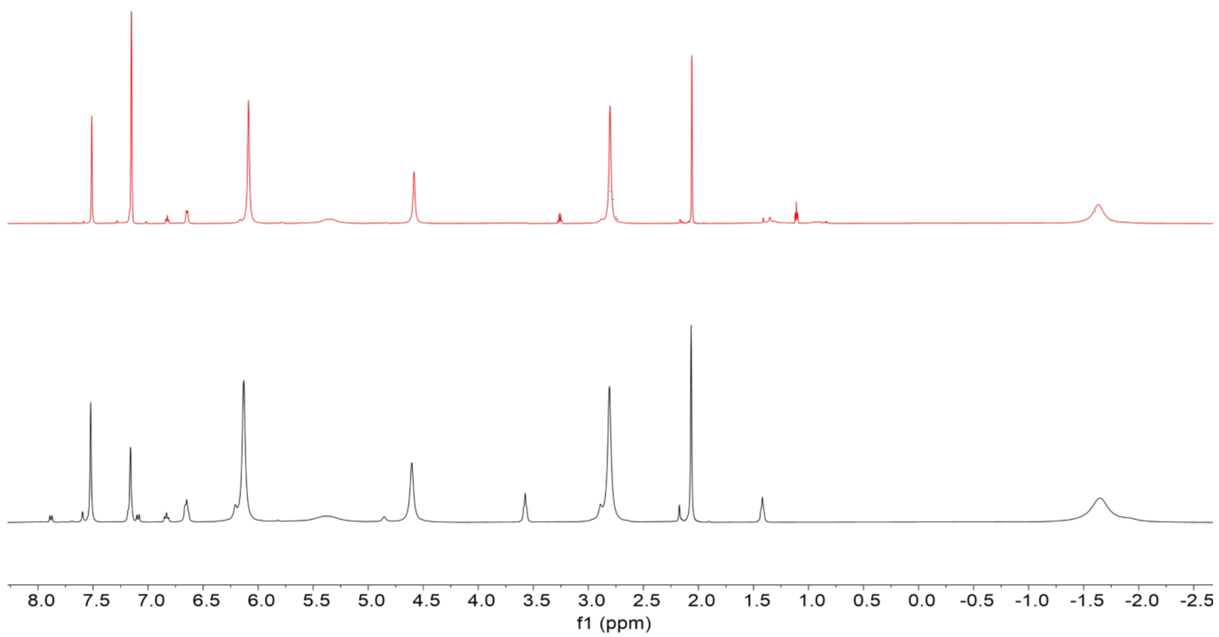

Figure S40. ^{1}H NMR spectra (400 MHz, C_6D_6) of **3** (top, red) and the crude reaction between **3** and 1,8-dichloroanthraquinone after 1 h of stirring (bottom, black).

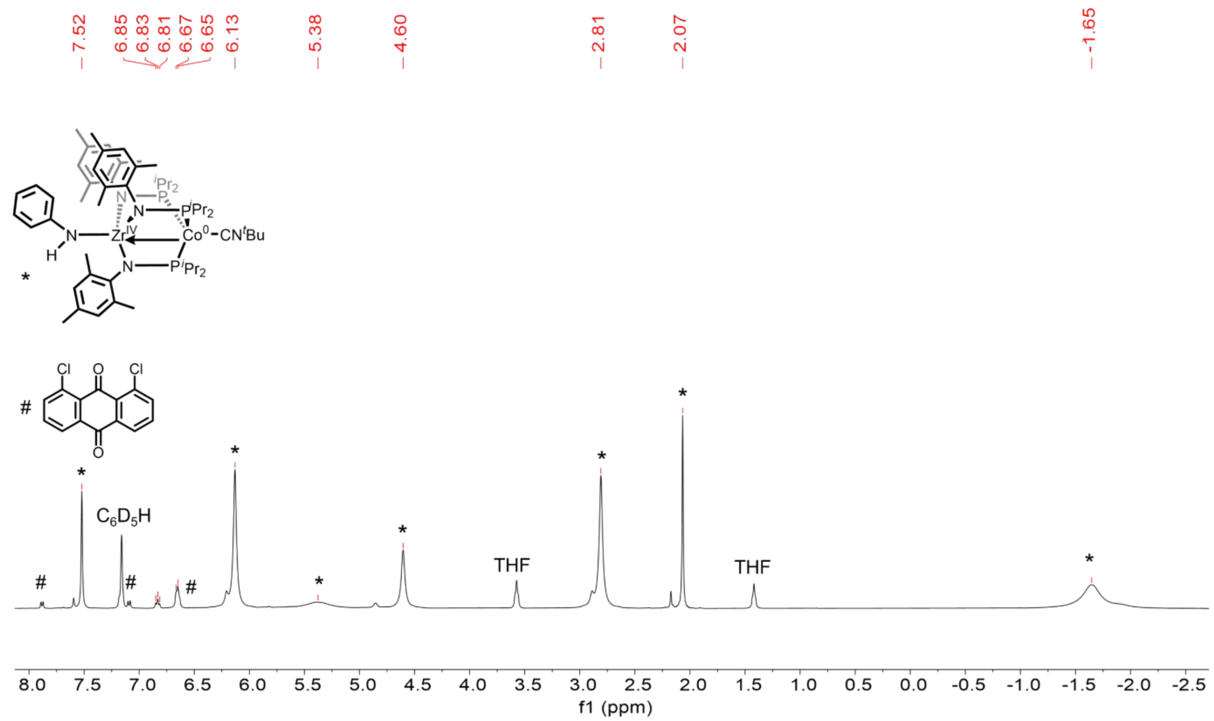

Figure S41. ^{1}H NMR spectra (400 MHz, C_6D_6) of the crude reaction between **3** and 1,8-dichloroanthraquinone after 1 h of stirring.

4.3. (PhH)NZr^{IV}(MesNP^{i}Pr$_2$)$_3$Co^{0}CN^{t}Bu (3) with *p*-benzoquinone

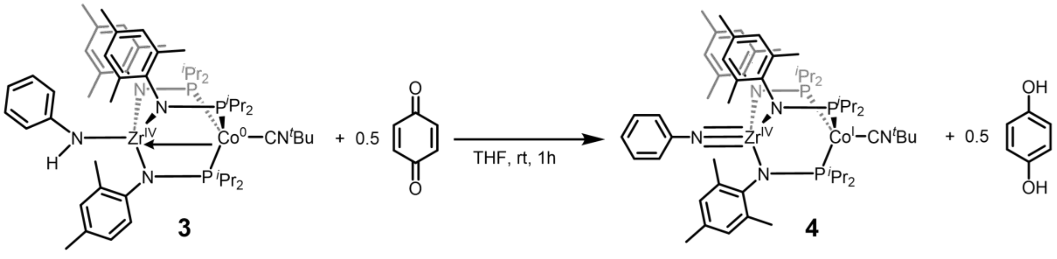

3 **4**

BDFE = 55.2 kcal/mol BDFE = 67.4 kcal/mol

Complex **3** (20.6 mg, 0.0191 mmol) was dissolved in THF (~2 mL) and added to *p*-benzoquinone (1.3 mg, 0.012 mmol) with stirring. A color change from orange-red to green was observed with a green solid precipitating out of solution. The reaction mixture was allowed to stir at room temperature for 1 h. The THF was removed under vacuum prior to ^{1}H NMR analysis.

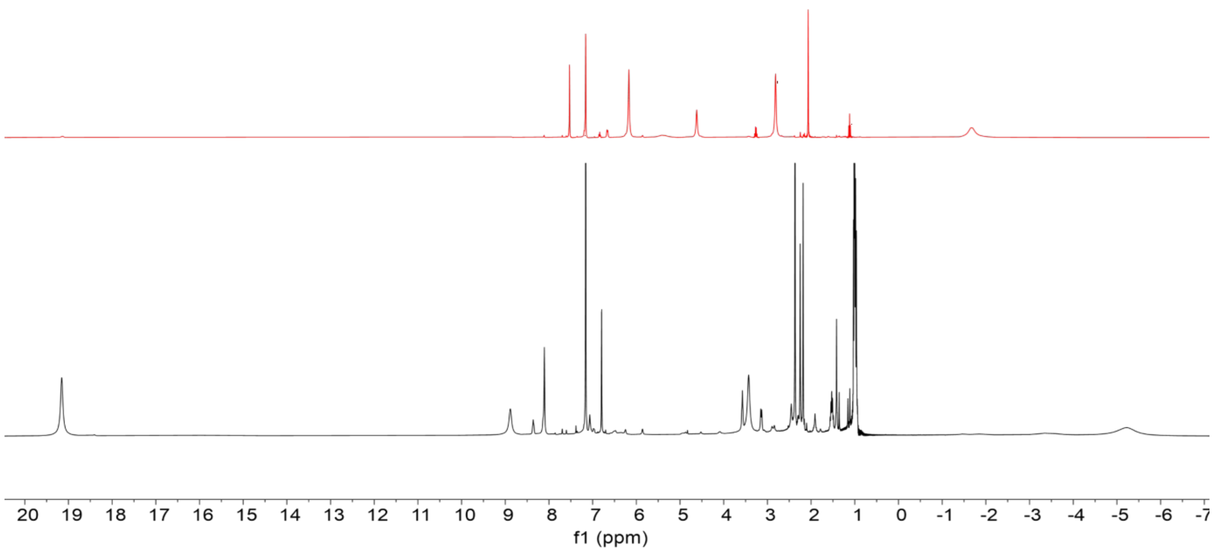

Figure S42. ^{1}H NMR spectra (400 MHz, C_6D_6) of **3** (top, red) and the crude reaction between **3** and *p*-benzoquinone after 1 h of stirring (bottom, black).

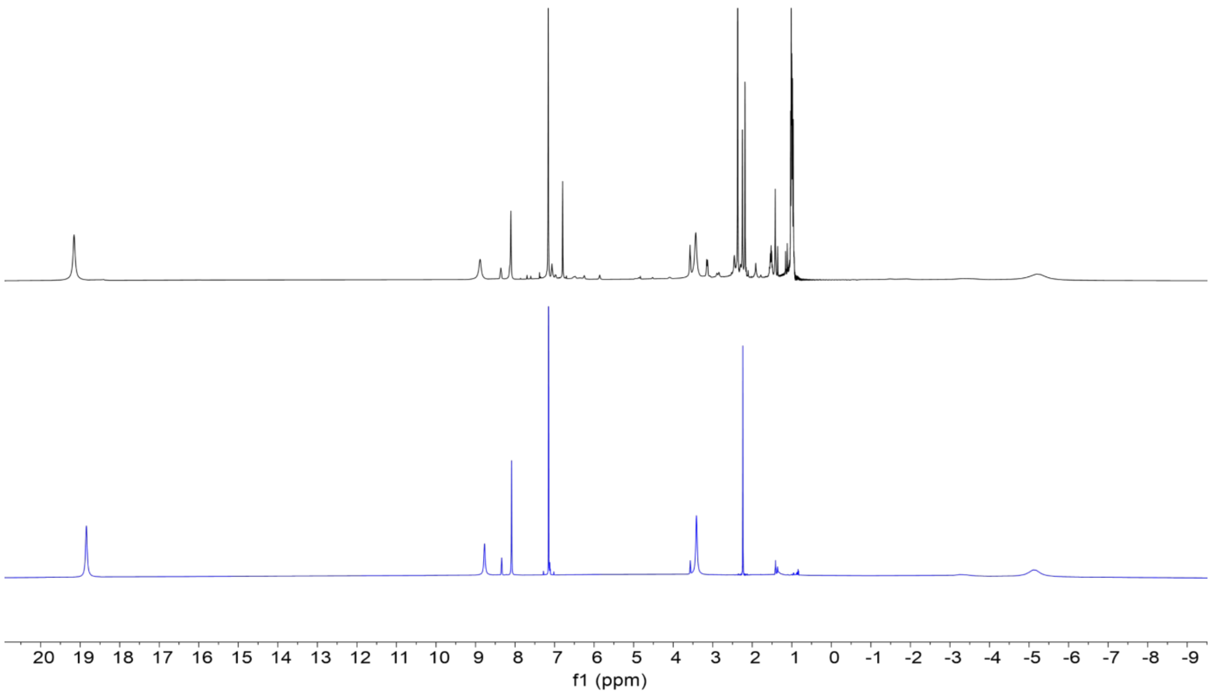

Figure S43. ^{1}H NMR spectra (600 MHz, C_6D_6) of the crude reaction between **3** and *p*-benzoquinone after 1 h of stirring (top, black) and the isolated desired product **4** (bottom, blue).

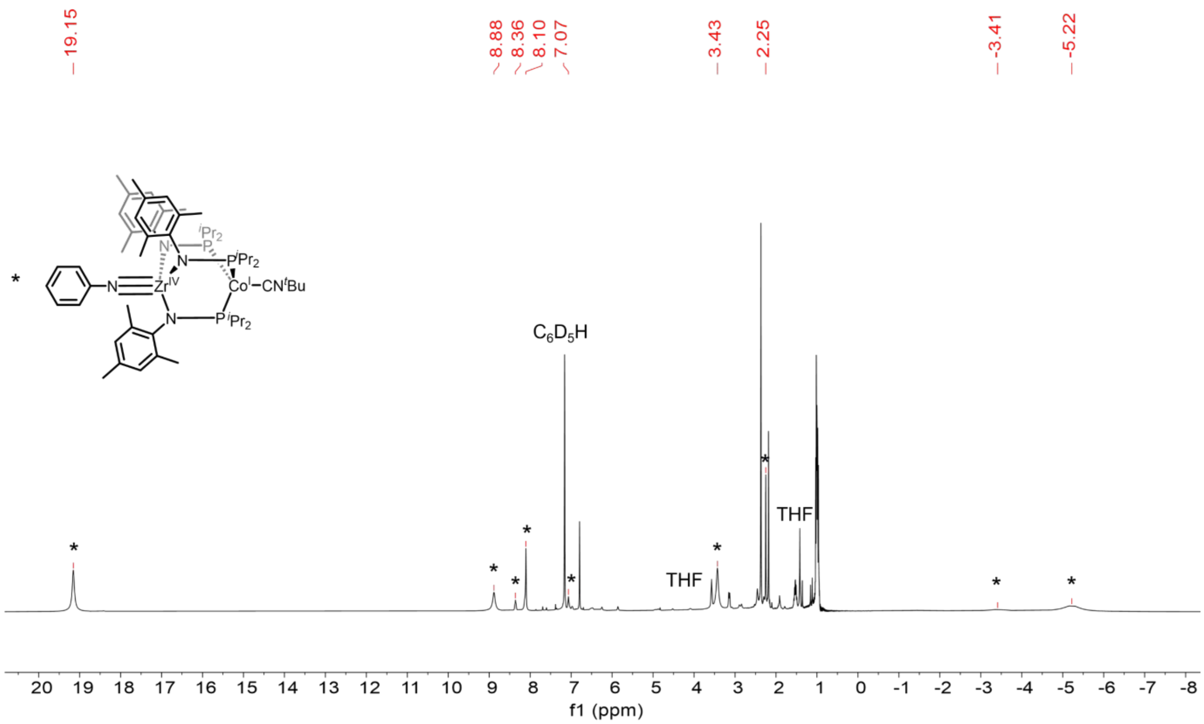

Figure S44. ^{1}H NMR spectra (400 MHz, C_6D_6) of the crude reaction between **3** and *p*-benzoquinone after 1 h of stirring.

5. pK_a test reaction

pK_a (PhH$_2$N)Zr^{IV}(MesNP^{i}Pr$_2$)$_3$Co^{-I}CN^{t}Bu (2) = 16.6

5.1. Reaction of (PhH$_2$N)Zr^{IV}(MesNP^{i}Pr$_2$)$_3$Co^{-I}CN^{t}Bu (2) with LiN(SiMe$_3$)$_2$

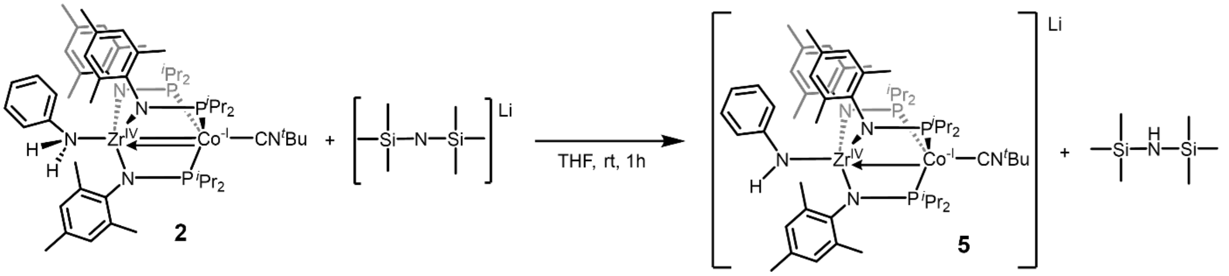

pK_a = 16.6 pK_a = 25.8

LiN(SiMe$_3$)$_2$ (1.8 mg, 0.011 mmol) was dissolved in THF (~2 mL) and added to **2** (10.8 mg, 0.0100 mmol) with stirring. No notable color change was observed; the solution remained red. The reaction mixture was allowed to stir at room temperature for 1 hour. The THF was removed under vacuum prior to ^{1}H NMR analysis. The resulting ^{1}H NMR spectrum revealed complete conversion to **5**.

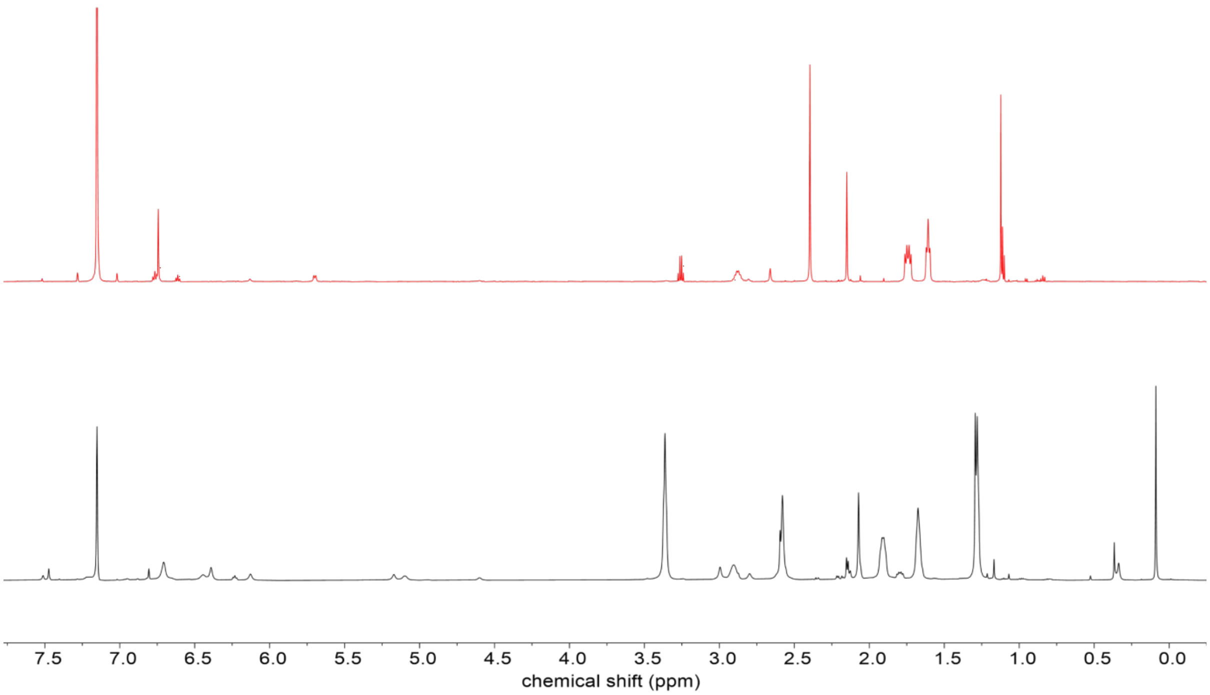

Figure S45. ^1H NMR spectra (600 MHz, C_6D_6) of **2** (top, red) and the crude reaction between **2** and $LiN(SiMe_3)_2$ after 1 h of stirring (bottom, black).

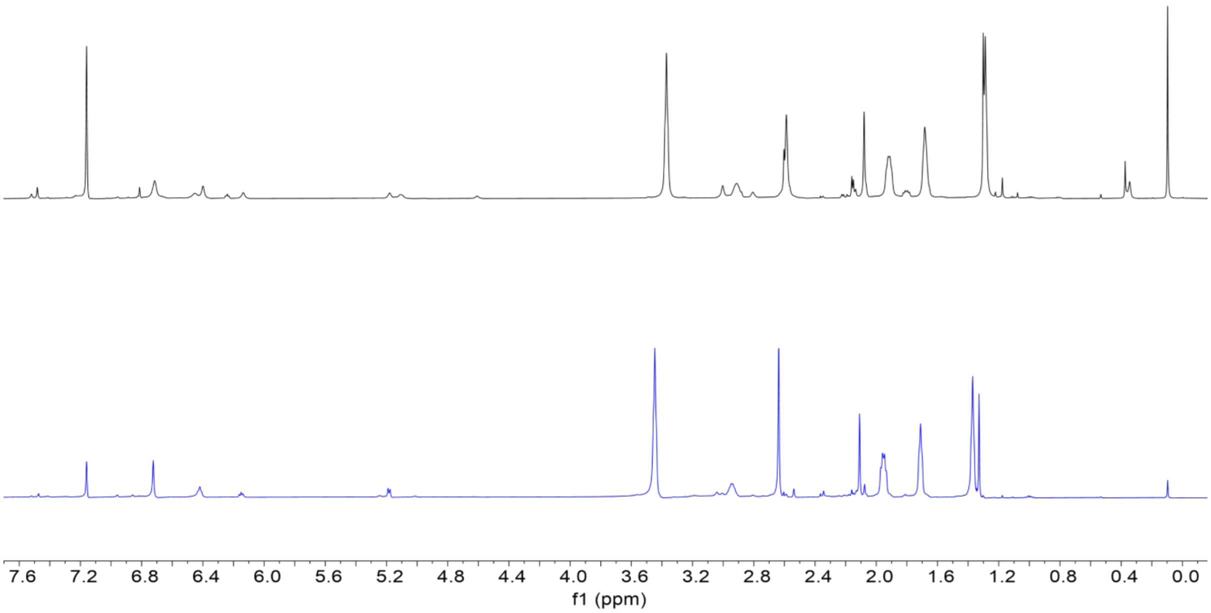

Figure S46. ^{1}H NMR spectra (600 MHz, C_6D_6) of the crude reaction between **2** and $LiN(SiMe_3)_2$ after 1 h of stirring (top, black) and the isolated desired product **5** (bottom, blue).

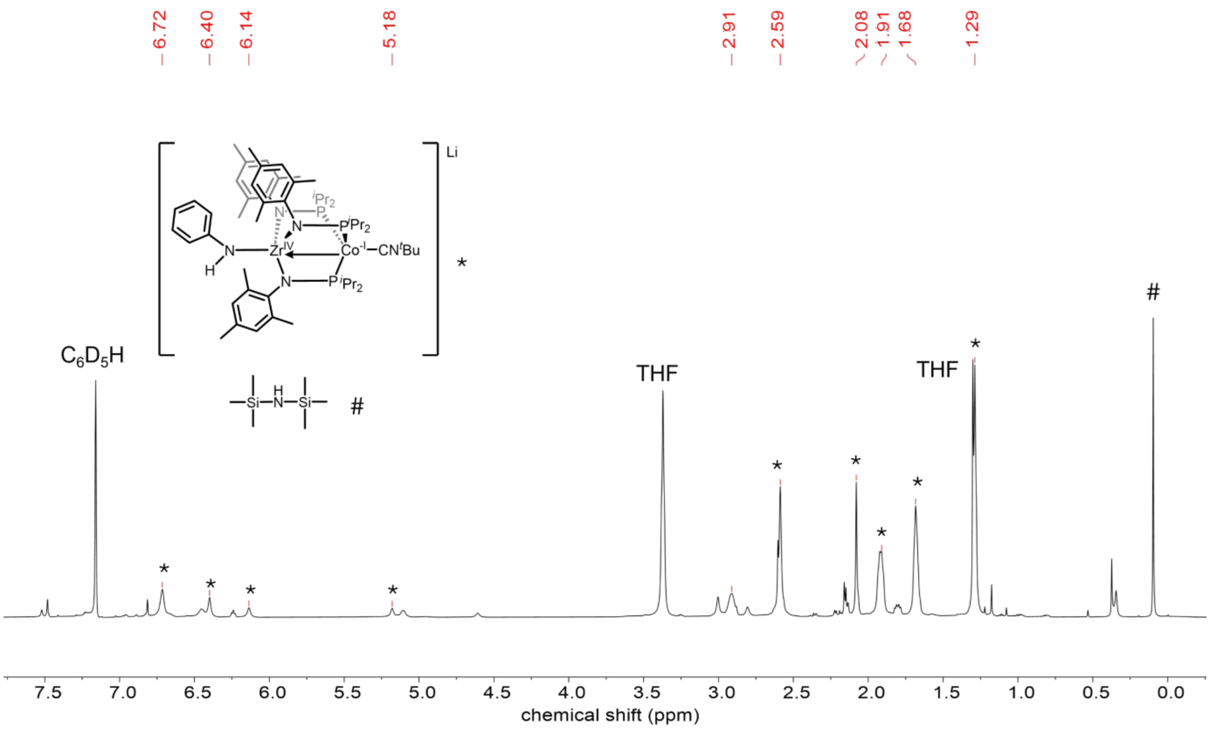

Figure S47. ^{1}H NMR spectrum (600 MHz, C_6D_6) of the crude reaction between **2** and $LiN(SiMe_3)_2$ after 1 h of stirring.

5.2. $(PhH_2N)Zr^{IV}(MesNP^iPr_2)_3Co^{-I}CN^tBu$ (2) with NEt_3

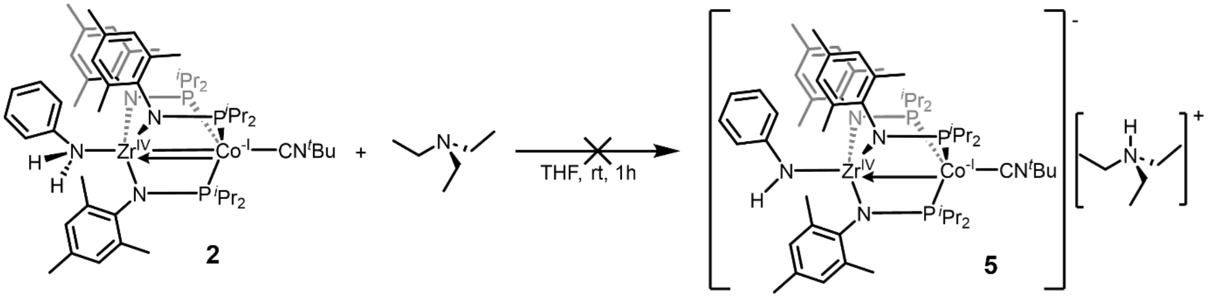

2 pK_a = 16.6

5 pK_a = 12.5

NEt_3 (1.1 mg, 0.011 mmol) was added to a stirring solution of **2** (9.7 mg, 0.0090 mmol) in THF (~2 mL). There was no color change observed during the reaction; the solution remained red. The reaction mixture was allowed to stir at room temperature for 1 h. The volatile components were removed under vacuum prior to ^1H NMR analysis. The resulting ^1H NMR spectrum revealed no reaction.

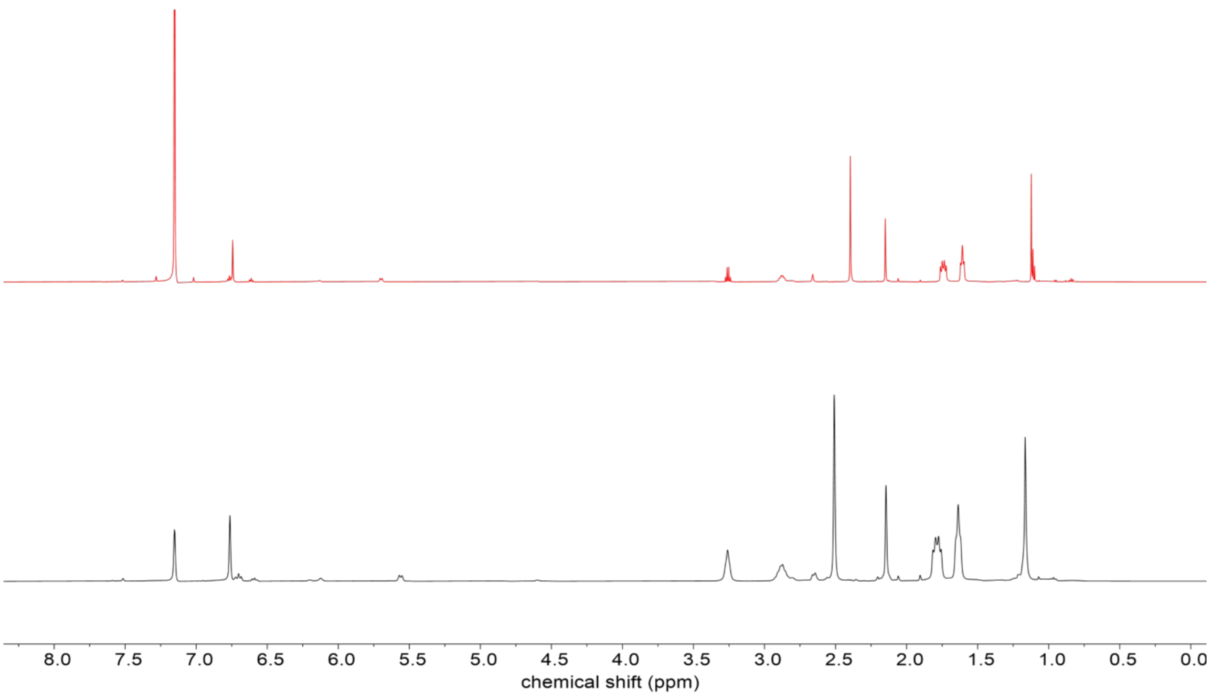

Figure S48. ^1H NMR spectra (400 MHz, C_6D_6) of **2** (top, red) and the crude reaction between **2** and NEt_3 after 1 h of stirring (bottom, black).

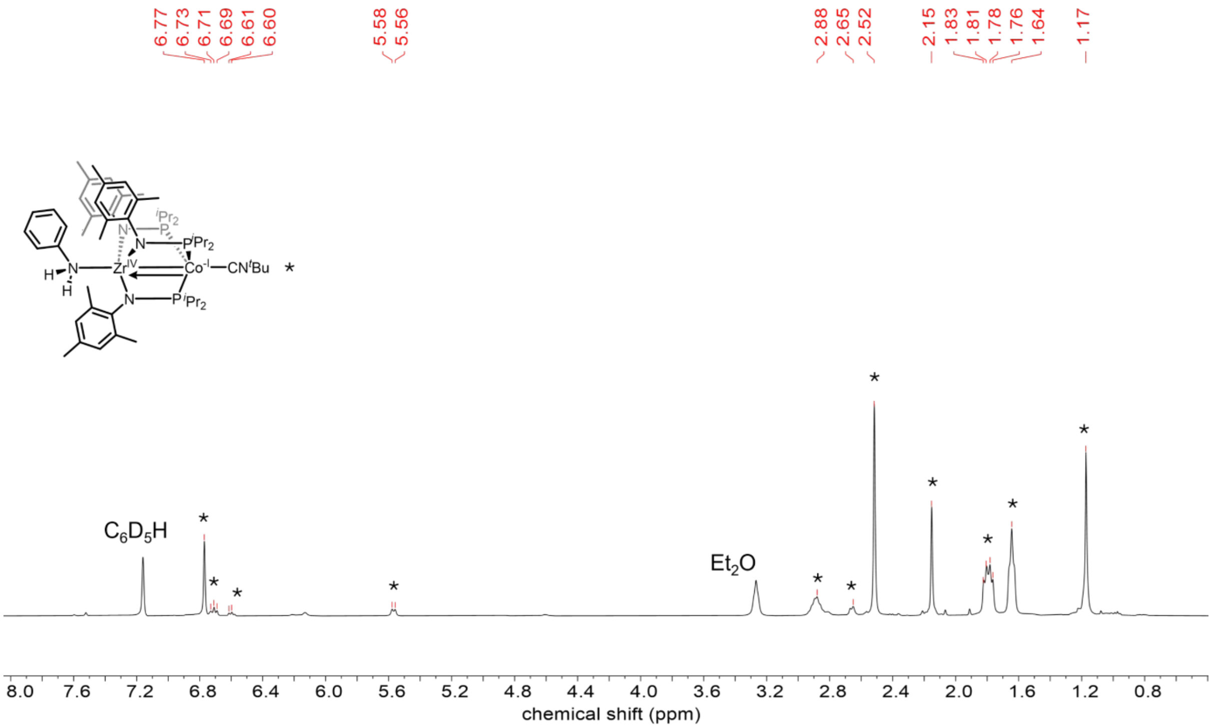

Figure S49. ^{1}H NMR spectrum (400 MHz, C_6D_6) of the crude reaction between **2** and NEt_3 after 1 h of stirring.

pK_a (PhHN)Zr^{IV}(MesNP^{i}Pr$_2$)$_3$Co^{0}CN^{t}Bu (3) = 26.4

5.3. Reaction of (PhHN)Zr^{IV}(MesNP^{i}Pr$_2$)$_3$Co^{0}CN^{t}Bu (3) with LiN(SiMe$_3$)$_2$

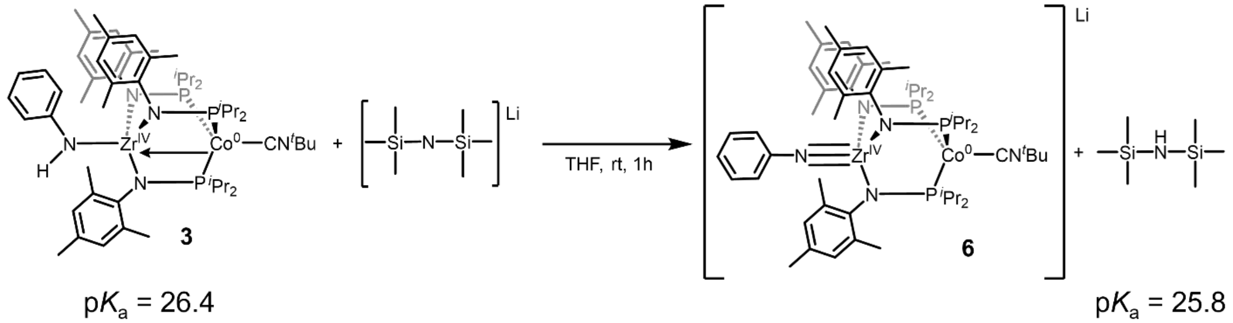

LiN(SiMe$_3$)$_2$ (1.9 mg, 0.011 mmol) was dissolved in THF (~2 mL) and added to **3** (10.1 mg, 0.00938 mmol) with stirring. No notable color change was observed; the solution remained orange. The reaction mixture was allowed to stir at room temperature for 1 hour. The THF was removed under vacuum prior to ^{1}H NMR analysis. The resulting ^{1}H NMR spectrum revealed complete conversion to **6**. Although the expected outcome was no reactivity, the resulting ^{1}H NMR spectrum revealed complete conversion to **6**. The difference observed between the calculated value and the experimental data may be because the calculated pK_a represents the pK_a in a buffered electrolyte solution rather than THF alone.

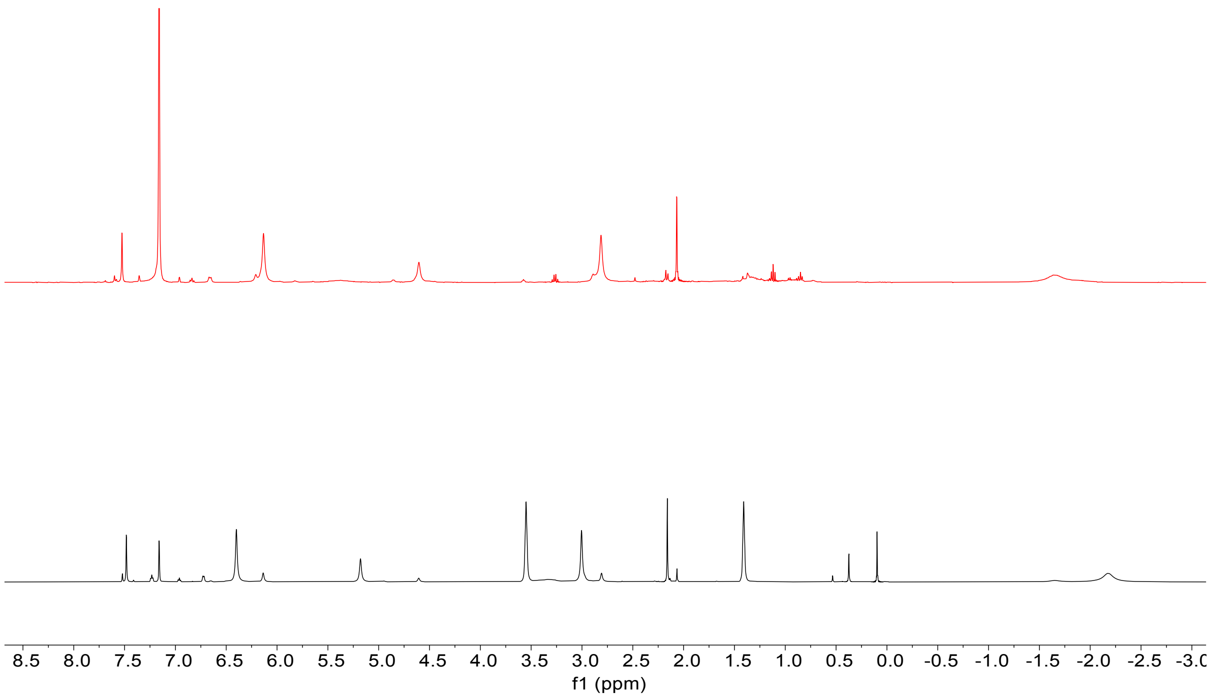

Figure S50. ^{1}H NMR spectra (600 MHz, C_6D_6) of **3** (top, red) and the crude reaction between **3** and $LiN(SiMe_3)_2$ after 1 h of stirring (bottom, black).

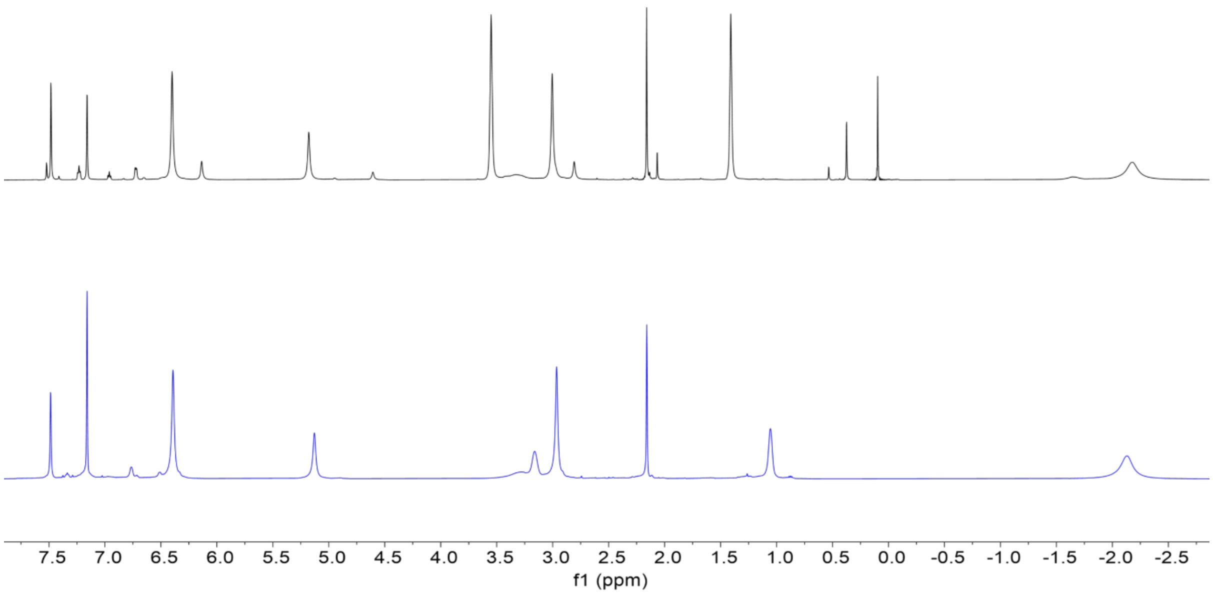

Figure S51. ^{1}H NMR spectra (600 MHz, C_6D_6) of the crude reaction between **3** and $LiN(SiMe_3)_2$ after 1 h of stirring (top, black) and the isolated desired product **6** (bottom, blue).

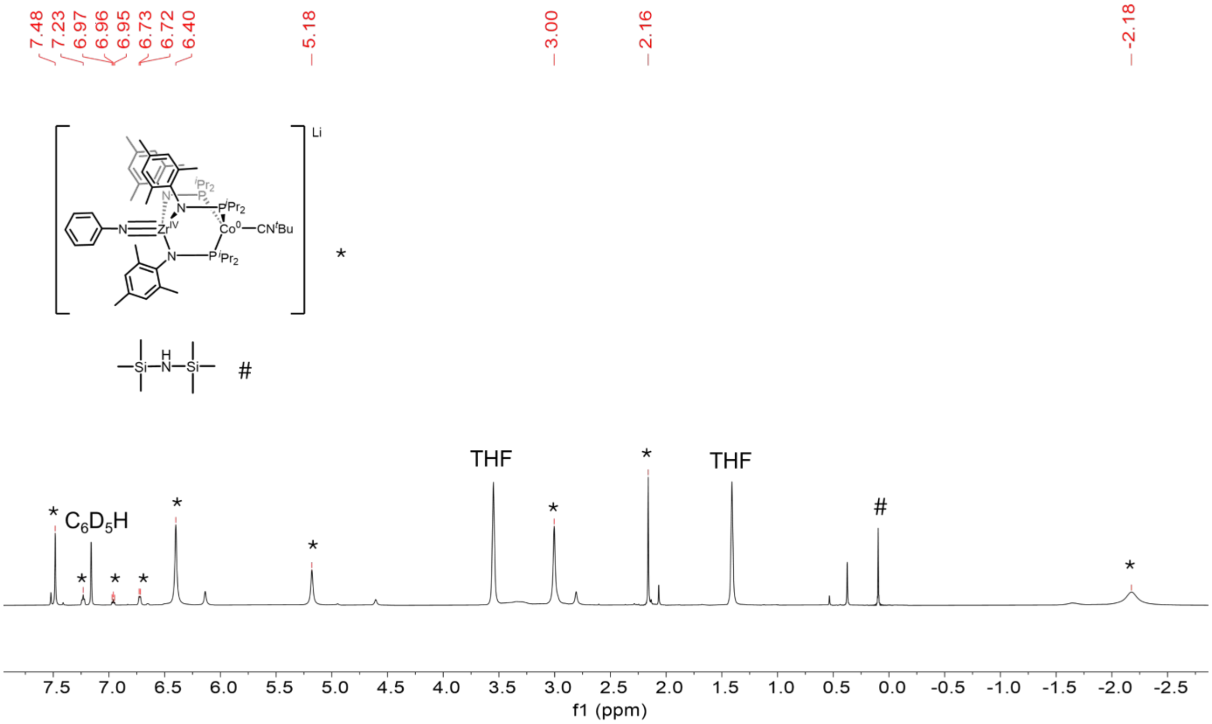

Figure S52. ^{1}H NMR spectrum (600 MHz, C_6D_6) of the crude reaction between **3** and $LiN(SiMe_3)_2$ after 1 h of stirring.

5.4. Reaction of (PhHN)Zr^{IV}(MesNP^{i}Pr$_2$)$_3$Co^{0}CN^{t}Bu (3) with NEt$_3$

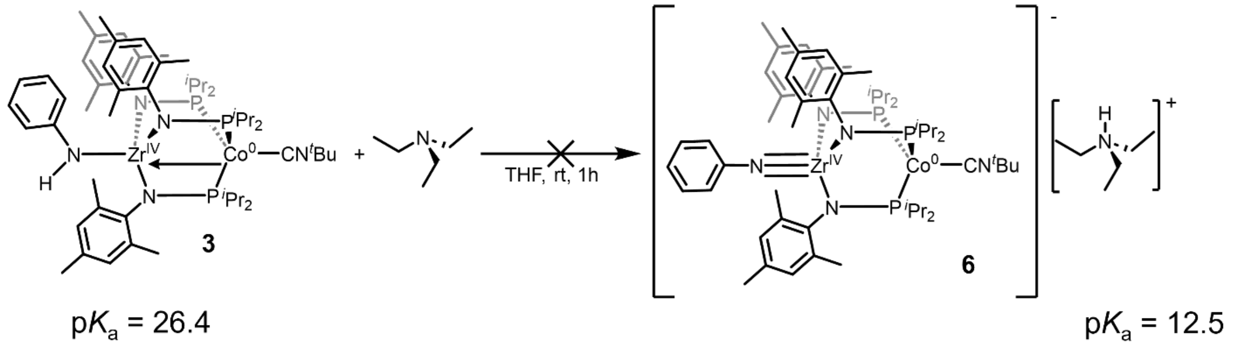

NEt$_3$ (0.94 mg, 0.0093 mmol) was added to a stirring solution of **3** (9.9 mg, 0.0092 mmol) in THF (~2 mL). There was no color change observed during the reaction; the solution remained orange-red. The reaction mixture was allowed to stir at room temperature for 1 h. The volatile components were removed under vacuum prior to ^{1}H NMR analysis. The resulting ^{1}H NMR spectrum revealed no reaction.

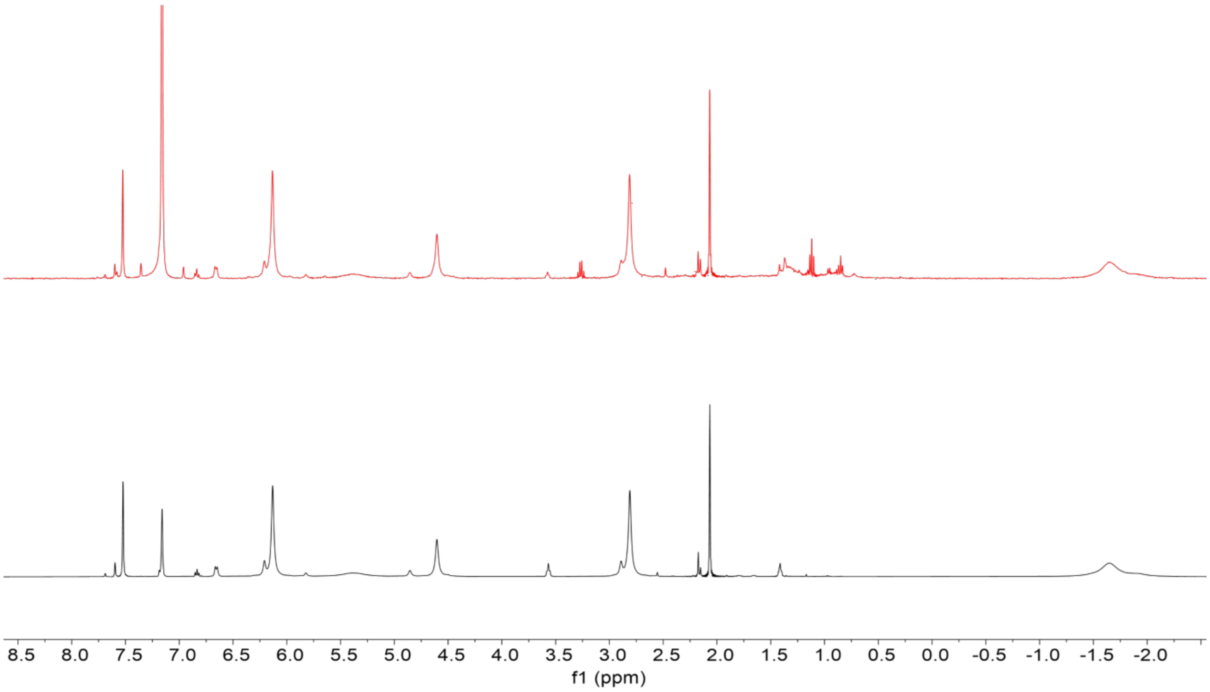

Figure S53. ^{1}H NMR spectra (400 MHz, C_6D_6) of **3** (top, red) and the crude reaction between **3** and NEt$_3$ after 1 h of stirring (bottom, black).

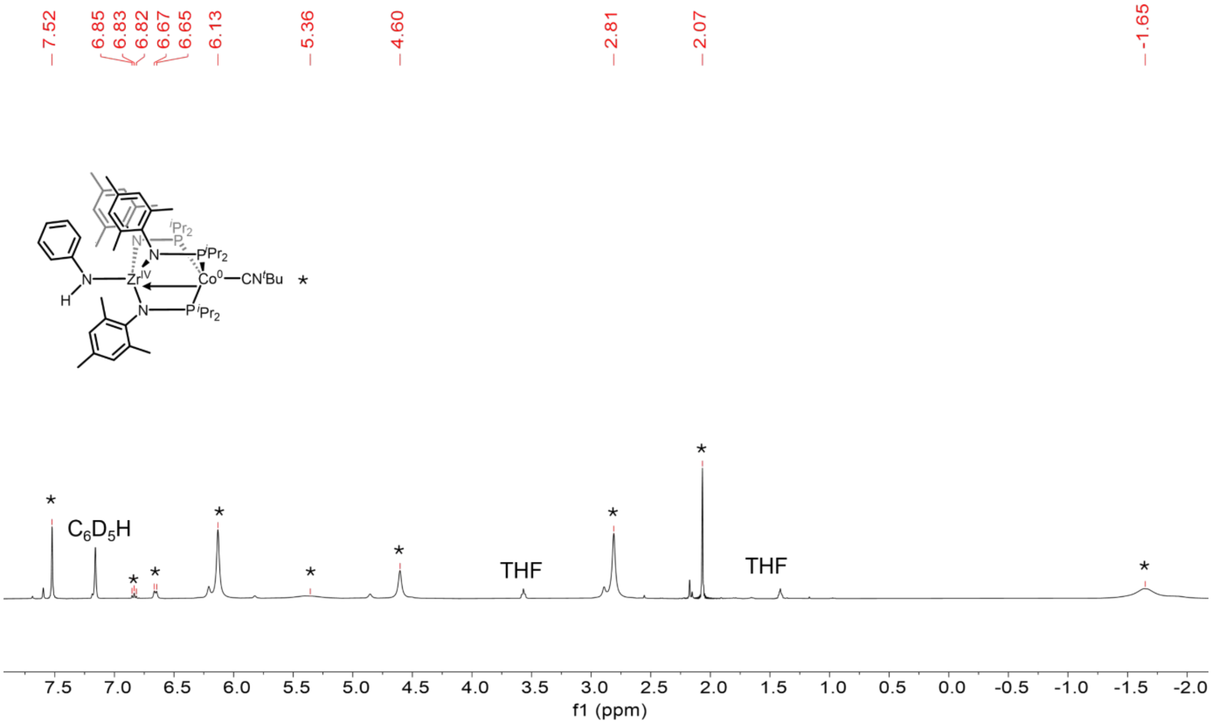

Figure S54. ^{1}H NMR spectrum (400 MHz, C_6D_6) of the crude reaction between **3** and NEt_3 after 1 h of stirring.

pK_a [PhHNZr^{IV}(MesNP^{i}Pr$_2$)$_3$Co^{I}CN^{t}Bu][BPh$_4$] (7) = 13.3

5.5. Reaction of [PhNZr^{IV}(MesNP^{i}Pr$_2$)$_3$Co^{I}CN^{t}Bu] (4) with [HNEt$_3$][BPh$_4$]

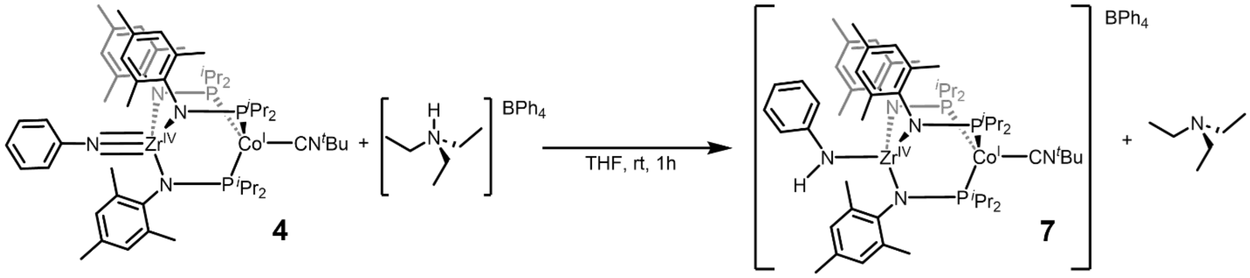

pK_a = 12.5 pK_a = 13.3

[HNEt$_3$][BPh$_4$] (4.7 mg, 0.011 mmol) was dissolved in THF (~2 mL) and added to **4** (11.3 mg, 0.00105 mmol) with stirring. The reaction mixture changed from bright green to a yellow color. The reaction mixture was allowed to stir at room temperature for 1 hour. The volatiles were removed under vacuum prior to ^{1}H NMR analysis.

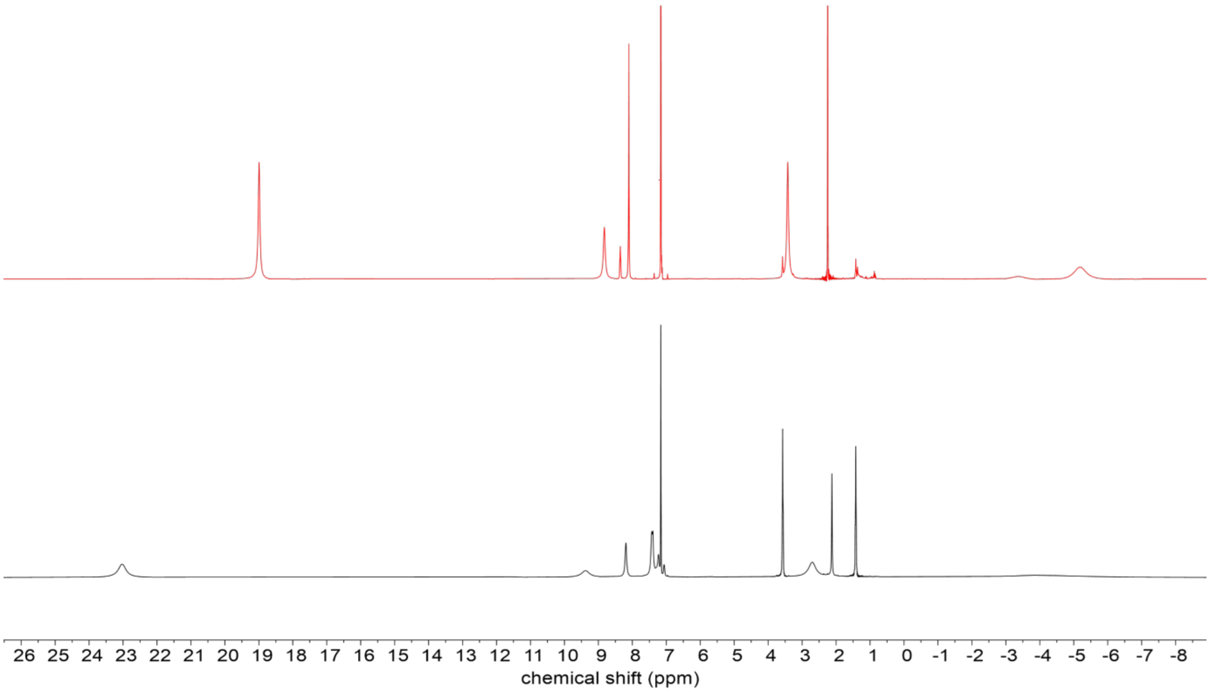

Figure S55. ^{1}H NMR spectra (400 MHz, C_6D_6) of **4** (top, red) and the crude reaction between **4** and $[HNEt_3][BPh_4]$ after 1 h of stirring (bottom, black).

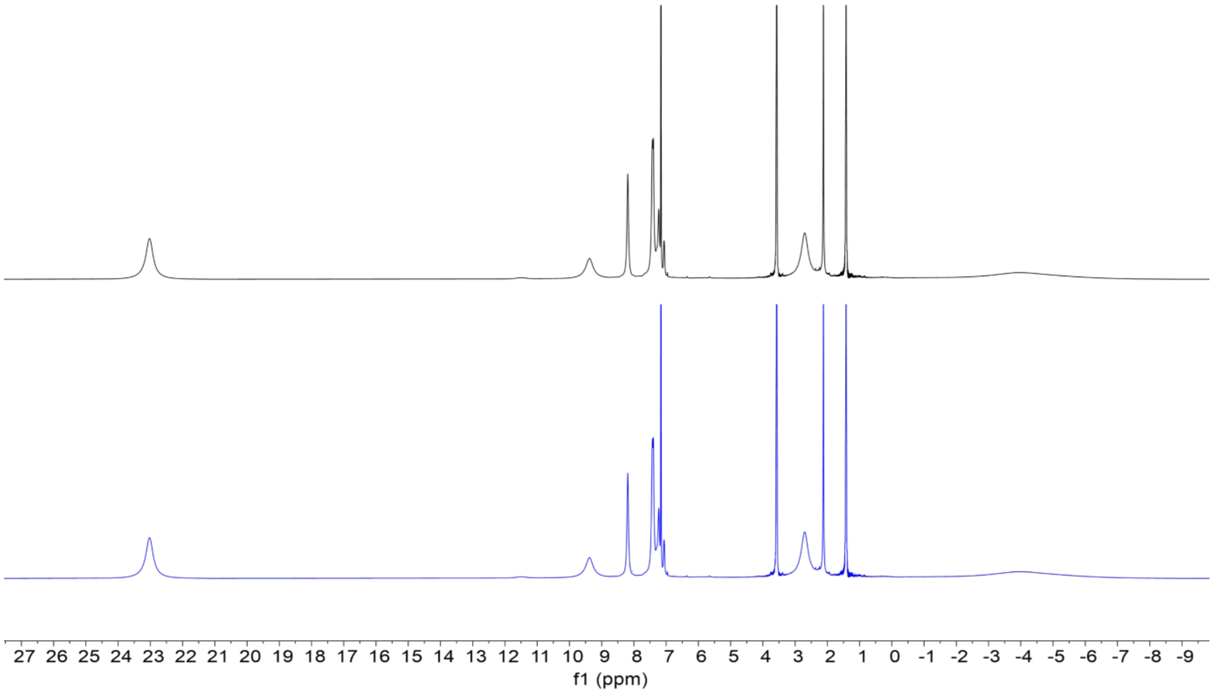

Figure S56. ^{1}H NMR spectra (400 MHz, C_6D_6) of the crude reaction between **4** and $[HNEt_3][BPh_4]$ after 1 h of stirring (top, black) and the isolated desired product **7** (bottom, blue).

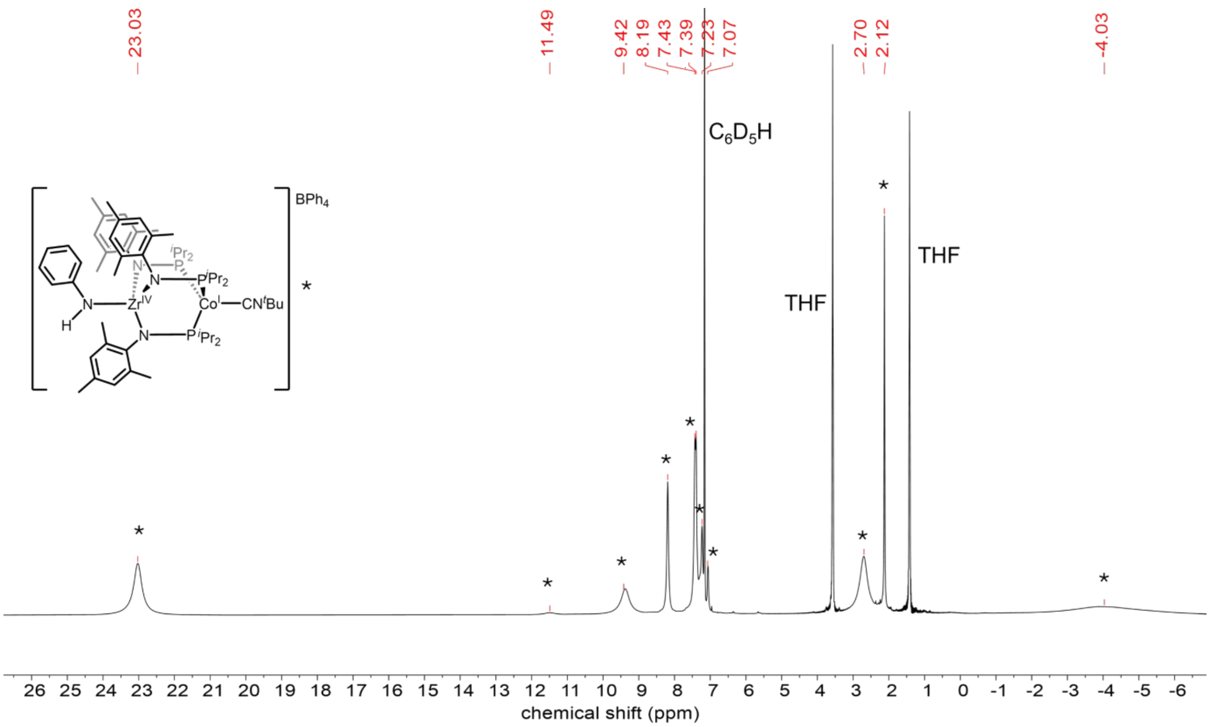

Figure S57. ^{1}H NMR spectrum (400 MHz, C_6D_6) of the crude reaction between **4** and [$HNEt_3$][BPh_4] after 1 h of stirring.

5.6. Reaction between [PhNZr^{IV}(MesNP^{i}Pr$_2$)$_3$Co^{I}CN^{t}Bu] (4) with [^{t}BuHN]P(pyrr)[BPh$_4$]

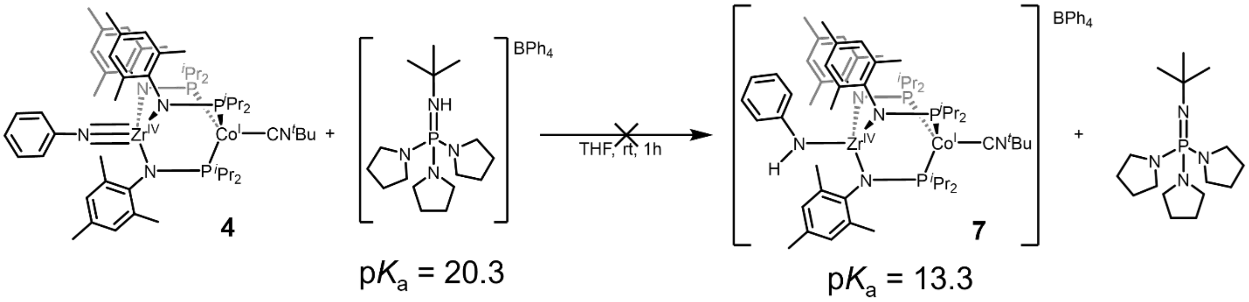

[^{t}BuHN]P(pyrr)[BPh$_4$] (6.8 mg, 0.011 mmol) was added to a stirring solution of **4** (10.0 mg, 0.00930 mmol) in THF (~2 mL). There was no color change observed during the reaction; the solution remained green. The reaction mixture was allowed to stir at room temperature for 1 h. The THF was removed under vacuum prior to ^{1}H NMR analysis. The resulting ^{1}H NMR spectrum revealed no reaction.

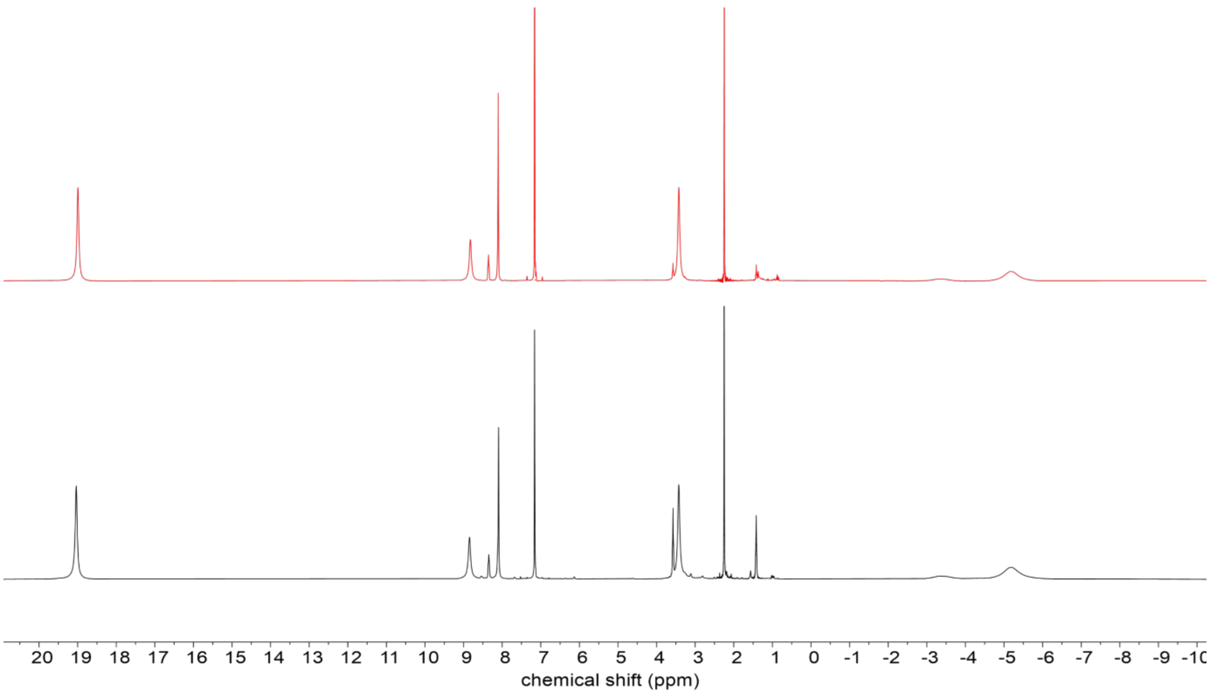

Figure S58. ^{1}H NMR spectra (400 MHz, C_6D_6) of **4** (top, red) and the crude reaction between **4** and [^{t}BuHN]P(pyrr)[BPh_4] after 1 h of stirring (bottom, black).

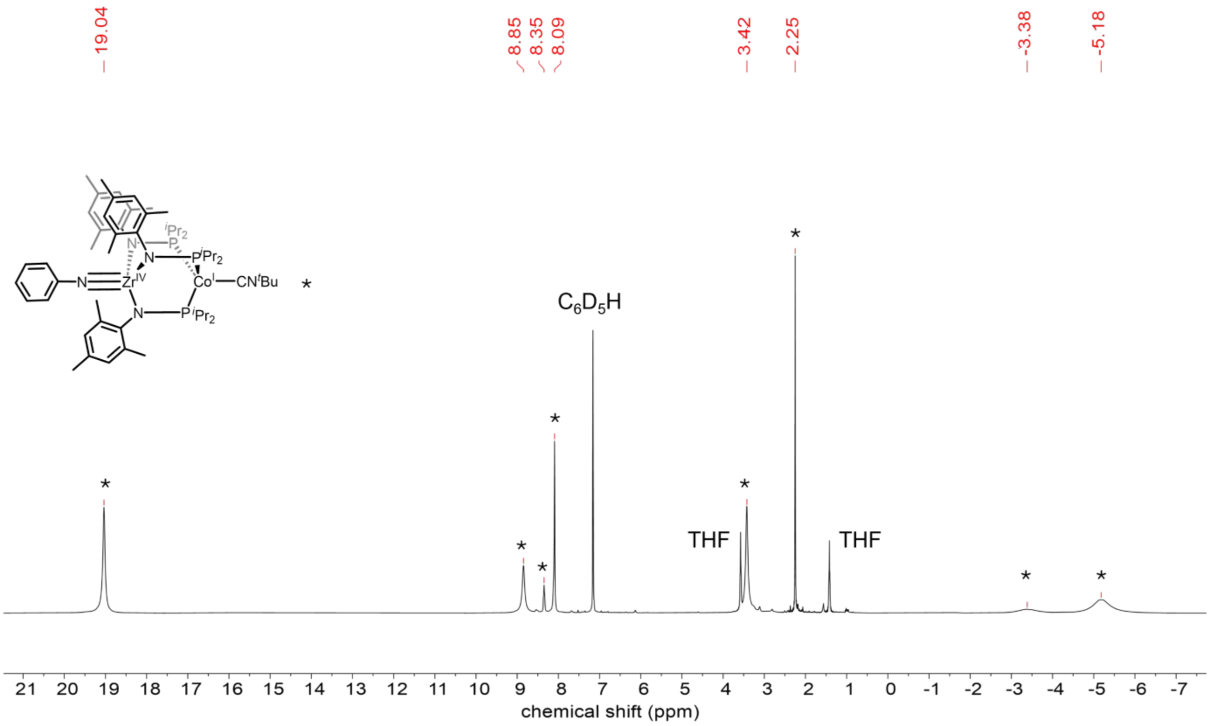

Figure S59. ^{1}H NMR spectrum (400 MHz, C_6D_6) of the crude reaction between **4** and [^{t}BuHN]P(pyrr)[BPh_4] after 1 h of stirring.

6. Crystallographic Data

6.1 Crystallographic Data of $(PhH_2N)Zr^{IV}(MesNP^iPr_2)_3Co^{-I}CN^tBu$ (2)

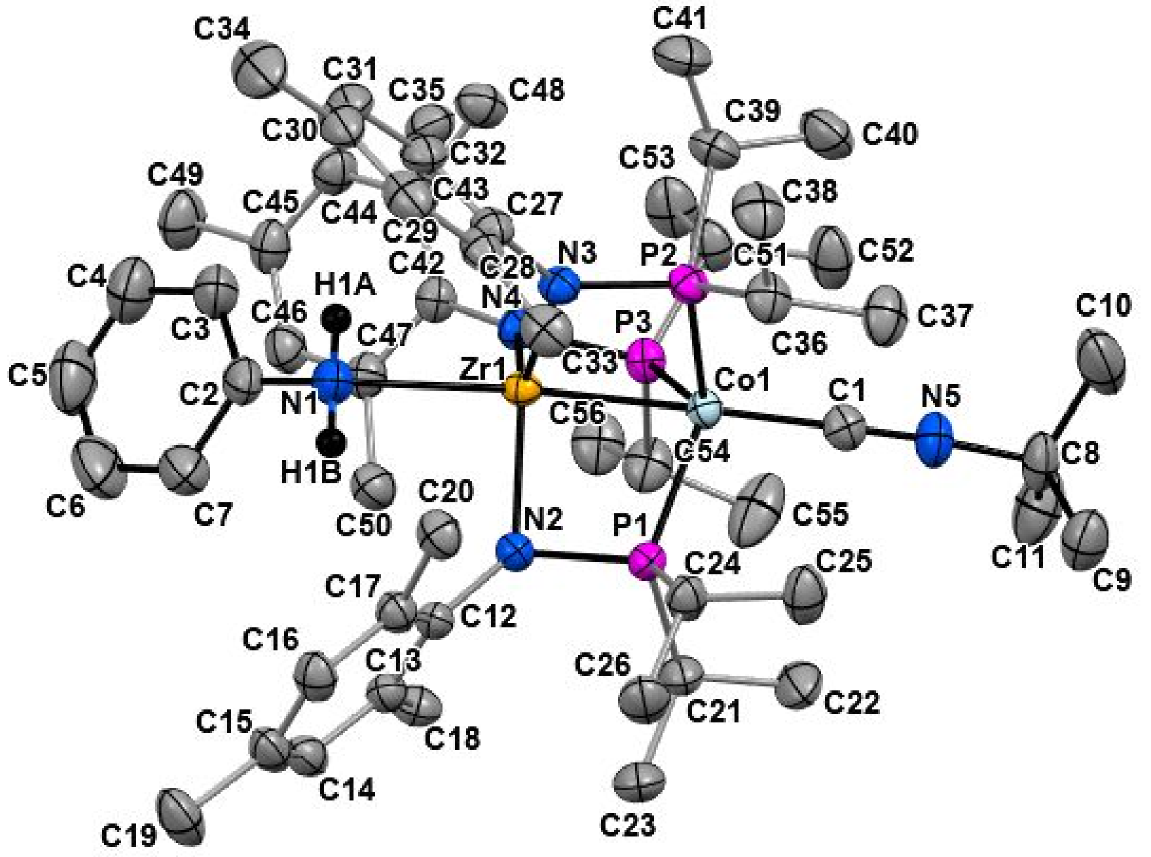

Figure S60. Fully labeled ellipsoid representation (50%) of **2**.

Experimental summary

The single crystal X-ray diffraction studies were carried out on a Bruker Kappa Photon III CPAD diffractometer equipped with Mo K_α radiation (λ = 0.71073 Å). A 0.202 x 0.112 x 0.109 mm piece of an orange block was mounted on a MiTeGen MicroMount with Paratone 24EX oil. Data were collected in a nitrogen gas stream at 100(2) K using ϕ and ϖ scans. Crystal-to-detector distance was 60 mm using variable exposure time (5s-30s) depending on θ with a scan width of 0.75°. Data collection was 99.8% complete to 25.00° in θ (0.83 Å). A total of 195607 reflections were collected covering the indices, $-22 \le h \le 22$, $-16 \le k \le 16$, $-29 \le l \le 29$. 11657 reflections were found to be symmetry independent, with a R_{int} of 0.0618. Indexing and unit cell refinement

indicated a primitive, monoclinic lattice. The space group was found to be $P2_1/c$. The data were integrated using the Bruker SAINT software program and scaled using the SADABS software program. Solution by dual-space method (SHELXT) produced a complete phasing model for refinement.

All nonhydrogen atoms were refined anisotropically by full-matrix least-squares (SHELXL-2014). All carbon bonded hydrogen atoms were placed using a riding model. Their positions were constrained relative to their parent atom using the appropriate HFIX command in SHELXL-2014. All other hydrogen atoms (N-bonded) were located in the difference map. Their relative positions were restrained using DFIX commands and their thermals freely refined. Crystallographic data are summarized in Table S1.

6.2. Crystallographic Data of (PhHN)Zr^{IV}(MesNP^{i}Pr$_2$)$_3$Co^{0}CN^{t}Bu (3)

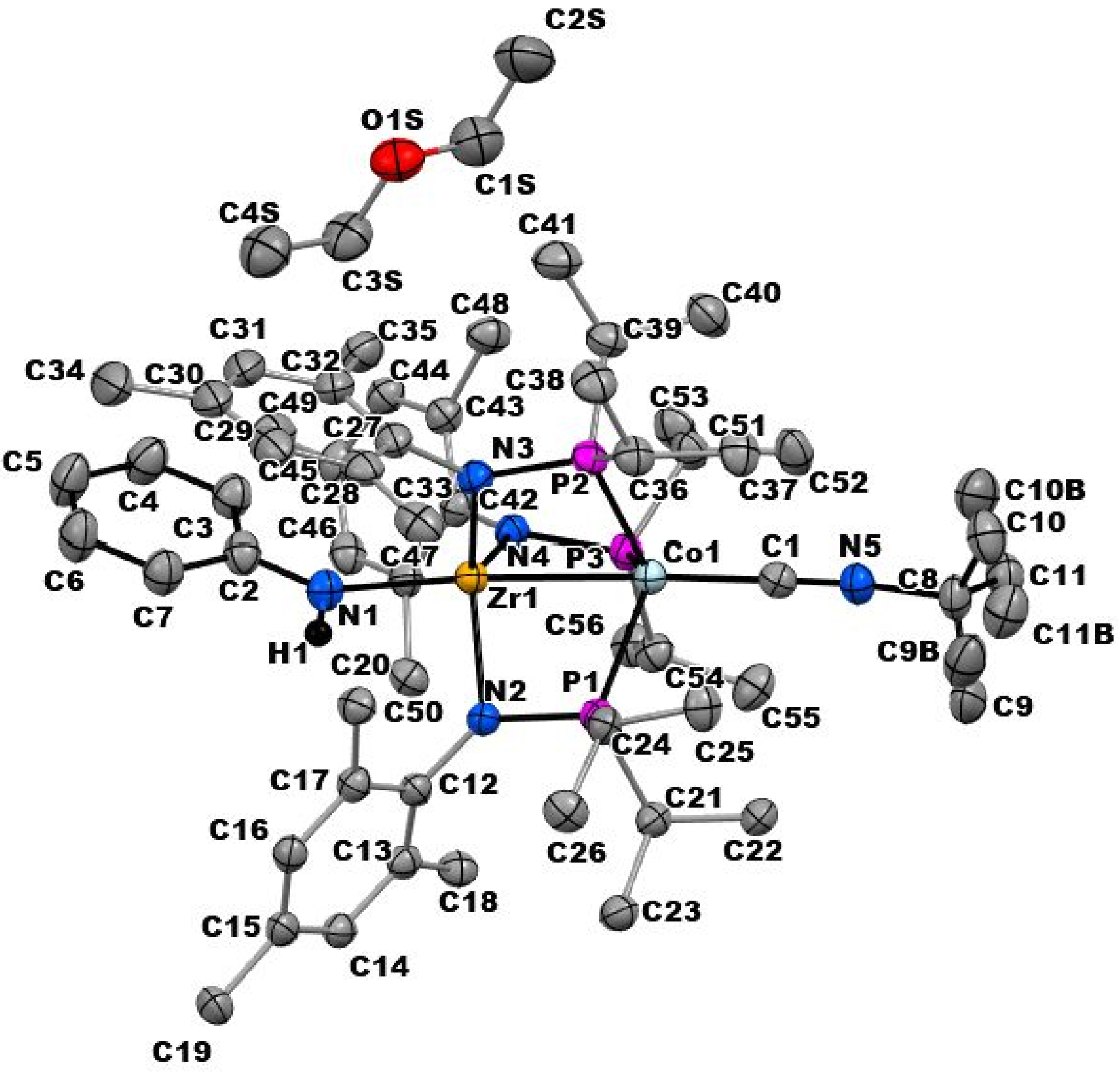

Figure S61. Fully labeled ellipsoid representation (50%) of **3**.

Experimental summary

The single crystal X-ray diffraction studies were carried out on a Bruker Kappa Photon III CPAD diffractometer equipped with Mo K$_\alpha$ radiation (λ = 0.71073 Å). A 0.116 x 0.059 x 0.039 mm piece of an orange plate was mounted on a MiTeGen MicroMount with Paratone 24EX oil. Data were collected in a nitrogen gas stream at 100(2) K using ϕ and ϖ scans. Crystal-to-detector distance was 60 mm using variable exposure time (1s-5s) depending on θ with a scan width of

1.0°. Data collection was 99.9% complete to 25.00° in θ (0.83 Å). A total of 215847 reflections were collected covering the indices, $-29 \leq h \leq 29$, $-26 \leq k \leq 26$, $-28 \leq l \leq 32$. 13549 reflections were found to be symmetry independent, with a R_{int} of 0.0671. Indexing and unit cell refinement indicated a *C*-centered, monoclinic lattice. The space group was found to be *C*2/c. The data were integrated using the Bruker SAINT software program and scaled using the SADABS software program. Solution by dual-space method (SHELXT) produced a complete phasing model for refinement.

All nonhydrogen atoms were refined anisotropically by full-matrix least-squares (SHELXL-2014). All carbon bonded hydrogen atoms were placed using a riding model. Their positions were constrained relative to their parent atom using the appropriate HFIX command in SHELXL-2014. The other hydrogen atom (N-bonded) was located in the difference map. Its relative position was restrained using a DFIX command and its thermal freely refined. Crystallographic data are summarized in Table S1.

6.3. Crystallographic Data of (PhN)Zr^{IV}(MesNP^{i}Pr$_2$)$_3$Co^{I}CN^{t}Bu (4)

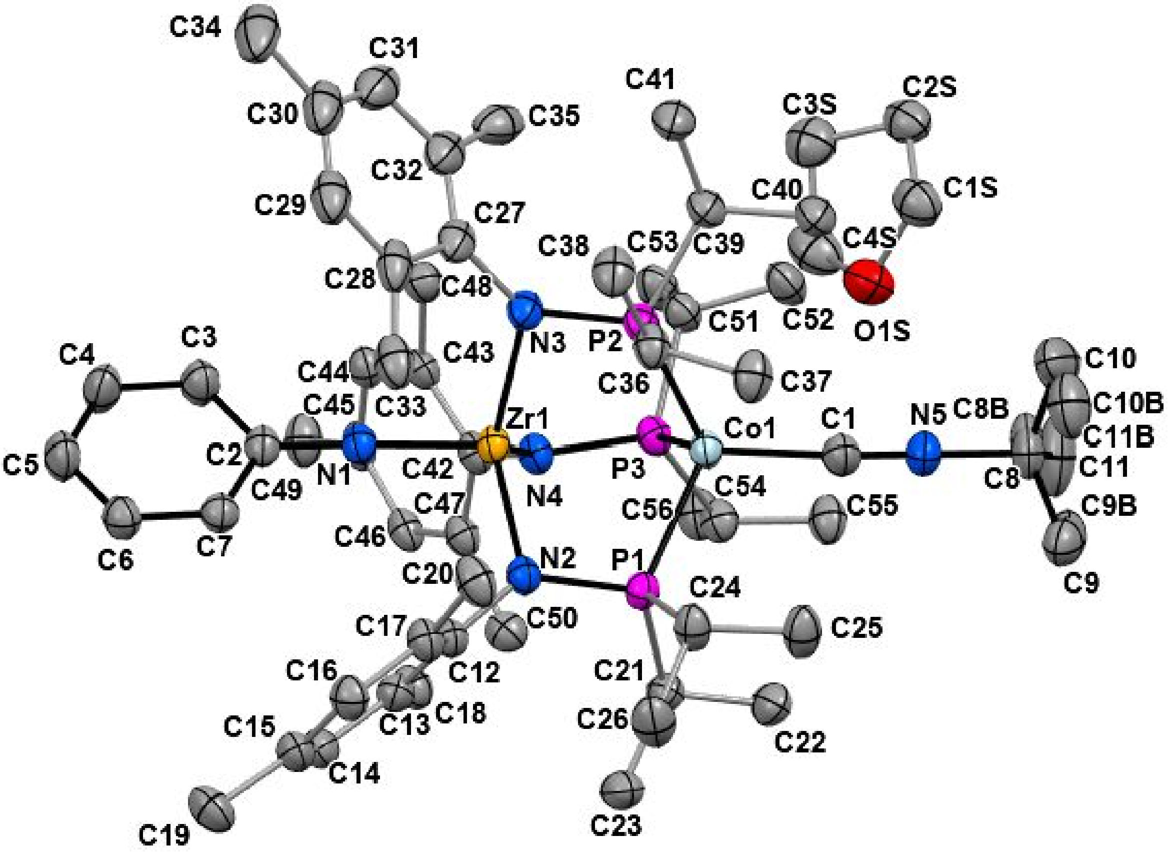

Figure S62. Fully labeled ellipsoid representation (50%) of **4**.

Experimental summary

The single crystal X-ray diffraction studies were carried out on a Bruker Kappa Photon III CPAD diffractometer equipped with Mo K_α radiation (λ = 0.71073 Å). A 0.134 x 0.119 x 0.092 mm piece of a yellow block was mounted on a MiTeGen MicroMount with Paratone 24EX oil. Data were collected in a nitrogen gas stream at 100(2) K using ϕ and ϖ scans. Crystal-to-detector distance was 60 mm using variable exposure time (1s-3s) depending on θ with a scan width of 0.75°. Data collection was 99.9% complete to 25.00° in θ (0.83 Å). A total of 151065 reflections were collected covering the indices, $-28 \le h \le 28$, $-27 \le k \le 26$ $-33 \le l \le 33$. 13552 reflections were

found to be symmetry independent, with a R_{int} of 0.0458. Indexing and unit cell refinement indicated a *C*-centered, monoclinic lattice. The space group was found to be *C*2/c. The data were integrated using the Bruker SAINT software program and scaled using the SADABS software program. Solution by dual-space method (SHELXT) produced a complete phasing model for refinement.

All nonhydrogen atoms were refined anisotropically by full-matrix least-squares (SHELXL-2014). All hydrogen atoms were placed using a riding model. Their positions were constrained relative to their parent atom using the appropriate HFIX command in SHELXL-2014. Crystallographic data are summarized in Table S2.

6.4. Crystallographic Data of [PhHNZr^{IV}(MesNP^{i}Pr$_2$)$_3$Co^{-I}CN^{t}Bu]$^{-}$ (5)

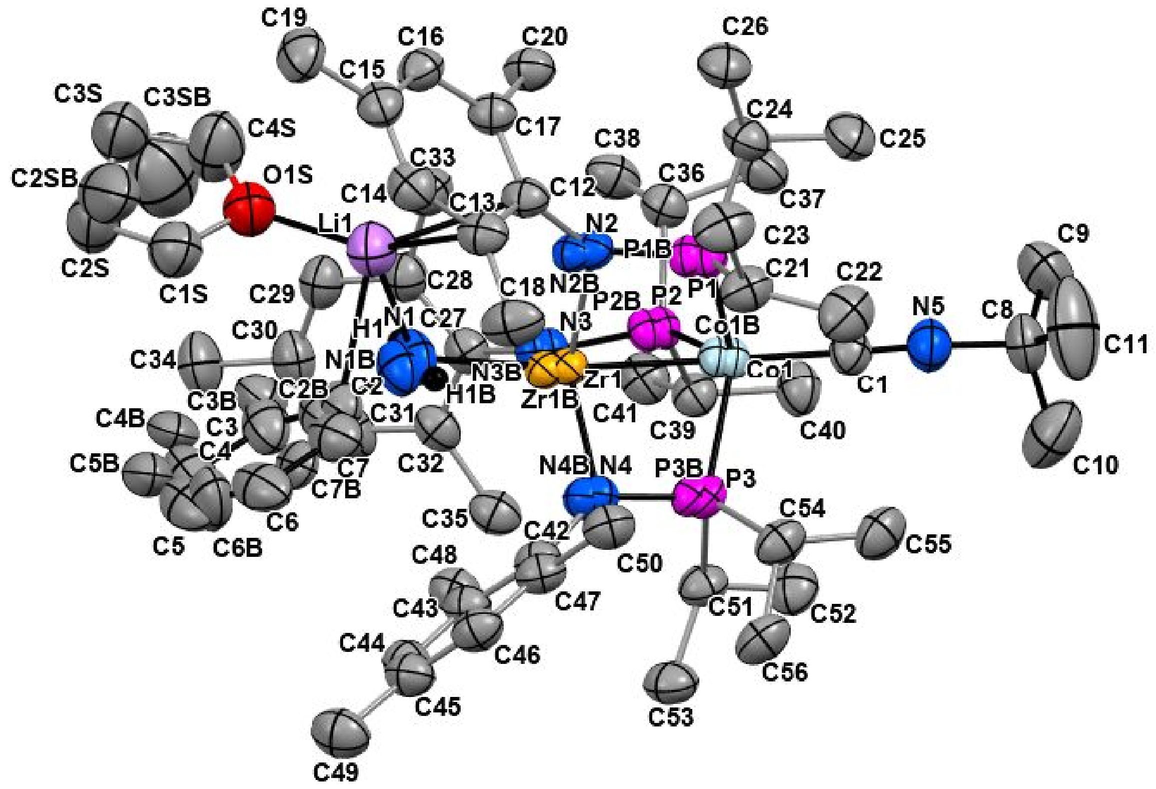

Figure S63. Fully labeled ellipsoid representation (50%) of **5.**

Experimental summary

The single crystal X-ray diffraction studies were carried out on a Bruker Kappa Photon III CPAD diffractometer equipped with Ga K_{α} radiation (λ = 1.34139). A 0.103 x 0.068 x 0.045 mm piece of a yellow block was mounted on a MiTeGen MicroMount with Paratone 24-EX oil. Data were collected in a nitrogen gas stream at 100(2) K using ϕ and ϖ scans. Crystal-to-detector distance was 50 mm using variable exposure time (1s-10s) depending on θ with a scan width of 1.0°. Data collection was 100% complete to 54.00° in *θ* (0.83 Å). A total of 187130 reflections were collected covering the indices, -28 ≤ h ≤ 23, 24 ≤ k ≤ 26, 32 ≤ l ≤ 31. 12558 reflections were found to be symmetry independent, with a R_{int} of 0.0759. Indexing and unit cell refinement indicated a primitive, orthorhombic lattice. The space group was found to be *P*bca. The data were integrated using the Bruker SAINT software program and scaled using the SADABS

software program. Solution by dual-space method (SHELXT) produced a complete phasing model for refinement.

All nonhydrogen atoms were refined anisotropically by full-matrix least-squares (SHELXL-2014). All carbon bonded hydrogen atoms were placed using a riding model. Their positions were constrained relative to their parent atom using the appropriate HFIX command in SHELXL-2014. All other hydrogen atoms (N-bonded) were located in the difference map. Their relative positions were restrained using DFIX commands and their thermals fixed to that of their parent atom. Crystallographic data are summarized in Table S2.

6.5. Crystallographic Data of $[PhNZr^{IV}(MesNP^{i}Pr_2)_3Co^{0}CN^{t}Bu]^-$ (6)

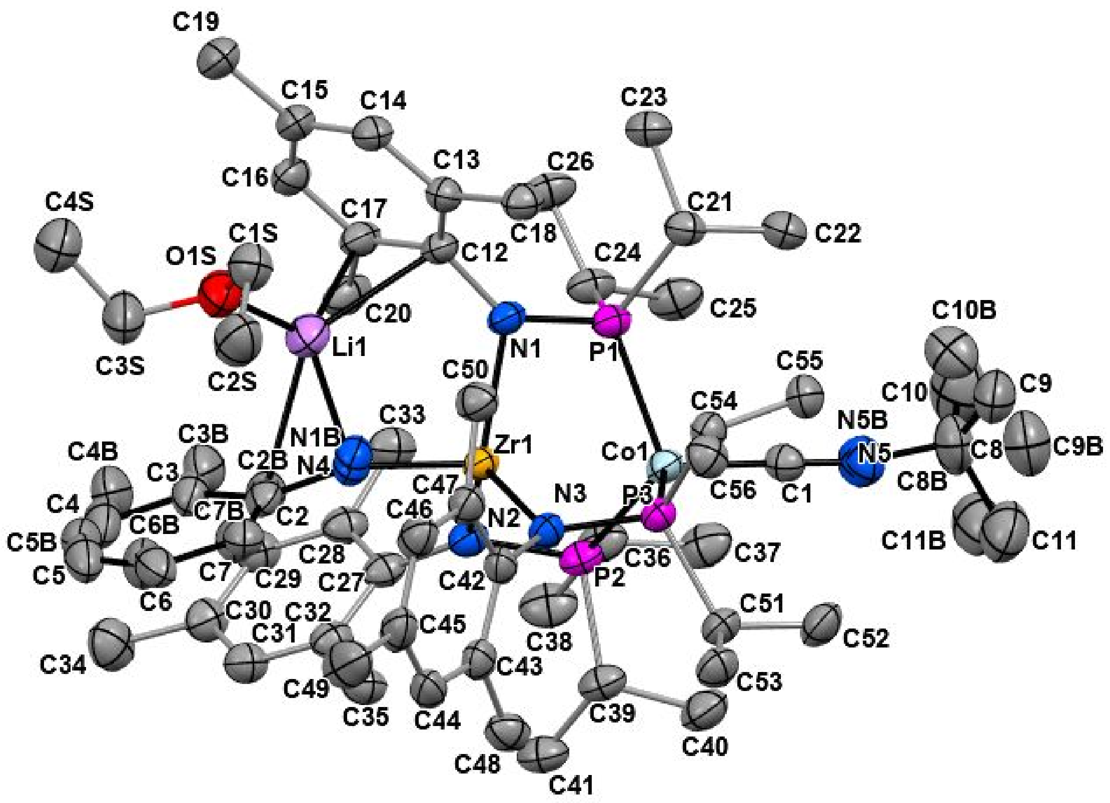

Figure S64. Fully labeled ellipsoid representation (50%) of **6.**

Experimental summary

The single crystal X-ray diffraction studies were carried out on a Bruker Kappa Photon III CPAD diffractometer equipped with Mo K_α radiation (λ = 0.71073 Å). A 0.192 x 0.149 x 0.021 mm piece of a yellow plate was mounted on a MiTeGen MicroMount with Paratone 24EX oil. Data were collected in a nitrogen gas stream at 100(2) K using ϕ and ϖ scans. Crystal-to-detector distance was 60 mm using variable exposure time (1s-10s) depending on θ with a scan width of 1.0°. Data collection was 99.9% complete to 25.00° in θ (0.83Å). A total of 15223 reflections were collected covering the indices, -9 ≤ h ≤ 10, -10 ≤ k ≤ 10, -19 ≤ l ≤ 18. 4110 reflections were found to be symmetry independent, with a R_{int} of 0.0267. Indexing and unit cell refinement indicated a primitive, monoclinic lattice. The space group was found to be $P2_1$. The data were integrated

using the Bruker SAINT software program and scaled using the SADABS software program. Solution by dual-space method (SHELXT) produced a complete phasing model for refinement.

All nonhydrogen atoms were refined anisotropically by full-matrix least-squares (SHELXL-2014). All hydrogen atoms were placed using a riding model. Their positions were constrained relative to their parent atom using the appropriate HFIX command in SHELXL-2014. Crystallographic data are summarized in Table S3.

6.6. Crystallographic Data of [PhHNZr^{IV}(MesNP^{i}Pr$_2$)$_3$Co^{I}CN^{t}Bu][BPh$_4$] (7)

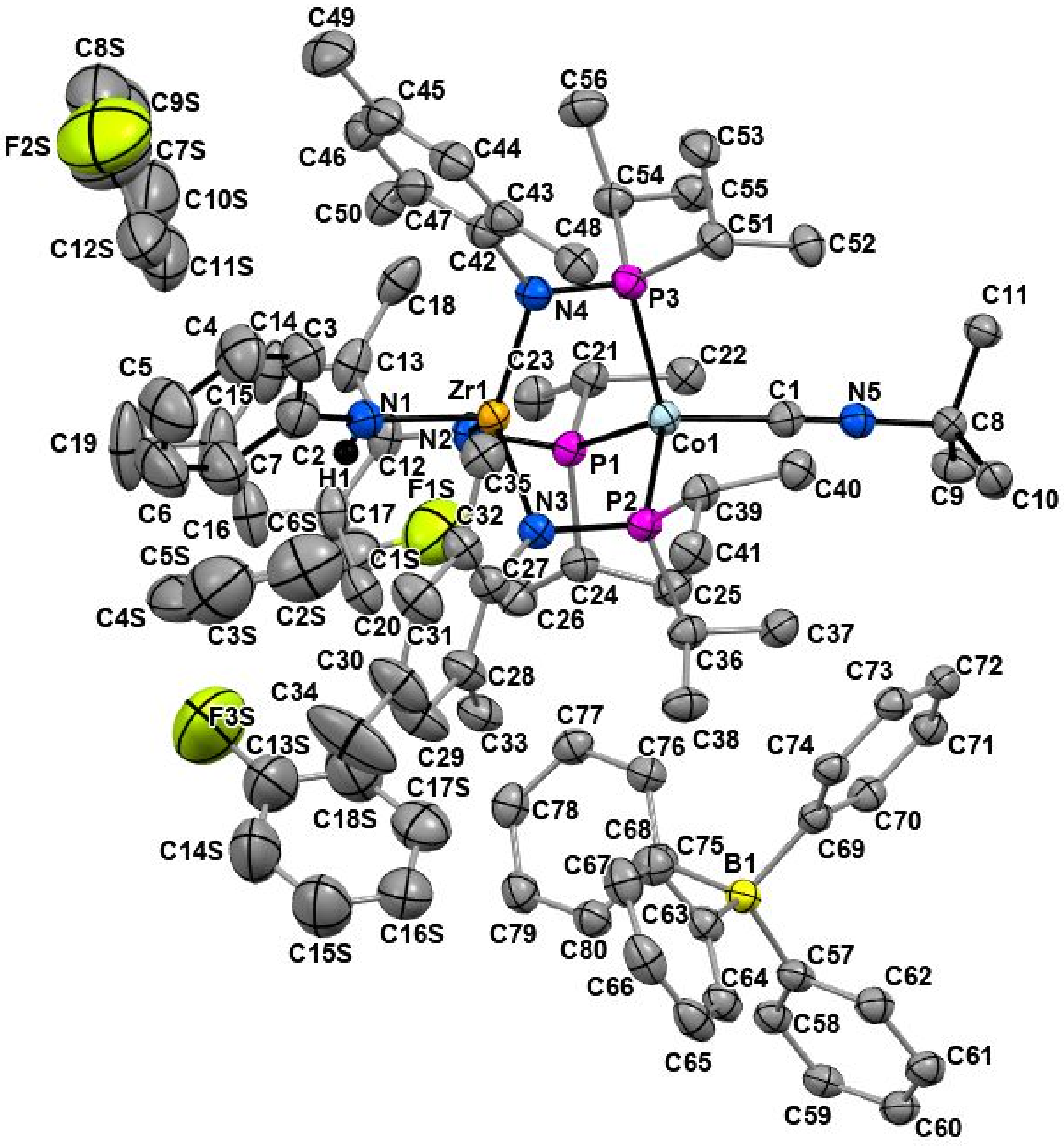

Figure S65. Fully labeled ellipsoid representation (50%) of **7.**

Experimental summary

The single crystal X-ray diffraction studies were carried out on a Bruker Kappa Photon III CPAD diffractometer equipped with Mo K$_{\alpha}$ radiation (λ = 0.71073 Å). A 0.317 x 0.195 x 0.040 mm

piece of a yellow plate was mounted on a MiTeGen MicroMount with Paratone 24EX oil. Data were collected in a nitrogen gas stream at 100(2) K using ϕ and ϖ scans. Crystal-to-detector distance was 100 mm using variable exposure time (2s-20s) depending on θ with a scan width of 0.75°. Data collection was 99.9% complete to 25.00° in θ (0.83 Å). A total of 163715 reflections were collected covering the indices, $-33 \leq h \leq 32$, $-19 \leq k \leq 19$, $-27 \leq l \leq 27$. 18383 reflections were found to be symmetry independent, with a R_{int} of 0.0593. Indexing and unit cell refinement indicated a primitive, monoclinic lattice. The space group was found to be $P2_1/c$. The data were integrated using the Bruker SAINT software program and scaled using the SADABS software program. Solution by dual-space method (SHELXT) produced a complete phasing model for refinement.

All nonhydrogen atoms were refined anisotropically by full-matrix least-squares (SHELXL-2014). All carbon bonded hydrogen atoms were placed using a riding model. Their positions were constrained relative to their parent atom using the appropriate HFIX command in SHELXL-2014. The other hydrogen atom (N-bonded) was located in the difference map. Its relative position was restrained using a DFIX command and its thermal freely refined. Crystallographic data are summarized in Table S3.

Table S1. Crystal data and structure refinement for **2** and **3.**

	**2**	**3•Et$_2$O**
CCDC Number	2505707	2505710
Empirical Formula	$C_{56}H_{91}CoN_5P_3Zr$	$C_{60}H_{100}CoN_5OP_3Zr$
Molecular Formula	$C_{56}H_{91}CoN_5P_3Zr$	$C_{56}H_{90}CoN_5P_3Zr$, C_4 $H_{10}O$
Formula Weight (g/mol)	1077.39	1150.50
Temperature (K)	100.0	100.0
Wavelength (Å)	0.71073	0.71073
Crystal System	Monoclinic	Monoclinic
Space Group	$P2_1/c$	$C2/c$
a (Å)	17.9987(14)	23.0374(15)
b (Å)	13.3379(9)	21.0437(13)
c (Å)	23.6523(14)	25.4194(13)
α (°)	90	90
β (°)	91.674(2)	94.264(2)
γ (°)	90	90
Volume (Å^3)	5675.7(7)	12289.0(13)
Z	4	8
Density (calculated) (g/cm^3)	1.261	1.244
Absorption Coefficient (mm^{-1})	0.600	0.559
F(000)	2296	4920
Crystal Size (mm^3)	0.202 x 0.112 x 0.109	0.116 x 0.059 x 0.039
Crystal Color, Habit	orange block	orange plate
Final R Indices [$I>2\sigma(I)$]	R_1 = 0.0309, wR_2 = 0.0752	R_1 = 0.0345, wR_2 = 0.0840
R Indices (all data)	R_1 = 0.0446, wR_2 = 0.0838	R_1 = 0.0483, wR_2 = 0.0922

Table S2. Crystal data and structure refinement for **4** and **5.**

	**4•THF**	**5•THF**
CCDC Number	2505706	2505711
Empirical Formula	$C_{60}H_{97}CoN_5OP_3Zr$	$C_{60}H_{98.57}CoLiN_5OP_3Zr$
Molecular Formula	$C_{56}H_{89}CoN_5P_3Zr$, C_4H_8O	$C_{60}H_{98.57}CoLiN_5OP_3Zr$
Formula Weight (g/mol)	1147.48	1156.01
Temperature (K)	100.0	100.00
Wavelength (Å)	0.71073	1.34139
Crystal System	Monoclinic	Orthorhombic
Space Group	C2/c	Pbca
a (Å)	22.3739(10)	22.7094(13)
b (Å)	21.0645(8)	20.9404(12)
c (Å)	26.0552(11)	25.8325(16)
α (°)	90	90
β (°)	92.920	90
γ (°)	90	90
Volume ($Å^3$)	12263.7(9)	12284.5(13)
Z	8	8
Density (calculated) (g/cm^3)	1.243	1.250
Absorption Coefficient (mm^{-1})	0.560	3.140
F(000)	4896	4933
Crystal Size (mm^3)	0.134 x 0.119 x 0.092	0.103 x 0.068 x 0.045
Crystal Color, Habit	yellow block	yellow block
Final R Indices [I>2σ(I)]	R_1 = 0.0338, wR_2 = 0.0878	R_1 = 0.0558, wR_2 = 0.1492
R Indices (all data)	R_1 = 0.0424, wR_2 = 0.0957	R_1 = 0.0723, wR_2 = 0.1581

Table S3. Crystal data and structure refinement for **6** and **7.**

	**6•Et$_2$O**	**7•3FPh**
CCDC Number	2505709	2505708
Empirical Formula	$C_{60}H_{99}CoLiN_5OP_3Zr$	$C_{98}H_{125}BCoF_3N_5P_3Zr$
Molecular Formula	$C_{60}H_{99}CoLiN_5OP_3Zr$	$C_{56}H_{90}CoN_5P_3Zr$, $C_{24}H_{20}B$, 3(C_6H_5F)
Formula Weight (g/mol)	1156.44	1683.89
Temperature (K)	100.00	100.0
Wavelength (Å)	0.71073	0.71073
Crystal System	Orthorhombic	Monoclinic
Space Group	*P*bca	$P2_1/c$
a (Å)	22.2806(6)	26.4014(11)
b (Å)	21.2416(7)	15.9075(6)
c (Å)	25.9462(8)	22.0291(9)
α (°)	90	90
β (°)	90	103.6910(10)
γ (°)	90	90
Volume (Å^3)	12279.7(6)	8988.9(6)
Z	8	4
Density (calculated) (g/cm^3)	1.251	1.244
Absorption Coefficient (mm^{-1})	0.560	0.408
F(000)	4936	3568
Crystal Size (mm^3)	0.192 x 0.149 x 0.021	0.317 x 0.195 x 0.040
Crystal Color, Habit	yellow plate	yellow plate
Final R Indices [I>2σ(I)]	R_1 = 0.0418, wR_2 = 0.0991	R_1 = 0.0642, wR_2 = 0.1729
R Indices (all data)	R_1 = 0.0689, wR_2 = 0.1195	R_1 = 0.0711, wR_2 = 0.1783

7. Computational Details

We have previously reported the use of a calibration curve to predict BDFE values with DFT using a series of H atom donors with known experimental values in THF.[1] Upon reconsideration of the calibration curve for this work, it was discovered that the three compounds that deviated most from the linear fit were quinone compounds that are 2 H^+/e^- transfer systems, and the experimentally determined BDFE values are reported as averages of the two H atom transfer steps.[5] Because these three quinones were treated as single H^+/e^- transfer systems in DFT calculations, the data points did not fit in the linear correlation and contributed to significant error in calculated BDFE values.

To eliminate this source of error for the complexes reported here, the calibration curve was reconstructed with the omission of the three quinone compounds that contributed to the poor linear fit and including the addition of two transition metal complexes F and G (Figure S66). Calculated and experimental BDFEs for each complex are tabulated in Table S4.

Free energies of N-H bond homolysis were calculated according to the following equations:

$$\mathbf{X-H \rightarrow X^{\bullet} + H^{\bullet}}$$

$$\mathbf{\Delta G_{BDFE} = (G_{X\cdot} + G_{H\cdot}) - G_{X-H}}$$

The free energy of H• at the present level of theory is -315.1 kcal/mol.

Table S4. List of H• donors with known experimental X-H BDFE values in THF[5] and corresponding DFT-calculated BDFE values in the gas phase.

H· donor	***Experimental X–H BDFE in THF (kcal/mol)***[5]	***X-H BDFE calculated using DFT (kcal/mol)***
A	57.9	65.5
B	71.3	71.1
C	74.4	75.2
D	73.5	76.7
E	83.9	91.8
F	63.6	58.5
G	39.7	45.1
**2**	41.2*	37.0**
**3**	59.6*	58.8**

*Predicted values from the BDFE calibration curve (Figure S66)
** Values determined via direct calculation of ΔG_{BDFE}

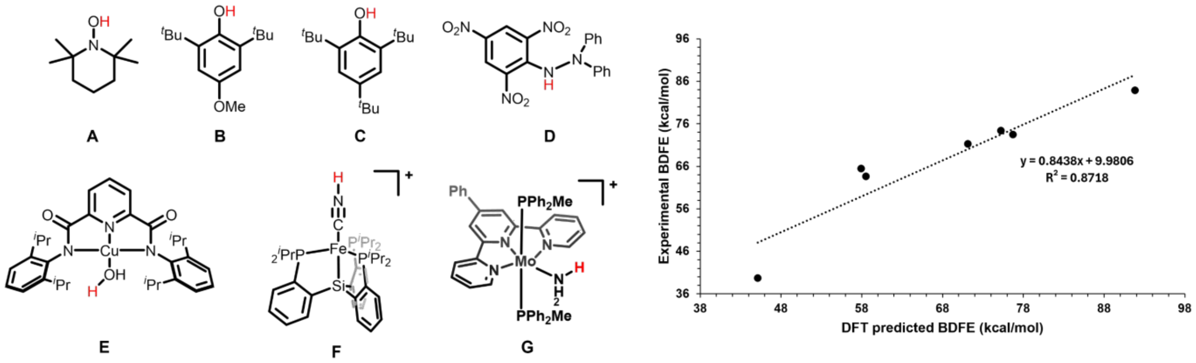

Figure S66. (Left) Structures of seven H• donors used to construct the BDFE calibration curve. (Right) BDFE calibration curve used to estimate BDFE values, plotted with experimental BDFE values in THF versus DFT-calculated BDFE values in the gas phase.

As shown in Table S4, the calibration curve consistently overestimates the BDFE values for **2** and **3** (experimentally determined to be 37 and 55, respectively), while the direct calculation of BDFE more accurately matches experiment. The overestimation of the values for **2** and **3** by the calibration curve is likely due to the scarcity of reported H• donors with BDFE values lower than 60 kcal/mol, and by consequence less accurate calibration of low experimental values like

those reported here. Due to this observed inaccuracy, the calibration curve was omitted and the BDFE values for **2** and **3** were determined *via* direct calculation of ΔG_{BDFE}.

8. References

(1) Feresin, J.; Barden, B. A.; Reyes, J. A.; Abhyankar, P. C.; Barrett, S. M.; Thomas, C. M. Heterobimetallic Multi-Site Concerted Proton Electron Transfer (MS-CPET) Promotes Coordination-Induced O–H Bond Weakening. *Chem. Sci.* **2025**, *16* (28), 12941–12946. https://doi.org/10.1039/D5SC03298A.

(2) Wise, C. F.; Agarwal, R. G.; Mayer, J. M. Determining Proton-Coupled Standard Potentials and X–H Bond Dissociation Free Energies in Nonaqueous Solvents Using Open-Circuit Potential Measurements. *Journal of the American Chemical Society* **2020**, *142* (24), 10681–10691. https://doi.org/10.1021/jacs.0c01032.

(3) Bordwell, F. G.; Cheng, J.; Ji, G. Z.; Satish, A. V.; Zhang, X. Bond Dissociation Energies in DMSO Related to the Gas Phase Values. *J. Am. Chem. Soc.* **1991**, *113* (26), 9790–9795. https://doi.org/10.1021/ja00026a012.

(4) Bordwell, F. G.; Cheng, J. Pei.; Harrelson, J. A. Homolytic Bond Dissociation Energies in Solution from Equilibrium Acidity and Electrochemical Data. *J. Am. Chem. Soc.* **1988**, *110* (4), 1229–1231. https://doi.org/10.1021/ja00212a035.

(5) Agarwal, R. G.; Coste, S. C.; Groff, B. D.; Heuer, A. M.; Noh, H.; Parada, G. A.; Wise, C. F.; Nichols, E. M.; Warren, J. J.; Mayer, J. M. Free Energies of Proton-Coupled Electron Transfer Reagents and Their Applications. *Chem. Rev.* **2022**, *122* (1), 1–49. https://doi.org/10.1021/acs.chemrev.1c00521.

(6) Smith, A. M.; Miller, A. J. M. Open Circuit Potential Method for Thermodynamic Hydricity Measurements of Metal Hydrides. *Organometallics* **2024**, *43* (24), 3163–3170. https://doi.org/10.1021/acs.organomet.4c00144.
